# Red light-emitting short Mango-based system enables tracking a mycobacterial small noncoding RNA in infected macrophages

**DOI:** 10.1093/nar/gkad100

**Published:** 2023-02-25

**Authors:** Oksana S Bychenko, Alexei A Khrulev, Julia I Svetlova, Vladimir B Tsvetkov, Polina N Kamzeeva, Yulia V Skvortsova, Boris S Tupertsev, Igor A Ivanov, Leonid V Aseev, Yuriy M Khodarovich, Evgeny S Belyaev, Liubov I Kozlovskaya, Timofei S Zatsepin, Tatyana L Azhikina, Anna M Varizhuk, Andrey V Aralov

**Affiliations:** Shemyakin-Ovchinnikov Institute of Bioorganic Chemistry, Russian Academy of Sciences, Moscow 117997, Russia; Shemyakin-Ovchinnikov Institute of Bioorganic Chemistry, Russian Academy of Sciences, Moscow 117997, Russia; Federal Research and Clinical Center of Physical-Chemical Medicine, Moscow 119435, Russia; Federal Research and Clinical Center of Physical-Chemical Medicine, Moscow 119435, Russia; Institute of Biodesign and Complex System Modeling, I.M. Sechenov First Moscow State Medical University, Moscow 119991, Russia; Shemyakin-Ovchinnikov Institute of Bioorganic Chemistry, Russian Academy of Sciences, Moscow 117997, Russia; Shemyakin-Ovchinnikov Institute of Bioorganic Chemistry, Russian Academy of Sciences, Moscow 117997, Russia; Skolkovo Institute of Science and Technology, Moscow 121205, Russia; Shemyakin-Ovchinnikov Institute of Bioorganic Chemistry, Russian Academy of Sciences, Moscow 117997, Russia; Shemyakin-Ovchinnikov Institute of Bioorganic Chemistry, Russian Academy of Sciences, Moscow 117997, Russia; Shemyakin-Ovchinnikov Institute of Bioorganic Chemistry, Russian Academy of Sciences, Moscow 117997, Russia; Frumkin Institute of Physical Chemistry and Electrochemistry, Russian Academy of Sciences, Moscow 119071, Russia; FSBSI Chumakov Federal Scientific Center for Research and Development of Immune and Biological Products, Russian Academy of Sciences, Moscow 108819, Russia; Institute of Translational Medicine and Biotechnology I.M. Sechenov First Moscow State Medical University, Moscow 119991, Russia; Lomonosov Moscow State University, Department of Chemistry, Moscow 119992, Russia; Shemyakin-Ovchinnikov Institute of Bioorganic Chemistry, Russian Academy of Sciences, Moscow 117997, Russia; Federal Research and Clinical Center of Physical-Chemical Medicine, Moscow 119435, Russia; Center for Precision Genome Editing and Genetic Technologies for Biomedicine, Federal Research and Clinical Center of Physical-Chemical Medicine of Federal Medical Biological Agency, Moscow 119435, Russia; Shemyakin-Ovchinnikov Institute of Bioorganic Chemistry, Russian Academy of Sciences, Moscow 117997, Russia

## Abstract

Progress in RNA metabolism and function studies relies largely on molecular imaging systems, including those comprising a fluorogenic dye and an aptamer-based fluorescence-activating tag. G4 aptamers of the Mango family, typically combined with a duplex/hairpin scaffold, activate the fluorescence of a green light-emitting dye TO1-biotin and hold great promise for intracellular RNA tracking. Here, we report a new Mango-based imaging platform. Its key advantages are the tunability of spectral properties and applicability for visualization of small RNA molecules that require minimal tag size. The former advantage is due to an expanded (green-to-red-emitting) palette of TO1-inspired fluorogenic dyes, and the truncated duplex scaffold ensures the latter. To illustrate the applicability of the improved platform, we tagged *Mycobacterium tuberculosis* sncRNA with the shortened aptamer-scaffold tag. Then, we visualized it in bacteria and bacteria-infected macrophages using the new red light-emitting Mango-activated dye.

## Graphical Abstract

**Graphical Abstract:**
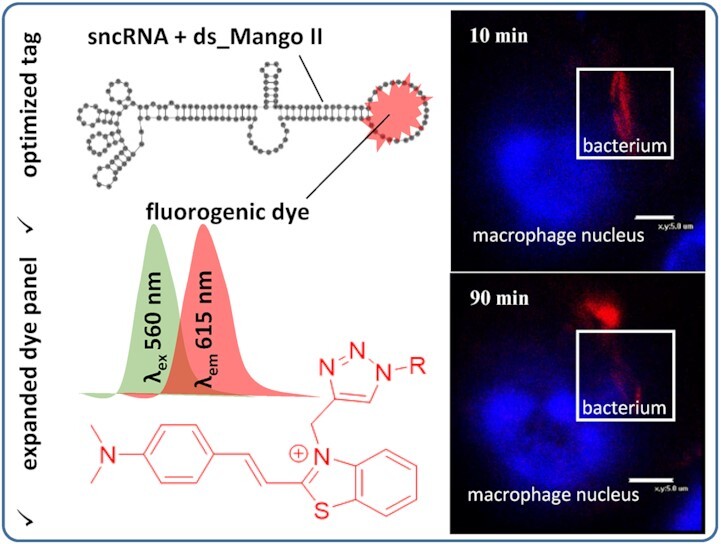
Tag-activated fluorogenic dyes with tunable spectral properties were obtained by a new synthetic approach. A very short aptamer-based tag for labeling short RNA was constructed. The developed fluorogenic dye-tag system was used to visualize target RNA in bacteria internalized by macrophages.

## INTRODUCTION

Small noncoding RNA (sncRNA) have recently emerged as essential modulators of transcription and translation. Many eukaryotic sncRNA are differentially expressed in cancer (1,2), neurodegenerative diseases (3,4) and other pathologies, which makes them attractive drug targets or biomarkers. Prokaryotic snсRNAs govern stress response, adaptation, signaling, and virulence-related processes (5). Of particular interest is their contribution to immune evasion (6). The underlying mechanisms require clarification, hence the necessity for sncRNA imaging and tracking in living cells.

Despite recent advances in encoding fluorogenic RNA or intracellular RNA labeling for fluorescent microscopy imaging (7,8), available methods can hardly be tuned for sncRNA. The critical challenge stems from the size factor. Lengthy/bulky tags, such as arrays of fluorescent protein binding sites, may interfere with sncRNA interactions, traffic, or phase transitions. Thus, in addition to general requirements, such as genetic encoding and biocompatibility, being covalently attachable to RNA of interest, and significant fluorescence with tunable spectral properties, a perfect tag for sncRNA labeling should also be shorter than or at least comparable to a wild-type small RNA (40–500 nt). A more general problem to be solved is contrast enhancement. Systems based on genetically encoded RNA-binding fluorescent proteins suffer from a substantial background signal, encouraging the development of light-up alternatives. Finally, orthogonal dual-color or multiplex imaging systems can track two or more distinct RNAs simultaneously.

Most of the above challenges and issues have been addressed to some extent in current imaging systems based on an aptamer and a fluorogenic dye (9). Such systems comprise a small molecule dye, which is supposed to be non-fluorescent in a free state, and a dye-specific ‘turn-on’ RNA aptamer fused with RNA of interest. In robust imaging systems, intracellular formation of the aptamer–dye complex causes an increase in the fluorescence quantum yields up to several orders of magnitude. SELEX has provided a plethora of aptamers that meet the above ‘turn-on’ criterion and show high specificity for the respective dye, including Spinach, Broccoli, Corn, Chilli, Mango, Coral, Malachite Green, Riboglow, Pepper and Peach (10–18). However, each system suffers more or less pronounced drawbacks associated with complex synthesis, low fluorescence enhancement, inability to penetrate cell membranes, low stability in live cells, prolonged length of the tag and limitations on detection channels.

The Mango-based systems look promising since they meet most requirements for a perfect tag. The first selected aptamer of the Mango family, Mango I, had a relatively small size and very high affinity (*K*_D_ = 3.2 nM) to the cognate thiazole orange (TO)-derived dye TO1-biotin. The dye showed up to 1100-fold fluorescence enhancement upon binding to Mango I aptamer (19). The properties of the proposed system allowed for visualizing TO1-biotin-Mango-I complex in *C. elegans* and fluorescent labeling or purifying biologically relevant RNAs (19). FRET-based conformational sensor and a genetically encodable RNA apta-FRET system that responded to RNA conformational changes in *E. coli* (20) were built based on Mango I aptamer complex with YO3-biotin (a TO1-biotin analog designed to increase the overlap of the FRET donor and acceptor spectra), and Spinach aptamer with its cognate dye DFHBI-1T. Mango I has recently been used to create sensors for fluorescence imaging of miRNA activities in living cells (21,22). The crystal structure of the TO1-biotin-Mango I complex was analyzed at·1.7 Å-resolution, revealing a mostly parallel three-tetrad G-quadruplex (G4) and an A-form helix were linked flexibly through an interrupted GAAA tetraloop-like junction. The fluorogenic core of TO1-biotin was sandwiched between one of the outer G4 tetrads and two unpaired adenines from propeller loops with a 45° angle between the N-methylquinolinyl (MQ) moiety and the benzothiazolium (BT) group leading to the suboptimal conformation responsible for the relatively low quantum yield of the complex (23).

To improve the properties of aptamer-based systems, three new representatives of the Mango family, namely Mango II, III and IV, were selected using a droplet-based microfluidics platform in presence of two competitors, NMM (*N*-methyl mesoporphyrin IX) and TO3-Biotin (24). These Mango aptamers showed high thermal stability, affinity, TO1-biotin fluorescence enhancement, resistance to high Mg^2+^ concentrations, and an ability to withstand formaldehyde fixation. Proposed fluorescent tags allowed fixed- and live-cell imaging of sncRNAs, such as 5S, U6 and a box C/D scaRNA. Tandem Mango II arrays demonstrated applicability for RNA imaging with high contrast and single-molecule sensitivity (25). These arrays of aptamers did not affect the cellular localization of both coding (β-actin mRNA) and long non-coding (NEAT1) RNAs. Moreover, they allowed tracking single mRNAs over an extended period, likely due to replacing the fluorophore after its bleaching. Despite the lack of information on the applicability of the Mango III-based tag in cells, it was used as an acceptor with the Broccoli-based fluorescent label as a donor to measure the angular dependence of FRET *in cuvette* (26). In addition, a sensor containing Mango III variant built from l-ribose units that presumably render the structure stable in cells provided the detection of miRNA-155 in living cells (27). Finally, the FRET pair consisting of DFHBI-1T-iSpinach and YO3-biotin-Mango IV linked by an A-form helix of 10, 11 or 12 bp, was also studied by biophysical methods (28).

The co-crystal study of the TO1-biotin-Mango-II complex revealed significant similarity to the Mango I complex, with some differences stemming from the presence of additional adenines in the loops of Mango II (29). The accompanying structural rearrangements are responsible for high aptamer stability and affinity as well as the 1.5-fold increase in the complex brightness due to the coplanarity of the two heterocycles of the dye. The introduction of mutations into the binding pocket increased the complex brightness and the discriminating ability of the aptamer relative to TO3-biotin, a TO1-biotin analog with the extended conjugated system. In contrast, Mango III in complex with TO1-biotin had a completely different and unusual structure (30). The aptamer possesses molecular connectivity between a two-tetrad G4 and an embedded non-canonical duplex analogous to a pseudoknot. The unique fold of Mango III sandwiched cyanine fragment of TO1-biotin between one of the G4 tetrads and the A10•U17 tertiary base pair provided, together with a structure-guided mutant Mango III(A10U) and a functionally reselected mutant iMango III, the brightest representatives of the Mango family. The crystal structure of the Mango IV aptamer was also studied (28). First, this aptamer forms an unexpected domain-swapping homodimer making it helpful in constructing a FRET system by replacing one protomer with an orthogonal aptamer and changing the linking RNA helix. Second, the aptamer has a «loose» open fluorophore binding pocket that decreases selectivity. At the same time, the propensity of its core to adopt alternative fluorescently active conformations and refold in the cellular media makes it less susceptible to RNA context and tagging to the RNA of interest.

In this paper, we report new Mango-specific fluorogenic dyes and the label length optimization. In dyes design, we focused on the following three problems: (i) simplification of the fluorogenic dye synthesis; (ii) enhancement of the fluorescence quantum yield in the bound state and (iii) adjustment of the emission range. The binding of dyes to two well-characterized Mango aptamers, namely Mango II and Mango IV (24), was studied and the effects of structural alterations on the fluorescence quantum yield were elucidated by molecular modeling. Additionally, to adjust the system for sncRNA visualization and tracking, the total length of the RNA imaging tag (F30-Mango II: 88 nt (24)) was shortened by truncating one of the stems of F30 folding scaffold. This cut resulted in the shortest terminal label for live-cell sncRNA imaging known to date (ds_Mango II: 52 nt). Finally, the applicability of the optimized tag and the new dye with improved characteristics for intracellular imaging and tracking of regulatory sncRNA was verified using an intracellular internalization of *Mycobacterium smegmatis* by a host macrophage.

## MATERIALS AND METHODS

For a detailed description of the synthesis of fluorogenic dyes, see Supporting Information.

### Oligonucleotide synthesis

Oligonucleotides (ONs) (purity > 95%, HPLC) were obtained from Litekh (Russia).

### Absorption spectroscopy, circular dichroism spectroscopy, fluorimetry and binding assays

The secondary structures of Mango-RNA were confirmed by circular dichroism (CD) spectroscopy using 5 μM solutions of free RNAs or their 1:1 complexes with the dyes in the working buffer (10 mM Tris–HCl, pH 7.5, and 140 mM KCl). All samples were heated to 90°C for 5 min and snap-cooled on ice (rapid annealing) or cooled gradually to room temperature (slow annealing) before all measurements. The annealing scheme had no noticeable effect on the CD spectra. The spectra were registered with a Chirascan spectrophotometer (Applied Photophysics, UK) at room temperature in quartz cuvettes of 1 cm path. In all other experiments, rapid annealing was used.

Spectral properties of the new dyes and their 1:1 complexes with Mango RNA or 1:1 mixtures with control RNA/DNA, as well as the 1:4 DFHBI complex with Broccoli RNA, were characterized using 5 μM dye solutions in the same working buffer (10 mM Tris–HCl, pH 7.5, and 140 mM KCl). To register the fluorescence emission of free dyes, 50 μM dye solutions in the working buffer were analyzed, and the intensity was normalized to 5 μM concentration for comparison with the dye–RNA complexes. Complexes of **4b** with genetically engineered RNA constructs were characterized using 1 μM solutions in a 20 mM sodium phosphate buffer, pH 7.2, supplemented with 0.05% Tween-20 and 140 mM КСl. Absorption, fluorescence excitation, and emission spectra were registered with a Chirascan spectrophotometer equipped with the CS/SEM fluorescence accessory at room temperature. Fluorescence quantum yields Ф*^F^* were calculated using equation ((1))


(1) $$\begin{equation*}{\Phi}^F = \Phi_r^F\ \times \frac{{1 - {{10}}^{{A}_r}}}{{1 - {{10}}^A}} \times \frac{F}{{{F}_r}} \times \frac{{{n}^2}}{{n_r^2}}\end{equation*}$$

where Ф*^F^*_r_ = 0.95 is fluorescence quantum yield of the reference dye rhodamine 6G (R6G) in ethanol, *A*_r_ and *F*_r_ are absorbance and fluorescence of R6G in ethanol, *A* and *F* are absorbance and fluorescence of the dye–RNA complex in the working buffer, and *n*_r_ and n are refractive indices of ethanol and the buffer, respectively.

For rough evaluation of **4b**-Mango binding constants, 10 nM solution of **4b** in the working buffer supplemented with 0.05% Tween-20 was titrated with increasing concentrations of Mango RNA, and integral fluorescence was registered in microcapillaries using Monolith NT.115 device (NanoTemper, Germany) equipped with a RED/GREEN detector in RED mode at 25 ºC. Normalized fluorescence was analyzed using MO. Affinity Analysis software (NanoTemper, Germany), and *K*_D_ values were obtained by fitting the experimental data to equation ((2)).


(2) $$\begin{eqnarray*} \frac{{\Delta F}}{{\Delta {F}_{{\rm max}}}} &=& {\rm{\ }}\frac{{\left[ {{\rm dye}} \right] + \left[ {{\rm RNA}} \right] + {K}_{\rm D} - \sqrt {{{\left( {\left[ {{\rm dye}} \right] + \left[ {{\rm RNA}} \right] + {K}_{\rm D}} \right)}}^2 - 4 \cdot \left[ {{\rm dye}} \right] \cdot \left[ {{\rm RNA}} \right])} }}{{2 \cdot \left[ {{\rm dye}} \right]}}\nonumber\\ \end{eqnarray*}$$

where [dye] and [RNA] are total concentrations of **4b** and Mango-RNA, respectively.

### Cell viability assay on murine macrophages (cell culture RAW 264.7, ATCC® TIB-71™)

Two-fold dilutions of studied compounds TO1-biotin and **4a–d** and DMSO as negative control were prepared in cultural medium (FSBSI ‘Chumakov FSC R&D IBP RAS’, Russia). Cell suspensions were added to the wells with compounds dilutions or DMSO control (∼2 × 10^4^ cells per well). The final concentration series of eight dilutions started from 50 μM. The cells were incubated at 37°C in a CO_2_-incubator for 24 h and 7 days. After incubation, the cells were analyzed using Olympus CKX-31 microscope for cell viability. For this, cultural medium was substituted with resazurin solution (25 mg/mL) and cells were incubated at 37°C in a CO_2_-incubator for 4 h. Then 20 ml of 10% SDS was added to stop the reaction and fluorescence was measured with Promega GloMax-Multi Detection System at λ_ex_ 525 nm and λ_em_ 580–640 nm. As additional controls, the same series of cells treated with compounds or DMSO dilutions but without resazurin solution to subtract the background fluorescence were used; and a medium with resazurin solution was used to set up a minimal value of non-reduced resazurin. All experimental procedures were performed in two replicates. Statistical analysis was performed, and fluorescence curves were plotted using MS Excel 2013. The 50% cytotoxic concentration (CC_50_) was calculated (compound concentration required to induce a cytopathic effect in 50% of the cells in a monolayer).

### Molecular modeling

All 3D models were built using the molecular graphics software package Sybyl-X software (Certara, USA). Partial charges on the dyes atoms were calculated using DFT/M06-2X (31)/6–311+g(d,p) and conductor-like polarizable continuum model (CPCM) (32), 6–31g* basis sets and Merz Singh Kollman scheme (33) with RESP (Restrained ElectroStatic Potential) method (34). All quantum mechanics simulations were carried out using Gaussian 09 program (35). To obtain the starting conformations of RNA-dye complexes, docking procedure was performed using ICM-Pro 3.9.2 (36). MD simulations were performed using Amber 20 software (37). The influence of the solvent was simulated using OPC3 (38) model of water molecules. The simulation was performed with periodical boundary conditions and a rectangular box. The buffer between the RNA-dye complex and the periodic box wall was at least 15 Å. For neutralizing the negative charge of RNA backbone, K^+^ ions were used. The parameters needed for interatomic energy calculation were taken from the force fields RNA.YIL (39,40) for RNA and from the general amber force field (gaff2) for the dyes. The MD simulations in the production phase were carried out using constant temperature (*T* = 300 K) and constant pressure (*p* = 1 atm) over 80 ns. To control the temperature, Langevin thermostat was used with a collision frequency of 1 ps^−1^. Energies were estimated by using the MM-GBSA approach. The polar contribution EGB was computed using the Generalized Born (GB) method and the algorithm developed by Onufriev et al. for calculating the effective Born radii (41). The non-polar contribution to the solvation energy (Esurf), which includes solute-solvent van der Waals interactions and the free energy of cavity formation in the solvent, was estimated from a solvent-accessible surface area (SASA).

### Cytotoxicity assays for *M. smegmatis*

The cytotoxic effects of **4b** on bacteria were assessed by MSmeg cellular growth in the presence of the dye at various concentrations since the measurements of bacterial growth rate reflect the concentration of actively growing cells (42). Рre-cultured MSmeg strains bearing empty pAMYC vector (43) were diluted in ratio 1:100 with fresh LB medium containing chloramphenicol (34 μg/ml), Tween 80 (0.05%) and **4b** at a concentration of 400 nM, 1 μM or without dye (positive control), and grown up at 37°C while shaken at 200 rpm. Then, the optical density of the culture (OD_600_) until the stationary growth phase was measured (Supplementary Figure S12).

### *In vitro* transcription

The modular RNA ds_Mango II_MTS1338, ds_Mango II tag and Broccoli aptamer were synthesized using T7 RiboMAX™ Express Large Scale RNA Production System according to the manufacturer's recommendations (Promega, Madison, WI, USA). The DNA templates for RNA synthesis were generated by PCR using the appropriate expression plasmids (see below and (44)) and oligonucleotide primers T7MngII_F/1338_R, T7MngII_F/MngII_R, T7Broc_F/Broc_R (Supplementary Table S2).

### Bacterial strains and growth conditions

*Mycobacterium smegmatis* mc(2)155 (MSmeg) obtained from the bacterial collection of the Bach Institute of Biochemistry (Research Center of Biotechnology of the Russian Academy of Sciences, Moscow, Russia) was pre-cultured for 24 h at 37°C on an orbital shaker (200 rpm) in 40 ml of Luria Bertani (LB) medium supplemented with 0.05% Tween-80 (to prevent cell clumping) and used as inoculums for further experiments. MSmeg recombinant strains were grown in LB supplemented with 34 μg/ml chloramphenicol.

### Construction of MSmeg recombinant strains

The modular RNA and aptamer expression plasmids were constructed by inserting the ds_Mango II_MTS1338 and ds_Mango II encoding DNA fragments synthesized commercially (Evrogen, Russia) into HindIII site of pAMYC (Cm^r^), between mycobacterial rrnB promoter of MSmeg and rrnB-T1 terminator (45). The sequences of the genetic constructs are given in Supplementary Table S2.

Plasmids were amplified in *Escherichia coli* DH5_α grown in Luria Bertani (LB) broth and LB agar supplemented with chloramphenicol (34 μg/mL). MSmeg cells were transformed by electroporation with the constructed plasmids and empty pAMYC vector (negative control), and recombinant strains were selected on LB agar containing with 34 μg/mL chloramphenicol.

### Confocal microscopy of ds_Mango II_MTS1338, ds_Mango II in bacteria and in infected macrophages

Recombinant MSmeg strains were grown up to the logarithmic growth phase (OD_600_ 0.8). Cells were pelleted by centrifugation at 3000 × g for 15 min, washed twice with PBS and either incubated for 30 min in visualization buffer (10 mM Tris pH 7.5, 140 mM KCl, 300 nM Hoechst 33258, 400 nM dye) or resuspended in RPMI-1640 medium (Gibco Europe, Paisley, UK) for infection in macrophages. *M. smegmatis* transformed by pAMYC without insertion was used as a negative control strain.

For the infection RAW 264.7 cells cultured in RPMI-1640 supplemented with 10% fetal calf serum (FCS) (Gibco) were seeded in the same medium on cover glasses (18 × 18 mm Menzel Gläsercoverslips, Thermo Fisher Scientific, Schwerte, Germany) placed in 6-well culture plates (Costar, Cambridge, MA, USA). After 24 h, cells (5 × 10^4^ cells/glass) were infected with ds_Mango II_MTS1338, ds_Mango II or pAMYC strains at MOI 10:1 for 1 h. After 1 h of infection, the medium was removed, and infected macrophages were washed three times with PBS and incubated for 30 minutes in the visualization buffer (see above). Then the samples were analyzed using an Eclipse TE2000 confocal microscope (Nikon, Tokyo, Japan). ds_Mango II_MTS1338 and ds_Mango II in Mycobacteria were detected in a green channel (ex 488/em 590 nm) for TO1-biotin and in a red channel (ex 543/em 650 nm) for 4b, whereas Hoechst 33258-stained cell nuclei were visualized in a blue channel (ex 408/em 515 nm). Because the fluorescent signal from the modular RNA complexes with the dye was clearly visible after 30 min of incubation in the visualization buffer, this time period was long enough for the dye to diffuse into macrophages and phagocytized bacteria and interact with RNA aptamers. Thus, the response time of the sensor is <30 min. The fluorescent signal from the modular RNA complexes with the dye was detectable for at least 2–3 h, suggesting the RNA tag and the dye are relatively stable in the cellular medium, which makes it possible to observe the processes occurring with the tagged molecules in living cells.

## RESULTS

### Design and synthesis of the dyes

**Figure 1.**
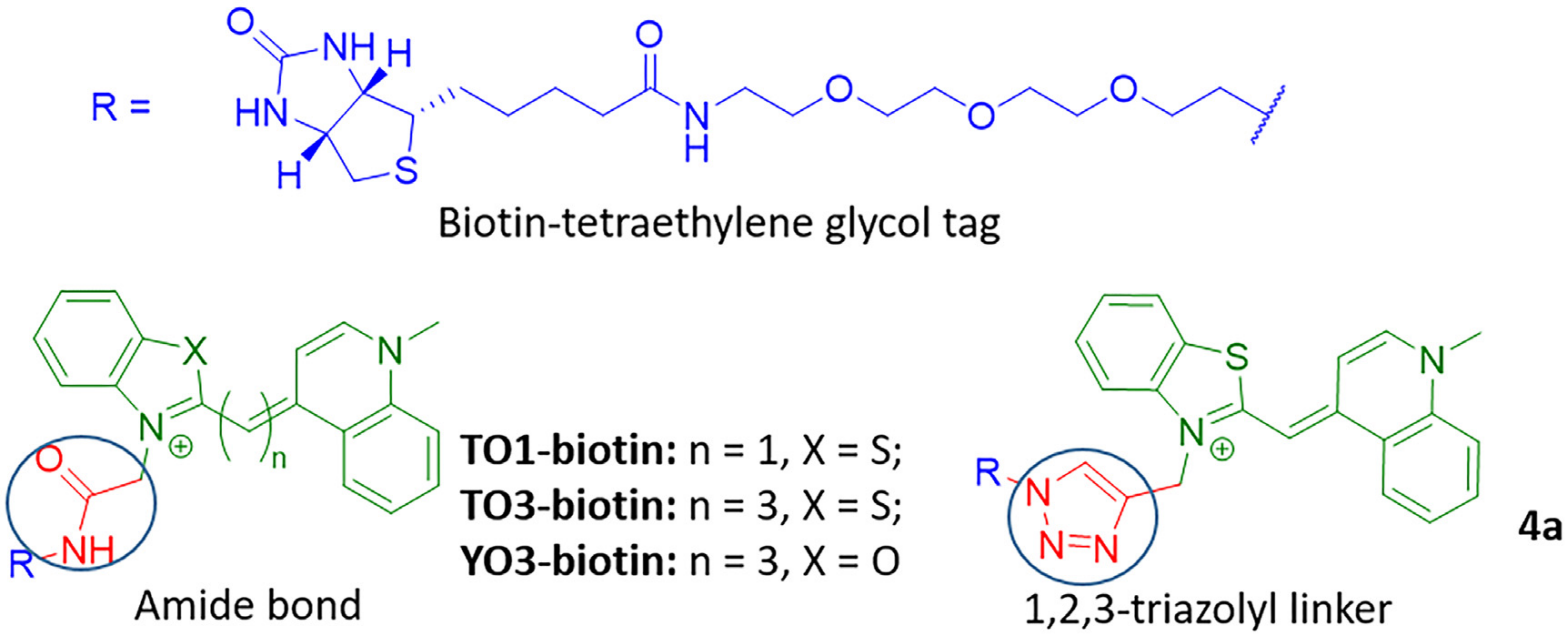
The structures of reported fluorogenic dyes TO1-biotin, TO3-biotin and YO3-biotin and triazolyl-linked TO1-biotin analog **4a**.

The design of new dyes was inspired by the thiazole orange derivatives TO1-biotin, TO3-biotin and YO3-biotin (Figure 1). These derivatives were first synthesized as partners for RNA Mango aptamer (later known as Mango I). TO1-Biotin was successfully used to purify Mango I-tagged transcription-regulating RNA 6S and visualize Mango I-based construct in *C. elegans* (19), and YO3-biotin-Mango I was combined with DFHBI-1T-Spinach pair to obtain a FRET-based system (20). Despite the promise for bioimaging, the application of TO1-biotin, TO3-biotin, and YO3-biotin is limited, partly because synthetic intermediates were obtained in meager 6–12% yields (19,20). The yields for the final acylation reaction are typically not shown, but one can expect some difficulties due to reported HPLC purification.

Nobel prize-recognized ‘click’ chemistry is a synthetic approach for efficient and bioorthogonal conjugating molecules with a high yield (46). The most popular variant of ‘click’ chemistry—1,3-dipolar cycloaddition under copper-(I) catalysis between terminal alkyne function and an azide moiety that leads to the formation of the 1,2,3-triazole cycle (47,48). The replacement of an amide bond with the isosteric 1,2,3-triazolyl might simplify synthetic transformations, increase their efficiency and, in some cases, improve the properties of the biomolecules (49–53).

The structure of TO1-biotin consists of two moieties - a biotin-tetraethylene glycol tag, and a fluorophore, linked by an amide bond (Figure 1). Here, we decided to replace this bond with an isosteric 1,2,3-triazolyl linker in order to increase the efficiency of synthetic transformations and evaluate its influence on the light-up effect upon congener binding with fluorogen-activating aptamers of Mango family.

**Scheme 1.**
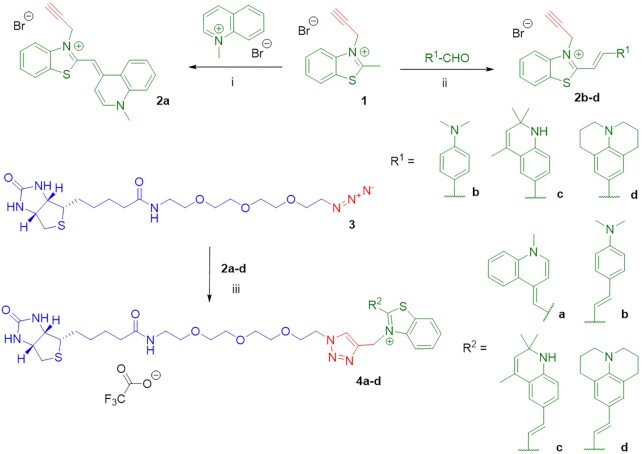
Synthesis of TO1-biotin analogs. Reagents and conditions: (i) TEA, CH_2_Cl_2_; (ii) Ac_2_O, reflux; (iii) CuI, TBTA, DIPEA, CH_3_OH/CH_3_CN, rt, then preparative HPLC.

TO1-biotin was prepared according to the literature (19). For the synthesis of triazolyl-linked analog **4a**, benzothiazolium salt **1** (prepared in a yield of 61%) (54) was condensed with 1-methylquinolinium salt in the presence of TEA to afford alkyne-modified dye **2a** with a yield of 43%, which is ∼7 times higher than the yield for the corresponding intermediate in the TO1-biotin synthesis (19). Following the reported procedure for copper catalyzed azide−alkyne cycloaddition («click» reaction) (55) (Scheme 1, see also Supplementary, chemistry), alkyne-containing derivative **2a** and azido-containing biotinylated derivative **3** (prepared in a yield of 46% for 3 steps, starting from tetraethylene glycole) (56) were condensed in the presence of copper (I) iodide, TBTA and DIPEA affording, after preparative HPLC purification, the target fluorogenic dye **4a** as trifluoroacetate salt. To verify the efficiency of Cu(I)-catalyzed azide–alkyne 1,3-dipolar cycloaddition (CuAAC) reaction compared to the amide bond-forming one, we analyzed crude reaction mixtures containing TO1-biotin and **4a** using LC-MS (Supplementary Figures S1 and S2). The purity assessment at absorption wavelengths of 260 and 500 nm (the latter wavelength is close to the maximum absorption wavelength of TO1-biotin and **4a**) gave 1.2 and 9.2% for TO1-biotin (*R*_f_ = 5.65 min) *versus* 22.4 and 76.5% for **4a** (*R*_f_ = 5.86 min). The reaction yields were 1.8 and 3.5% for TO1-biotin and **4a**, respectively, after a single HPLC purification, presumably due to the low solubility of both derivatives in the loading buffer (water with 0.1% TFA). However, after several iterations of HPLC purification, overall yield of TO1-biotin and **4a** reached 6.8 and 58.1%, respectively (see Supplementary).

Inspired by the chemical design of TO3-biotin and YO3-biotin with extended conjugated systems, which are significantly red-shifted but show drastically reduced fluorescence enhancement upon aptamer binding with respect to the parent TO1-biotin (19,20), and our finding on the utility of simple and effective «click» reaction, we next explored the possibility of using other cyanine derivatives as fluorogenic dyes for Mango-based RNA labeling systems. In the design of the dyes with the red-shifted emission spectra, D–π–A systems, where D and A are an electron donor and an electron acceptor, respectively, find broad application (57). Accordingly, we left the electron-withdrawing benzothiazolium ring (58) in the cyanine push-pull system but replaced 1-methylquinoline ring with strong electron-donating moieties. We also replaced the methine bridge between the donor and acceptor fragments with an ethenyl linker. Thus, we synthesized the dyes containing *N*,*N*-dimethylaminophenyl (**4b**), 2,3,6,7-tetrahydro-1H,5H-pyrido[3,2,1-ij]quinolin-9-yl (**4c**), or 1,2,2,4-tetramethyl-1,2-dihydroquinolin-6-yl (**4d**) group as an electron donor. For this, benzothiazolium salt **1** was condensed with an appropriate aromatic aldehyde via an aldol-type reaction in acetic anhydride (59) to afford the alkyne-modified derivatives **2b-d** (Scheme 1, see Supplementary, chemistry). Their condensation with azido-containing biotinylated derivative **3** in the presence of copper (I) iodide, TBTA and DIPEA yielded, after preparative HPLC purification, fluorogenic dyes **4b–d** as trifluoroacetate salts.

### Spectral properties, affinity, and specificity of the dyes

Spectral properties of the new dyes **4a–d** and the control dye TO1-biotin were investigated in a free state and in complexes with aptamers Mango II and Mango IV (for sequences, see Supplementary Table S1). The former shows enhanced thermal stability compared to Mango I, is resistant to formaldehyde fixation, and has been comprehensively evaluated for bioimaging (24,25,29). The latter is the second actively used aptamer of the Mango family (24,28).

**Figure 2.**
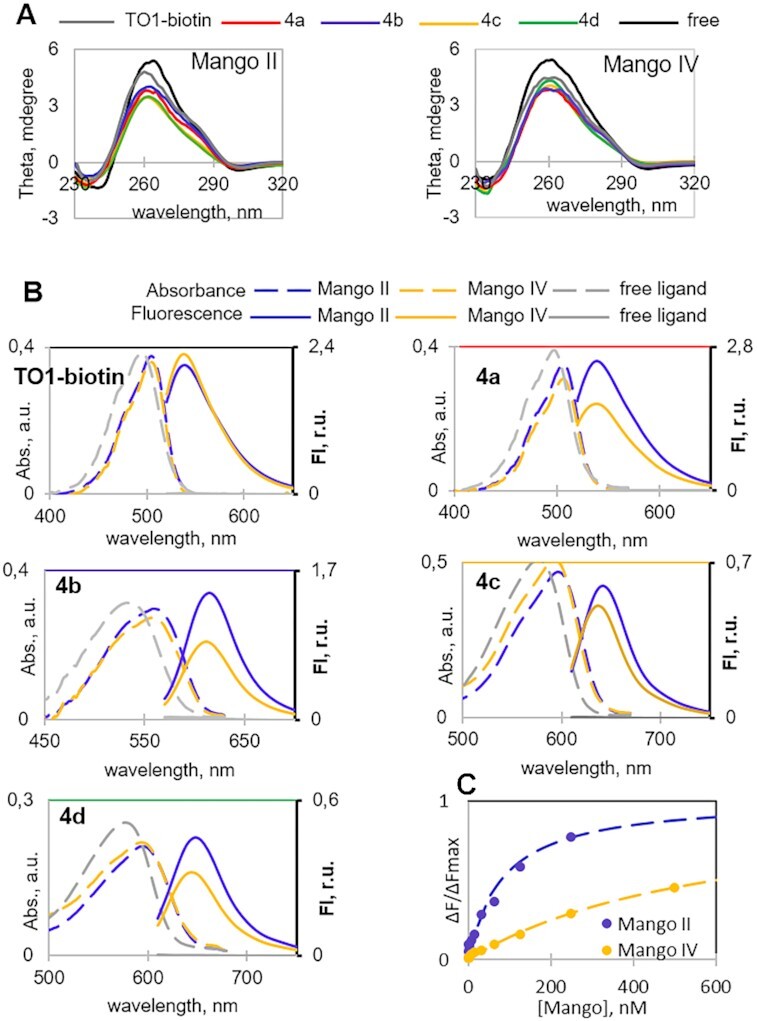
TO1-biotin and new dyes **4a–d** in complexes with Mango II and IV aptamers: spectral properties and binding affinity. (**A**) Verification of dye impact on Mango secondary structure by CD spectroscopy. (**B**) Absorption and fluorescence emission spectra of the free dyes and their complexes. (**C**) **4b**-Mango binding assay. Conditions in A and B: 5 μM Mango aptamer, 5 μM dye, 10 mM Tris–HCl, pH 7.5 and 140 mM КСl. Conditions in C: 10 nM dye.

First, we tested the dyes for disrupting or altering secondary structures of the aptamers. At a concentration of 5 μM, which is well above the reported *K*_D_ values for Mango complexes with TO1-biotin (24), dyes did not induce significant changes in absorbance or circular dichroism (CD) bands of Mango II and Mango IV, except for the minor decrease of CD amplitude (Figure 2A). At the same time, the dyes showed red-shifted absorbance spectra in equimolar mixtures with the aptamers compared to the free state (Figure 2B). Consistent with previous reports for TO1-biotin (24,29), these data support the complex formation and the maintenance of the G4 core in the complexes.

Next, we compared dyes based on their key light-up properties, including the increase of fluorescence and brightness in the bound state. Excitation at complex-specific absorbance maxima (Table 1, Supplementary Figure S3) gave negligible fluorescence emission of the dyes in a free state (*F*_o_), while equimolar mixtures with the aptamers showed up to nearly three orders of magnitude increase in fluorescence emission (*F*_E_ = *F*/*F*_o_, Table 2). While TO1-biotin proved to be an efficient light-up probe for Mango II and Mango IV with fluorescence quantum yields (Ф) equal to 0.39 and 0.43, respectively, its triazole-linked analog **4a** showed preference for Mango II (Ф = 0.42) compared to Mango IV (Ф = 0.32) and a slightly reduced brightness (Table 1). Interestingly, **4a** exhibited higher contrast (*F*_E_) than TO1-biotin, presumably due to the reducing effect of the 1,2,3-triazolyl linker on the rotational flexibility of heteroaromatic systems compared to the amide bond. Dye **4b** was also shown to be a more robust probe for Mango II than Mango IV. Dye **4b** had red-shifted spectra with an increased Stokes shift (Δλ = 55 nm) compared to TO1-biotin and **4a** (Δλ = 35 nm). Dyes **4c** and **4d** were inferior to other variants and showed substantially reduced fluorescence quantum yields and brightness (Table 1).

**Table 1.** Spectral properties of the dyes in complexes with the aptamers

					Φ_Mango-dye (r.u.)^b^		Brightness_Mango-dye (M^−1^cm^−1)b,c^	
Dye	Free dye abs. max (nm)	Free dye^a^ ϵ (M^−1^cm^−1^)	Mango-dye λ_ex_ (nm)	Mango-dye λ_em_ (nm)	Mango II	Mango IV	Mango II	Mango IV
TO1-biotin	495	77500	505	540	0.39	0.43	29200	32200
**4a**	495	77500	505	540	0.42	0.32	29400	19900
**4b**	535	62400	560	615	0.44	0.29	26200	15900
**4с**	580	101100	595	640	0.07	0.05	7100	5400
**4d**	580	51300	595	650	0.14	0.10	6400	4700

^a^Molar extinction at absorbance maximum of the free dye.

^b^Conditions: 5 μM Mango aptamer, 5 μM dye, 10 mM Tris–HCl, pH 7.5, and 140 mM КСl.

^c^Brightness = molar extinction (ϵ) at complex max*Ф.

**Table 2.** Specificity of the light-up effects

	*F*_E_^a^									
Dye	Mango II	Mango IV	Control G4 RNA^b^	dsRNA^b^	ssRNA^b^	dsDNA^b^	ssDNA^b^	h/pG4-DNA^b^	aG4-DNA^b^	*i*-motif^b^
TO1-biotin	2080	2280	1440 (1.6)^c^	132 (16)	84 (25)	24 (87)	12 (173)	54 (39)	48 (43)	42 (50)
**4a**	4770	3200	3310 (1.4)	428 (11)	220 (22)	208 (23)	35 (137)	313 (15)	284 (17)	295 (16)
**4b**	540	320	110 (4.9)	41 (13)	35 (16)	12 (47)	12 (47)	23 (23)	17 (31)	12 (47)
**4с**	320	270	175 (1.8)	23 (1.8)	17 (18)	29 (11)	17 (18)	35 (9)	29 (11)	23 (14)
**4d**	340	240	98 (3.5)	12 (30)	12 (30)	29 (12)	23 (15)	35 (10)	29 (12)	23 (15)

^a^Fluorescence enhancement (*F*_E_) = fluorescence emission in complex (*F*) normalized by fluorescence of the free dye (*F*_o_) at a Mango-specific max. Conditions: 5 μM RNA or DNA, 5 μM dye, 10 mM Tris–HCl, pH 7.5, and 140 mM КСl

^b^ssRNA is a random-sequence RNA (25 μg/mL); for other ON sequences, see Table S1.

^c^Selectivity toward Mango II compared with control RNA or DNA secondary structure as a ratio of *F*/*F*_o_^Mango II^ to *F*/*F*_o_^control^ is shown in parentheses.

To evaluate the binding selectivity of the reported and new dyes towards their RNA aptamers, we compared the light-up effects of dyes in the presence of Mango II, Mango IV, and other DNA/RNA structures using a set of control oligonucleotides (ONs) (Supplementary Table S1). The set included the control G4 RNA *utr-z* from the human KRAS 5'-UTR sequence (60), random-sequence ssRNA from salmon, the RNA hairpin rds26 and the DNA hairpin ds26 (61,62), the telomeric DNA fragment 22AG (63–65), which adopts a parallel-stranded or a hybrid-type G4 structure, along with its mutant 22CTA, which adopts an antiparallel-stranded G4 structure (66), and a DNA fragment from C9ORF72 repeat expansions, which adopts an i-motif structure at near-physiological conditions (67). None of these control ONs was comparable to Mango in terms of the light-up effect (Table 2). Dyes **4a–d** showed somewhat reduced selectivity towards Mango over DNA structures (∼10× Mango:ODN light-up ratio) compared to TO1-biotin (∼100X Mango:ODN light-up ratio). Among the new dyes, the highest selectivity was observed for **4b** (Table 2). Regarding selectivity towards Mango over other RNA structures (especially G4 RNA), **4b** outperformed both **4a** and TO1-biotin (Table 2 and Supplementary Figure S4). For this dye, binding affinity toward Mango aptamers was evaluated in a fluorescence light-up assay, which gave *K*_D_ values of 80 ± 60 nM and 0.6 ± 0.4 μM for Mango II and Mango IV, respectively (Figure 2C).

**Figure 3.**
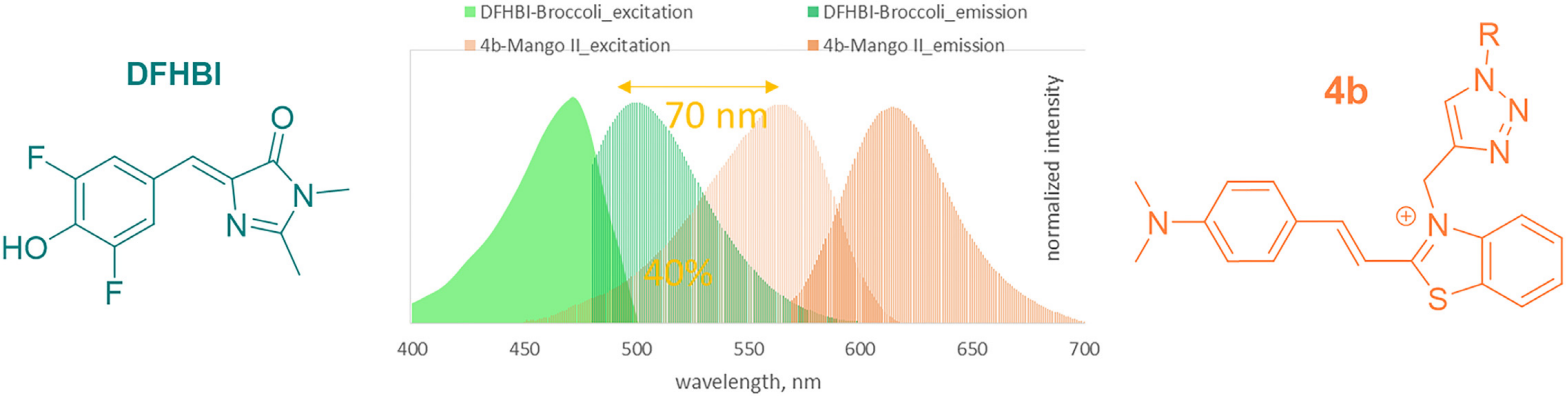
Excitation and emission spectra of DFHBI and **4b** in complex with Broccoli and Mango II aptamers, respectively.

Considering the improved selectivity of **4b** for MangoII/IV over randomly selected G4 RNA and its profound Stokes shift, we questioned whether it could be applied in combination with another dye/aptamer pair for multiplex imaging or FRET-based monitoring of RNA–RNA juxtaposition (20,28). Recently, fluorescent RNA aptamers Spinach and Mango I with their cognate fluorophores were used to develop an RNA apta-FRET system that responded to RNA conformational changes in *E. coli* (20), and DFHBI-1T-Broccoli/YO3-biotin-Mango III donor-acceptor pair was used for performing angle-dependent FRET measurement (26). We evaluated the possibility of using **4b–**Mango II pair as an acceptor paired with DFHBI-Broccoli donor (11) for constructing FRET systems. Broccoli aptamer was synthesized as previously described (44) (for sequence, see Supplementary Table S2). Its CD spectrum (Supplementary Figure S5a) was consistent with a presumed G4 structure. Upon titration with Broccoli aptamer, DFHBI showed a bathochromic absorbance shift (Supplementary Figure S5b) and became fluorescent (Supplementary Figure S5c) when excited at 470 nm (absorbance maximum of the DFHBI-Broccoli complex) with an emission maximum at 510 nm. GFP-derived fluorophores are reportedly insensitive to Mango aptamers (20,28). Thus, the selectivity of **4b** to Mango over Broccoli was the defining question concerning DFHBI-Broccoli and **4b–**Mango II orthogonality. Broccoli-induced absorbance changes (Supplementary Figure S5d) and fluorescence enhancement (Supplementary Figure S5e) of **4b** were negligible compared to those induced by Mango II, indicating no interference between the aptamer-dye pairs. The binding affinity of Broccoli for **4b** was insignificant compared to that of Mango II, according to fluorescence titration assays (Supplementary Figure S5f). At the same time, the pairs showed substantial donor-acceptor spectra overlap (Figure 3), suggesting efficient FRET upon donor-acceptor juxtaposition. To summarize this part, **4b–**Mango II and DFHBI-Broccoli pairs are a perfect match and have prospects in multiplex or FRET-based RNA tracking.

### Molecular modeling

**Figure 4.**
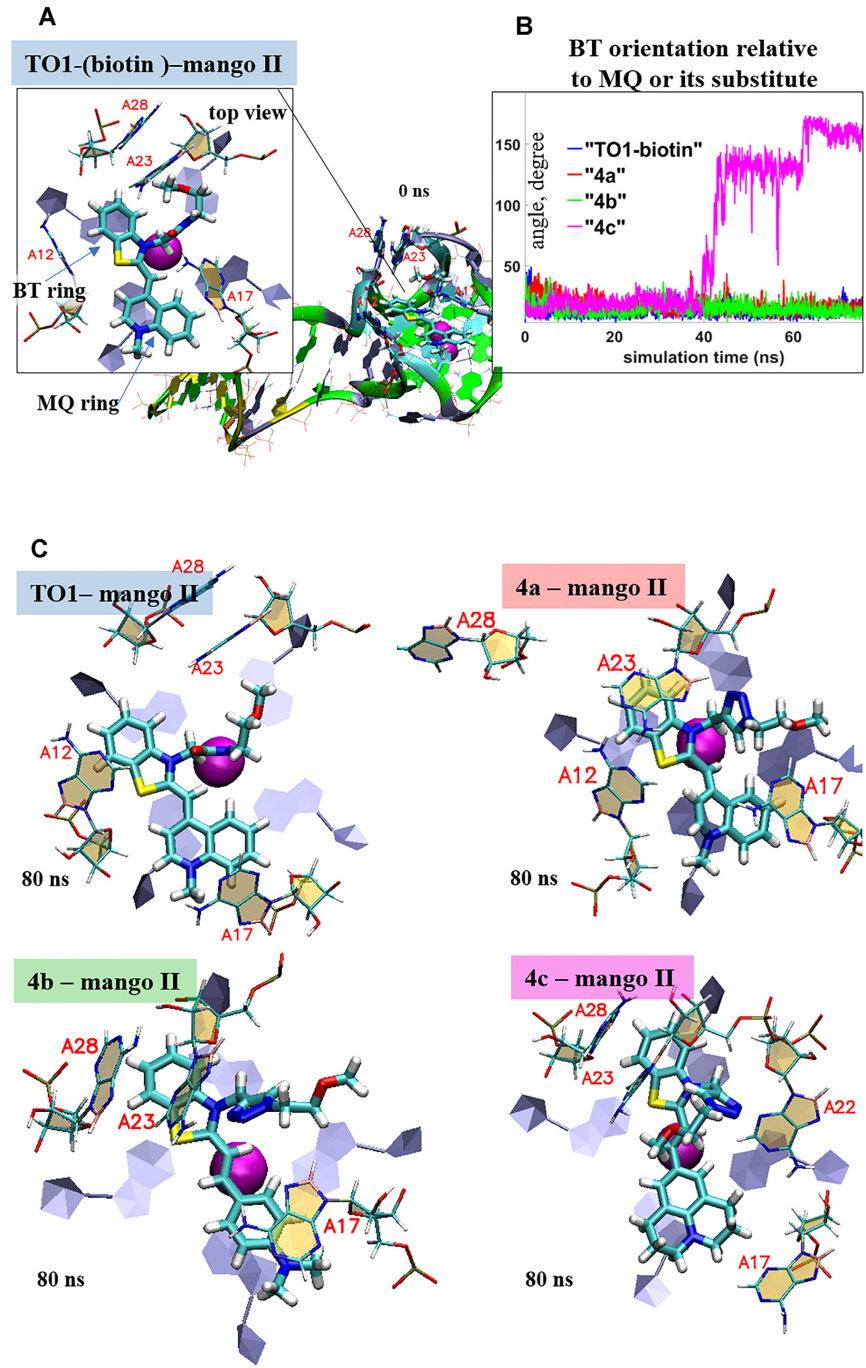
Dynamics of Mango II complexes with TO1-biotin and its analogs: planarity versus rotational mobility of dye residues. (**A**) Initial model of TO1-biotin-Mango II complex (0 ns MD simulation snapshot) illustrating starting positions of benzothiazolium (BT) and methylquinolinyl (MQ) moieties, their stacking with external Mango II G4 tetrad and orientation relative to key neighboring Mango II residues (A12, A17, A23 and A28). Biotin residue is not shown and was excluded from the analysis. (**B**) Evolution of the angle between the planes of BT ring and MQ ring (TO1-biotin and **4a**) or its substitute (**4b–c**). (**C**) End-point conformations of the complexes (80 ns MD simulation snapshots), top views. Color labeling of aptamer residues in TO1-biotin-Mango II full structure (**a**) G, green; A, grey; T, orange; C, cyan. Aptamer residues in complex fragment, top view: C, beige; G, grey. Ligand atoms: C, cyan; N, blue; O, red; S, yellow.

To elucidate the molecular basis for the high light-up effects of **4a** and **4b** (Table 1), we modeled their complexes with Mango II based on the reported structure of TO1-biotin-Mango II (29) (Figure 4A). Dye **4c**, which showed a poor light-up effect, was also analyzed as a semi-negative control. All dye models were truncated slightly by removing the biotin-tetraethylene glycol tag but keeping the amide bond in TO1-biotin and 1,2,3-triazolyl linker in **4a–c**. Amide/1,2,3-triazole linkers of the fluorogenic heteroaromatic system may impact relative positioning of the *N*-methylquinolinyl (MQ) moiety (TO1-biotin and **4a**) or its substitute (**4b** and **4c**) and the benzothiazolium (BT) group in the dye–Mango II complexes and thus could affect fluorescence enhancement. The biotin-containing tag can hardly make a significant contribution, so it was excluded from the analysis. The dyes were localized on the surface of the outed Mango G-quartet by docking, and the stability of the resulting complexes was tested by molecular dynamics (MD) simulations.

The key result of the 80 ns simulation that illustrates the mobility of the fluorogenic heteroaromatic system within the complexes is shown in Figure 4B. While BT and MQ rings in TO1-biotin and **4a**, as well as BT ring and *N,N*-dimethylaminophenyl (DAP) moiety in **4b** remained roughly planar throughout the simulation, in **4c**, the 2,3,6,7-tetrahydro-1H,5H-pyrido[3,2,1-ij]quinolin-9-yl (THPQ) residue underwent full (180°) rotation. End-point structures of the complexes (top views of the dyes and the neighboring aptamer residues) are shown in Figure 4C (for full structures, see Supplementary Figure S6). The observed rotation of THPQ, which caused the energy loss and thus reduced the fluorescent quantum yield of **4c**, likely results from weak stacking interactions between THPQ and A17. In contrast, MQ of TO1-biotin and **4a**, as well as DAP of **4b**, formed stable stacking contacts with A17, as was evident from the plots of COM distances and the angles between the normal and planes of these residues (Supplementary Figure S7).

The 1,2,3-triazole ring did not interfere with the dye geometry but formed additional stacking contacts with the neighboring aptamer residue A23, which led to minor disruption of the local aptamer structure (A23-A26 stacking) in **4a–**Mango II complex. In the **4c–**Mango II complex, the 1,2,3-triazole ring formed additional contacts with A22, and **4c** induced a particularly pronounced alteration of the local aptamer environment, as evidenced by increased RMSD (Supplementary Figure S8). The **4b–**Mango II complex showed minimal fluctuations at the aptamer–dye interface (Supplementary Figure S7), which explains its top performance in fluorescent enhancement assays (Table 1). Despite the lower aptamer binding affinity of **4b** compared to TO1-biotin, it showed the lowest free energy of complex formation (mainly due to van der Waals interactions) among all new dyes (Supplementary Figure S9).

### Application of the new 4b-mango II system for imaging and tracking of the mycobacterial sncRNA

The sncRNAs of intracellular pathogenic bacteria are powerful regulators of bacterial adaptation to the host's immune defense (6,68). In this work, we tested **4b** for intracellular imaging of *M. tuberculosis* small RNA MTS1338, detecting this sncRNA in bacteria *in vitro* and tracking in infected macrophages. For this purpose, we used the modular RNA (ds_Mango II_MTS1338) consisting of MTS1338 fused with ds_Mango II (the optimized fluorescence-activating tag) that was prepared by truncating one of the stems in F30 folding scaffold (Supplementary Figure S10). Since the functional role of the structural elements for the studied RNA has not yet been elucidated, we introduced a tag at the 5'-end of MTS1338 RNA. The sequences of the modular RNA and the genetic constructs used in this work are given in Supplementary Table S2.

The ability of the modular RNA bearing two functional domains, MTS1338 and the fluorogen-activating tag to form a correct secondary structure, according to the RNAfold server (http://rna.-tbi.univie.ac.at/cgi-bin/RNAWebSuite/RNAfold.cgi), was shown (the free energy of the thermodynamic ensemble is –87.79 kcal/mol, Supplementary Figure S10).

To exclude cytotoxicity-related artifacts, the effects of **4b** and other dyes on the metabolic activity of host-cell macrophages and bacterial growth rate were verified. Cytotoxicity of the dyes was evaluated using murine macrophages (RAW 264.7 cell culture, ATCC® TIB-71™). No changes in cell morphology (data not shown) or metabolic activity were observed after 24 h incubation with the dyes (Supplementary Figure S11). At day 7, even at 50 μM concentration, none of the compounds showed a decrease in the level of cell metabolism by more than 50%, suggesting CC_50_ > 50 μM for all tested compounds. However, **4c** and **4d** at this concentration provoked morphological changes in cells (data not shown). We also performed cytotoxicity assays for bacterial cells. For bacteria, no differences were found in growth rate in the presence/absence of **4b** in the medium (Supplementary Figure S12).

Modular RNA and control ds_Mango II were obtained using *in vitro* transcription. The selective light-up effects of **4b** and the control dye TO1-biotin upon their interactions with these RNAs were confirmed *in cuvette*. Consistently with the data in Figure 2, both dyes showed pronounced fluorescence in the presence of the Mango II-tagged RNA but not the tag-free (negative control) RNA (Supplementary Figure S13).

Next, we generated *Mycobacterium smegmatis* (MSmeg) clones expressing the modular RNA, ds_Mango II, and the empty vector-transformed clone (as a negative control). The non-pathogenic MSmeg mc(2)155 was used in the study because its genome and metabolism are very similar to *Mycobacteriumtuberculosis*. MSmeg also has a significantly higher growth rate and much easier undergoes genetic modifications, therefore is widely used as a surrogate organism (69). MSmeg does not contain the MTS1338 gene.

**Figure 5.**
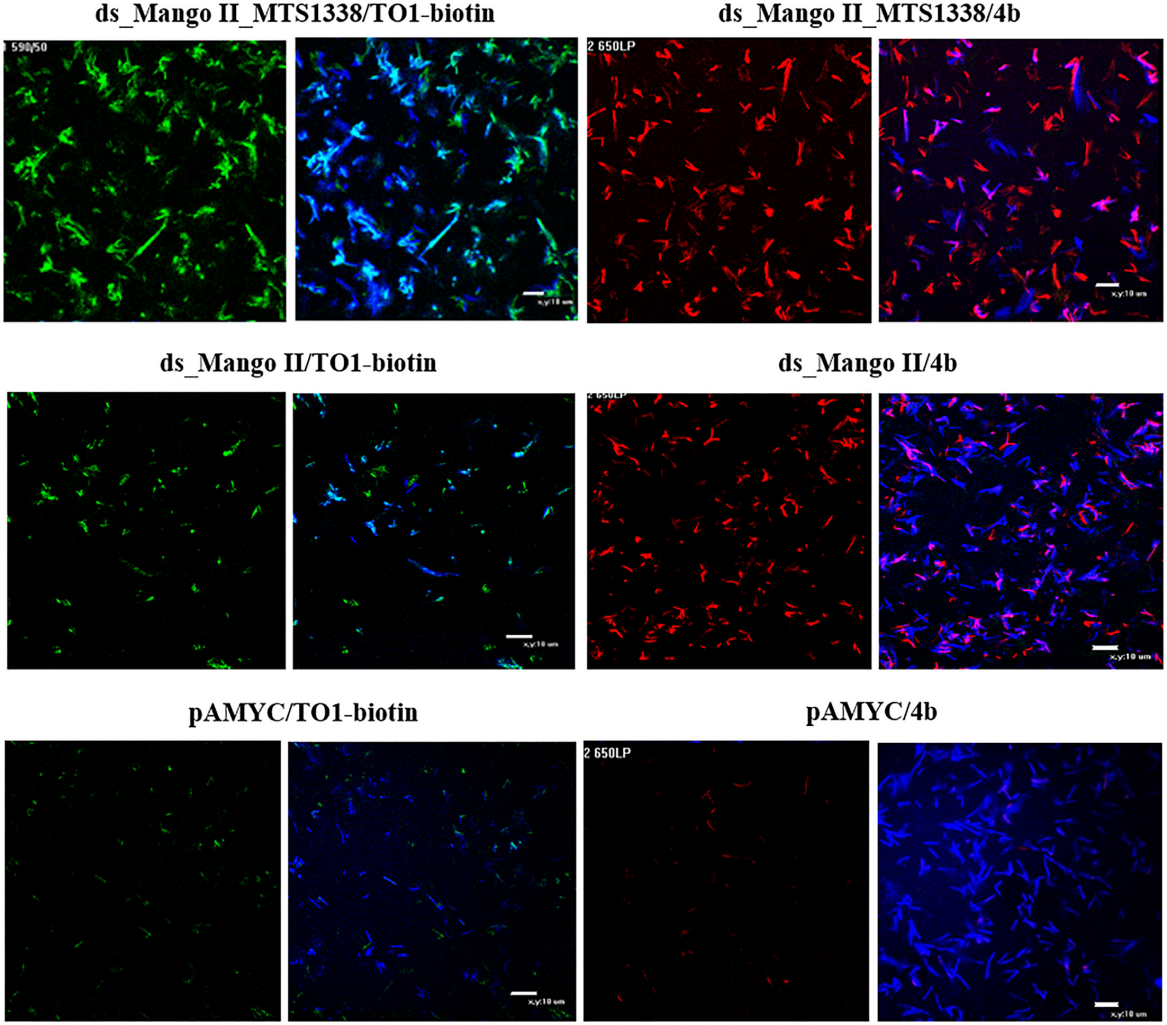
Visualization of the genetically encoded modular RNA ds_Mango II_MTS1338 and ds_Mango II in *M. smegmatis* in solution using TO1-biotin (in a green channel) and **4b** (in a red channel). Bacteria transformed by pAMYC without insertion were used as a negative control. Bacteria were stained with Hoechst 33258 (in a blue channel).

Visualization of the modular RNA and ds_Mango II in live bacterial cells is shown in Figure 5. The images demonstrate high fluorescence of modular RNA and ds_Mango II complexes with both TO1-biotin and **4b**. Since the imaging of live bacteria was performed in solution, the fluorescent signals from the construct/dye complexes (in green/red channels) do not always coincide with those generated by Hoechst 33258 (blue channel) due to the bacteria's movement. No bright green or red signals were detected in experiments with bacteria transformed by empty vector pAMYC and stained with TO1-biotin or **4b**. Control images of bacteria with the empty pAMYC vector (*Msm* pAMYC in buffer, as well as in infected macrophages) are given in Supplementary Figure S14.

When macrophages infected with MSmeg recombinant strains expressing modular RNA or ds_Mango II were stained with TO1-biotin, the fluorescent bacteria were detected, suggesting a correct folding of the label's secondary structure (Supplementary Figure S15).

**Figure 6.**
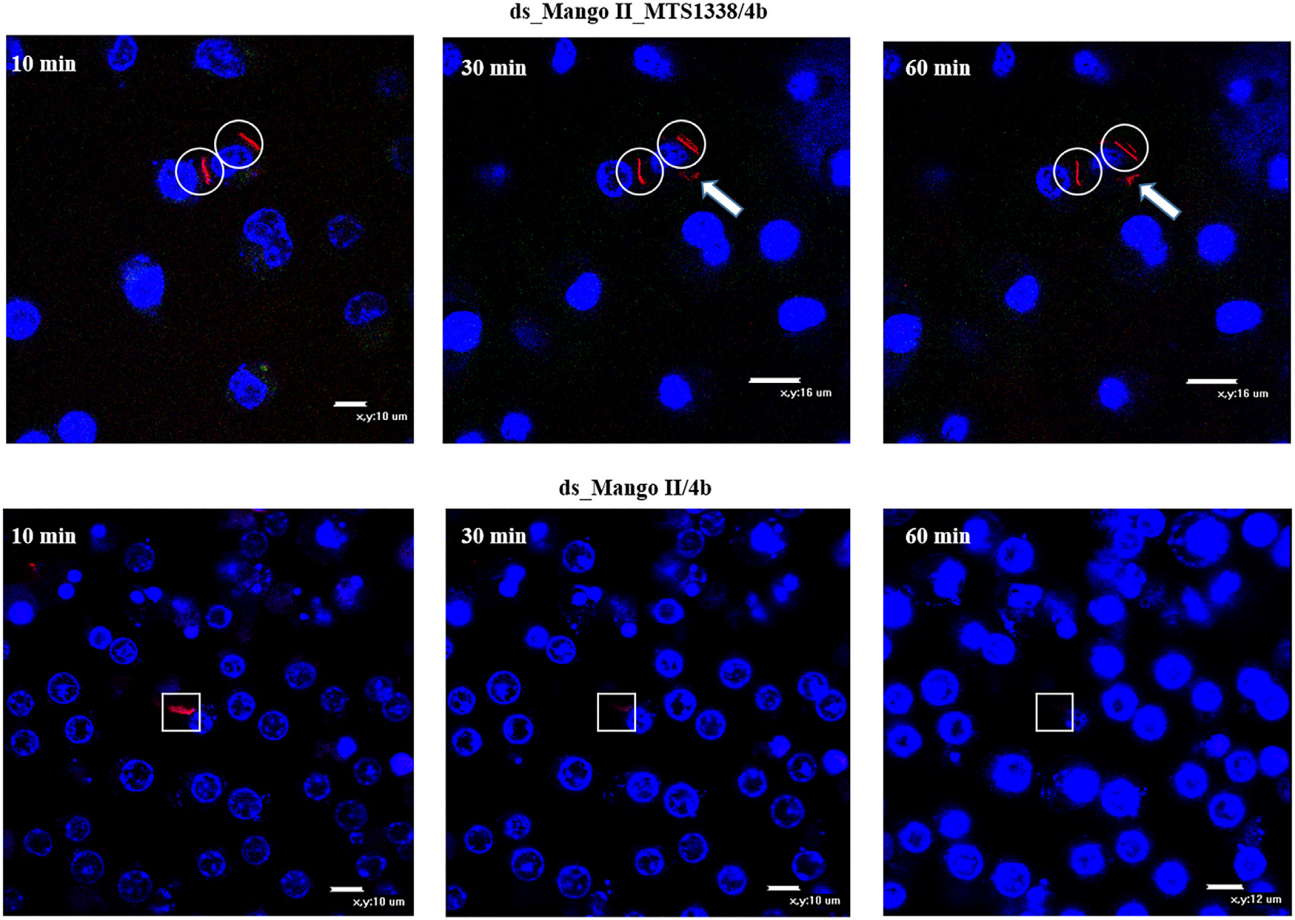
Tracking of the red fluorescent signal for 1 h (10, 30 and 60 min), using the genetically encoded modular RNA or ds_Mango II and **4b** in infected RAW 264.7 macrophages (wide field images). Macrophage nuclei were stained with Hoechst 33258. The circles cover the signal maintenance; white arrows show fluorescent signal movement/occurrence while squares highlight the area of signal disappearance.

**Figure 7.**
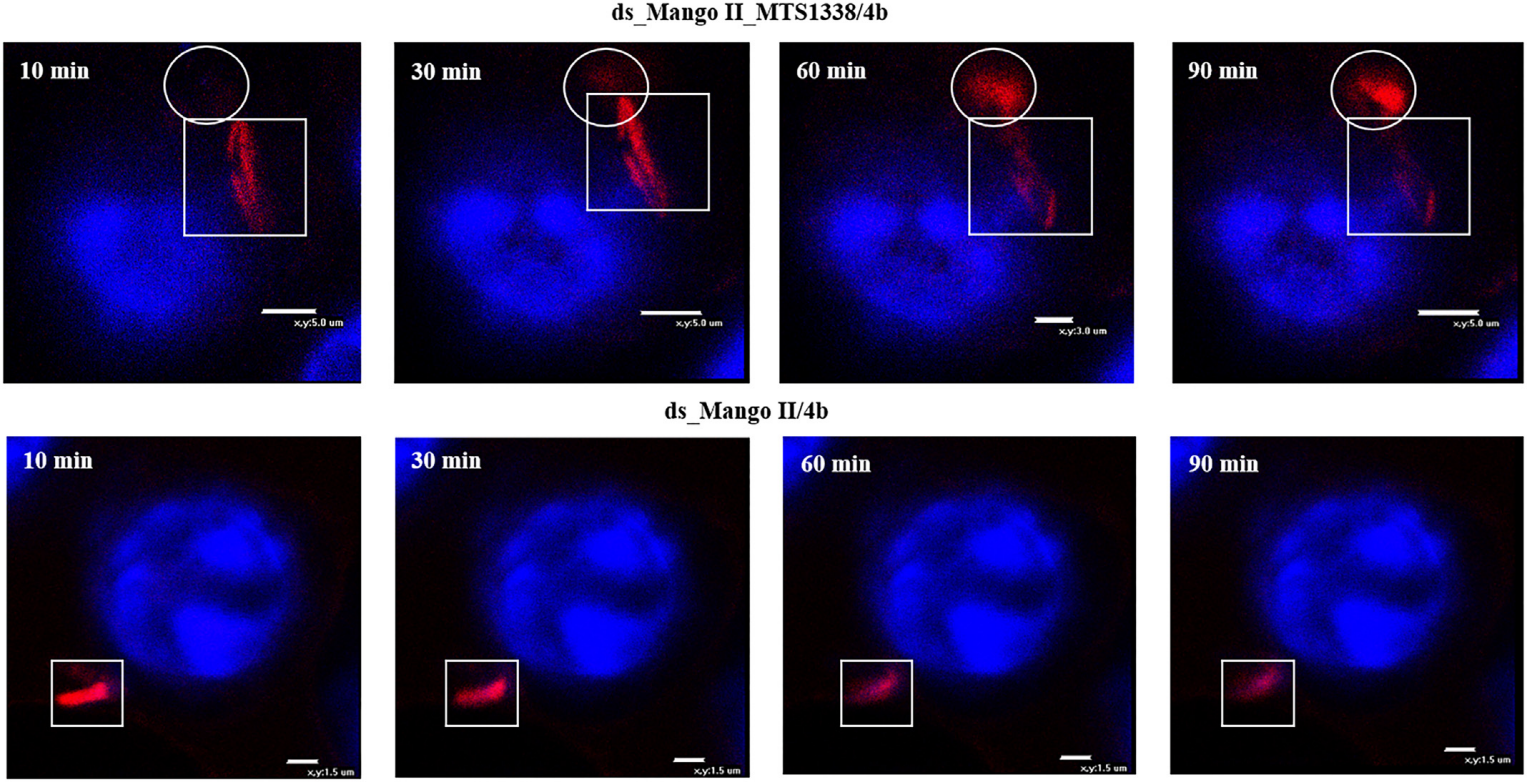
Tracking of the red fluorescent signal for 1.5 h (10, 30, 60 and 90 min), using the genetically encoded modular RNA or ds_Mango II and **4b** in infected RAW 264.7 macrophages (enlarged scale). Macrophage nuclei were stained with Hoechst 33258. The circles show fluorescent signal movement/occurrence, while squares highlight the area of signal disappearance.

To further explore the dissemination and possible secretion of MTS1338 within infected live macrophages, we monitored the changes in the fluorescent signal of modular RNA and ds_Mango II complexes with **4b** during 1 and 1.5 h. For this experiment we chose a field of view to visualize ∼1–2 phagocytosed bacteria (Figures 6 and 7). Since *Mycobacterium smegmatis* is not pathogenic, bacterial cells undergo lysis in phagolysosomes. Also, bacterial cells may be located inside macrophages in different positions relative to the visualization plane. Therefore, after a while, we can visualize mostly fluorescent RNAs, but not bacteria (Supplementary Figure S16). If there were many phagocytosed bacteria in the field of view, fluorescent signals would be difficult to trace and annotate for a concrete bacterium. Thus, a small number of bacteria in the field of view allows us to identify fluorescent signals directly from a particular bacterium and to observe their migration.

Analysis of individual infected cells allowed observing a certain migration of the red fluorescent signal from the modular RNA-expressing bacterial cell inside the infected macrophage, while the outlines of bacteria gradually disappeared. At the same time, no such signal migration was observed in macrophages infected with a control strain expressing ds_Mango II. Instead, we observed gradual attenuation of the signal and the disappearance of the bacterium. Similar to the results obtained by visualizing MTS1338 in infected macrophages with Broccoli aptamer (44), this may indicate the MTS1338 secretion into the cytoplasm of macrophages and is consistent with the presumed function of MTS1338 (70).

Although additional experiments are required to characterize MTS1338 functions further, the data obtained support the role of this sncRNA in bacteria-host interactions. Furthermore, these results confirm successful application of the developed imaging system (short RNA imaging tag + red light-emitting fluorogenic dye) for visualization of sncRNAs within infected living cells.

## DISCUSSION

Fluorescence-activating RNA aptamers can selectively bind to RNA targets, followed by the light up of small molecule fluorogenic dye binders. They are the key player in RNA visualization/tracking systems and FRET constructs that allow to monitor RNA–RNA interactions and their structural rearrangements within the living cell. Among proposed aptamer–dye pairs, the Pepper-based imaging system appears to cover the entire range of visible light emission (16). However, the discriminating ability of Pepper aptamers for dyes structurally dissimilar to their cognate partners has yet to be studied, thus leaving its applicability in FRET studies under question. At the same time, an increase in thermal stability and a decrease in the magnesium-ion dependence of the aptamers are still needed (71). The authors obtained a minimal aptamer of 43 nt in length using the SELEX approach and additional structure optimization. However, the relatively lengthy aptamer D11 (61 nt) was used for tagging natural RNAs of interest, such as 5S ribosomal RNA, 7SK small nuclear RNA and U6 splicing RNA, due to better performance (16).

Mango-based imaging systems also look very promising for both the fluorogenic dye (TO1-biotin) and the aptamers (Mango I–IV and others) (24,29,30). TO1-biotin, stemming from the thiazole orange (TO) core, shows no significant cytotoxicity and has remarkably high photostability, extinction coefficient, quantum yield and brightness (72–75). At the same time, Mango aptamers are notable for their in-cell stability and high binding affinity toward TO1-biotin. Mango aptamers are very short (29–32 nt), albeit an additional F30 folding scaffold (58 nt) is usually required for terminal labeling (24,76). Recently, a tandem Mango II array lacking F30 folding scaffold has been used for imaging single RNA transcripts of β-actin gene in living cells (25); however, such a long tag is hardly suitable for labeling sncRNAs.

Here, an efficient scheme for the synthesis of analogs of TO1-biotin, proposed initially as a fluorogenic dye for Mango aptamers (19), has been developed. Poor yields of the original scheme prompted us to use a simple and efficient CuAAC «click» strategy instead of a common approach via an amide bond-forming reaction (Scheme 1). As a result, **4a**, a TO1-biotin analog with the same thiazole orange (TO) fluorogenic moiety, was prepared in an overall yield of 25% which is ∼60 times higher than in a classical route to TO1-biotin (19). The use of a different synthesis strategy led to the replacement of the amide bond by a 1,2,3-triazolyl linker, thus requiring the study of its effect on the dye spectral properties and the fluorescence-activating ability of Mango aptamers. To evaluate this new dye, the most studied and stable in living cells aptamers Mango II and Mango IV were selected. The new dye **4a** was shown to have spectral properties almost similar to the original dye in relation to Mango II (Table 1). Inspired by these results and the previous synthetic design of YO3-biotin, we replaced the TO fluorogenic fragment with others that could potentially provide red-shifted fluorescence. Among them, **4b** with *N*,*N*-dimethylaminophenyl moiety instead of 1-methylquinolinyl showed properties slightly inferior in brightness to TO1-biotin and **4a**, but at the same time had red-shifted emission maximum at 615 nm (Table 1). The proposed dye in the complex with Mango II has absorption and emission wavelengths similar to complexes of YO3-biotin with MIII AU10 (Mango III mutant) and Peach aptamers (18). Although it is noticeably inferior to them in brightness (26 200 versus 77 300 and 47 600 M^−1^ cm^−1^, respectively), the use of the stable Mango II provides the opportunity for live-cell imaging, in contrast to Mango III and its variants, which have been long studied only *in cuvette*, whereas the applicability of Peach aptamers for intracellular imaging has not yet been evaluated.

Another critical issue is the contrast-providing selectivity of dyes to cognate aptamers over abundant DNA and RNA components within cells, which has not been systematically studied to date. We observed the selectivity of TO1-biotin to Mango aptamers compared to model RNA corresponding to the literature data (19) and a general tendency to increase selectivity when moving from RNA to DNA secondary structures (Table 2). The selectivity of TO1-biotin and **4a** for Mango II compared to genomic RNA G4 *utr-z* from the human KRAS 5'-UTR sequence (60) was only 1.6 and 1.4, respectively. At the same time, **4b** provides an increase in the selectivity to 4.9 (Table 2). The results for TO1-biotin are consistent with those previously obtained for a set of biologically relevant parallel-stranded RNA G4s (77). Despite a significant fluorescence enhancement (*F*_E_) upon TO1-biotin binding to these structures, low affinity (*K*_D_) determines low fluorescent efficiency (*E* = *F*_E_/*K*_D_) of the complexes, which is approximately two orders of magnitude lower than for TO1–biotin–Mango I complex.

Recently, a FRET system was developed based on RNA aptamers Spinach and Mango I that is genetically encodable and responds to conformational changes (20). Mango III and IV were also used to create and study FRET systems *in cuvette* (26,28). Here, we assess the applicability of DFHBI–Broccoli pair that has a brightness comparable to DFHBI-1T-Spinach one, thanks to the higher fluorescence-activating ability of the Broccoli aptamer (11) and **4b–**Mango II in developing FRET system. In terms of the maximum possible FRET efficiency, **4b–**Mango II in combination with DFHBI-Broccoli (40% donor-acceptor overlap upon normalization of all emission/excitation spectra to a maximum intensity of 1) appeared superior to other known FRET pairs, namely DFHBI-Spinach with TO3-biotin-Mango I (23% spectral overlap) and DFHBI-1T-Spinach with YO3-biotin-Mango I (30% overlap) (Figure 3) (20). TO3–biotin–Mango I (acceptor) demonstrated a relatively large (120 nm) excitation redshift relative to the emission of DFHBI-Spinach (donor). Hence there was a modest spectral overlap. Substitution of TO3-biotin for YO3-biotin resulted in a 40 nm excitation blue-shift, which improved the spectral overlap by approximately 30%. Excitation of **4b**-Mango II was blue-shifted further by 20 nm, and its overlap with the donor was improved by an additional 30% compared to YO3–biotin–Mango I. Due to the excitation maximum blue-shift of **4b** and its greater Stokes shift compared to YO3-biotin (Δλ = 55 nm and 40 nm, respectively), the proposed pair is even more suitable for excitation by 488/555 nm lasers and detection using common EGFP and Texas Red filters.

There are examples that noncoding bacterial RNAs of intracellular pathogens can be secreted into the cytoplasm of infected cells and modulate the immune response during infection (78,79). Small *M. tuberculosis* RNA MTS1338 may also be secreted and affects the protective cells of the immune system (80). To study this process, a method for visualizing small bacterial RNAs using fluorescence microscopy is in demand. Previously, we demonstrated the possibility of detecting sncRNA MTS1338 by fluorescence microscopy with the persistence of bacteria in macrophages using F30-2xdBroccoli aptamer (44). However, the aptamer F30-2xdBroccoli is twice as long as the RNA under study (MTS1338-117 nt versus F30-2xdBroccoli-234 nt), which may affect the functional role of the latter. Obviously, the use of another imaging system with a shorter tag and a new dye with higher contrast and selectivity will significantly expand the methodological possibilities for studying the functional properties of the sncRNA in living cells. In this study, we truncated one of the stems of F30 folding scaffold in F30-Mango II tag (88 nt) (24), resulting in the shortest terminal ds_Mango II label (52 nt) for live-cell sncRNA imaging reported to date (Supplementary Figure S10). The optimized tag and the new dye **4b** with improved characteristics for intracellular imaging/tracking regulatory sncRNA were verified using MTS1338. We visualized the modular RNA and ds_Mango II in live bacterial cells using **4b** and TO1-biotin to control the correct aptamer folding (Figure 5). The fluorescence intensity was comparable for both dyes and higher in bacterial cells transfected with the modular RNA-encoding plasmid, probably due to the label-stabilizing ability of MTS1338.

Correct folding of RNA secondary structure in macrophages infected with strains expressing modular RNA or ds_Mango II was confirmed by staining with TO1-biotin (Supplementary Figure S15). Finally, we tracked the red fluorescence signal of **4b** within macrophages infected with bacteria bearing modular RNA or ds_Mango II for 1 and 1.5 h (Figures 6 and 7). In the case of modular RNA, the signal movement was observed, indicating the possible MTS1338 secretion into the cytoplasm of macrophages, while the presence of a tag lacking this RNA was only accompanied by the signal disappearance over time.

In conclusion, a new dye with improved selectivity and red fluorescence has been developed and shown to be promising in combination with stable in living cells Mango II aptamer for RNA imaging/tracking and FRET system development. Furthermore, a shortened fluorescence-activating tag compared to one bearing a widely used F30 stabilizing scaffold for live-cell sncRNA imaging has been proposed, and its effectiveness has been tested for tracking the movement of a fluorescent signal from labeled bacterial RNA in a complex living system consisting of macrophages infected with the *M. tuberculosis* surrogate *M. smegmatis*. Thus, our approach can find applications in developing genetically encodable molecular tools for studying RNA–RNA interactions and conformational rearrangements, as well as sensors and advanced devices.

## Supplementary Material

gkad100_Supplemental_File
