# Supplementary material for: Red light-emitting short Mango-based system enables tracking a mycobacterial small noncoding RNA in infected macrophages
Source: Nucleic Acids Res. 2023 Feb 25;51(6):2586–601. doi: 10.1093/nar/gkad100 (PMC10085697; doi:10.1093/nar/gkad100)
Supplement: gkad100_Supplemental_File [file gkad100_supplemental_file.docx]

**Supporting Information**

**Red light-emitting short Mango-based system enables tracking a mycobacterial small noncoding RNA in infected macrophages**

Oksana S. Bychenko,^1, †^ Alexei A. Khrulev,^1,†^ Julia I. Svetlova,^2,†^ Vladimir B. Tsvetkov,^2,3,†^ Polina N. Kamzeeva,^1^ Yulia V. Skvortsova,^1^ Boris S. Tupertsev,^4^ Igor A. Ivanov,^1^ Leonid V. Aseev,^1^ Yuriy M. Khodarovich,^1^ Evgeny S. Belyaev,^5^ Liubov I. Kozlovskaya,^6,7^ Timofei S. Zatsepin,^8^ Tatyana L. Azhikina,^1,*^ Anna M. Varizhuk,^2,9,*^ and Andrey V. Aralov^1,*^

[Chemistry 2](#_Toc120712636)

[General 2](#_Toc120712637)

[Preparative HPLC purification of TO1-biotin and its analogs 4a-d 2](#_Toc120712638)

[LC-HRMS analysis 3](#_Toc120712639)

[Preparation of intermediates 3](#_Toc120712640)

[General procedure for copper catalyzed azide−alkyne cycloaddition (preparation of final compounds) 4](#_Toc120712641)

[Figures and Tables 10](#_Toc120712642)

[Fig. S1. HPLC profile and purity assessment (Area %) of TO1-biotin 10](#_Toc120712643)

[Fig. S2. HPLC profile and purity assessment (Area %) of 4a 11](#_Toc120712644)

[Table S1. Sequences of chemically synthesized oligonucleotides used for evaluating the spectral properties, affinity and selectivity of fluorogenic dyes 12](#_Toc120712645)

[Fig. S3. Fluorescence excitation spectra of TO1-biotin and new dyes 4a-d in complexes with Mango II 12](#_Toc120712646)

[Fig. S4. Fluorescence emission spectra of TO1-biotin and new ligands 4a-d in complexes with Mango II and control RNA 13](#_Toc120712647)

[Fig. S5. Orthogonal RNA-dye pairs Broccoli-DFHBI and Mango II-4b 13](#_Toc120712648)

[Fig. S6. Molecular dynamics of dye-Mango II complexes: full structures of the complexes at 0 and 80 ns simulation times 14](#_Toc120712649)

[Fig. S7. Molecular dynamics of dye-Mango II complexes: binding modes 15](#_Toc120712650)

[Fig. S8. Molecular dynamics of dye-Mango II complexes: RMSD of the dyes (a) and the aptamer (b) 16](#_Toc120712651)

[Fig. S9. Molecular dynamics simulation: free energy plots 16](#_Toc120712652)

[Fig. S10. The sequences and the secondary structure of F30_Mango II, ds_Mango II aptamers, and modular RNA ds_Mango II_MTS1338 according to the RNAfold server 17](#_Toc120712653)

[Table S2. Nucleotide sequences of the genetic constructs and the oligonucleotides used for their analysis 18](#_Toc120712654)

[Fig. S11. Cytotoxicity of TO1-biotin and 4a-d in RAW 264.7 cell lines after 7 d incubation 19](#_Toc120712655)

[Fig. S12. Evaluation of the dye cytotoxicity for bacterial cells using optical density (OD_600_) measurements 19](#_Toc120712656)

[Fig. S13. Spectra of TO1-biotin and 4b in complexes with the Mango II-labeled genetic construct and control RNA 20](#_Toc120712657)

[Fig S14. The control confocal microscopy images showing 4b staining of Msm_pAMYC in buffer (A) and infected macrophages RAW 264.7 (B). 21](#_Toc120712658)

[Fig S15. Visualization of the genetically encoded modular RNA ds_Mango II_MTS1338 and ds_Mango II tag in *M. smegmatis* in infected RAW 264.7 macrophages using TO1-biotin (in a green channel) 22](#_Toc120712659)

[Fig S16. Visualization of the genetically encoded modular RNA ds_Mango II_MTS1338 using 4b in infected macrophages RAW 264.7 22](#_Toc120712661)

[HPLC and MS data 36](#_Toc120712662)

[References 45](#_Toc120712663)

# Chemistry

## General

All reagents and solvents were commercially available unless otherwise mentioned. Thin layer chromatography (TLC) was performed on plates (Merck) precoated with silica gel (60 μm, F254) and visualized using UV light (254 and 365 nm). Column chromatography (CC) was performed on silica gel (0.040−0.063 mm, Merck, Germany). ^1^H and ^13^C NMR spectra were recorded on the Bruker Avance III 600 spectrometer at 600 and 151 MHz, respectively. Chemical shifts are reported in δ (ppm) units using residual 1H signals from deuterated solvents as references. The multiplicity are reported using the following abbreviations: s (singlet), d (doublet), t (triplet), m (multiplet) and br (broad). The coupling constants (J) are given in Hz. ESI HR mass spectra were acquired on a Thermo Scientific LTQ Orbitrap hybrid instrument (Thermo Electron Corp., Bremen, Germany) in continuous flow direct sample infusion (positive ion mode). 2-Methyl-3-(prop-2-yn-1-yl)benzo[d]thiazol-3-ium bromide **1** was prepared according to the reported method^1^.

## Preparative HPLC purification of TO1-biotin and its analogs 4a-d

Purification of the compounds was performed on a Gilson HPLC system (331/332 pump with GX-271 liquid handler) using an Luna C18(2) (100×21.20 mm, 5 μm) column. MeCN (with 0.1% TFA) and aq. TFA (0.1%) were used as eluents. UV detection was achieved at 210 nm and 280 nm. TO1-biotin and **4a-d** were purified in a linear gradient from 20 to 70% of MeCN in 45 min at a flow rate of 25 mL/min. Fractions containing the target compound were collected, organic solvent was removed *in vacuo* and water was lyophilized.

## LC-HRMS analysis

All experiments were carried out on a Dionex 3000 UltiMate HPLC system coupled with QExactive Orbitrap mass spectrometer (Bremen, Germany) using Macherey-Nagel Pyramid С18 Nucleodur (2.0×100 mm, 1.8 µm) HPLC column. Mobile phase A: 0.1% formic acid in 5% aqueous solution of acetonitrile; mobile phase B: 0.1% formic acid in acetonitrile. The following gradient at a flow rate of 0.50 mL/min was used: 0-1.0 min 5% B, 1.0-10.0 min 5-95.0% B, 10.0-12.0 95% B, 12.0-13.0 min 95.0-5% B, 13.0-15.0 min 5% B. The injection volume was 1 μL. The resolving power was 35 000 (for m/z = 200). The Sheath, Aux and Spare gases were set to 45, 35 and 5, respectively. The Spray Voltage was 4.1 kV, the temperature of the desolvating capillary was 350 °C. S-Leans RF level was 50 and the source temperature was set to 200 °C. All mass spectra were obtained in Full MS followed by data dependent analysis (DDa) in a positive ionization mode. The retention time are shown in the relevant parts of the chemical procedures related to product characterization.

## Preparation of intermediates

*(E)-2-((1-methylquinolin-4(1H)-ylidene)methyl)-3-(prop-2-yn-1-yl)benzo[d]thiazol-3-ium bromide* **2a**

To a suspension of 2-methyl-3-(prop-2-yn-1-yl)benzo[d]thiazol-3-ium bromide **1** (270 mg, 1 mmol) in CH_2_Cl_2_ (20 mL) 1-methylquinolinium bromide (270 mg, 1.2 mmol) and TEA (0.7 mL, 5 mmol) were added and the resulting mixture was stirred at rt for 48 hours. The mixture was concentrated *in vacuo*. The solid obtained was triturated with Et_2_O (3 x 15 mL) and then purified by column chromatography on silica gel (1→10% CH_3_OH in CH_2_Cl_2_) yielding **2a** (176 mg, 0.43 mmol, 43 %) as a red solid. ^1^H NMR (600 MHz, DMSO-d6): δ 8.81 (d, J = 8.5 Hz, 1H), 8.78 (d, J = 7.1 Hz, 1H), 8.13 (d, J = 8.7 Hz, 1H), 8.06 (d, J = 7.1 Hz, 1H), 8.04 (d, J = 8.2 Hz, 1H), 7.85 (t, J = 7.7 Hz, 1H), 7.79 (d, J = 8.3 Hz, 1H), 7.61 (t, J = 7.8 Hz, 1H), 7.46 (d, J = 7.1 Hz, 1H), 7.41 (t, J = 7.6 Hz, 1H), 7.05 (s, 1H), 5.59 (d, J = 1.9 Hz, 2H), 4.24 (s, 3H), 3.53 (t, J = 1.9 Hz, 1H). ^13^C NMR (151 MHz, DMSO-d6): δ 158.18, 149.00, 145.54, 139.12, 137.96, 133.32, 128.08, 127.18, 125.29, 124.44, 124.19, 123.36, 122.93, 118.40, 112.44, 108.65, 88.02, 76.82, 76.31, 42.55, 35.32. HRMS (ESI) m/z: calcd for C_21_H_17_N_2_S^+^ [M-Br]^+^: 329.1107; found 329.1118; t_R_ = 4.01 min.

*(E)-2-(2-(4-(Dimethylamino)phenyl)ethenyl)-3-(2-propynyl)benzo[d]thiazolium bromide* **2b**

This derivative (yield 79%) was prepared according to the reported method^2^. ^1^H NMR (600 MHz, DMSO-*d_6_*): δ 8.32 (d, J = 8.0 Hz, 1H), 8.14 (d, J =15.0 Hz, 1H), 8.13 (d, J =8.3 Hz, 1H), 7.95 (d, J =8.7 Hz, 2H), 7.80 (t, J =7.7 Hz, 1H), 7.74 (d, J =15.0 Hz, 1H), 7.68 (t, J =7.6 Hz, 1H), 6.86 (d, J = 8.7 Hz, 2H), 5.82 (s, 2H), 3.72 (t, J = 2.0 Hz, 1H), 3.13 (s, 6H). ^13^C NMR (151 MHz, DMSO-d6): δ 171.34, 153.83, 151.56, 140.31, 133.30 (2C), 128.92, 127.40, 126.57, 123.99, 121.43, 115.50, 112.01, 105.18, 78.01, 75.96, 37.48. HRMS (ESI) m/z: calcd for C_20_H_19_N_2_S^+^ [M-Br]^+^: 319.1263; found 319.1255; t_R_ = 4.11 min.

*(E)-3-(prop-2-yn-1-yl)-2-(2-(2,3,6,7-tetrahydro-1H,5H-pyrido[3,2,1-ij]quinolin-9-yl)vinyl)benzo[d]thiazol-3-ium bromide* **2c**

This derivative (yield 74%) was prepared according to the reported method^2^. ^1^H NMR (600 MHz, DMSO-d6): δ 8.22 (d, J = 8.0 Hz, 1H), 8.02 (d, J = 8.5 Hz, 1H), 7.93 (d, J = 14.8 Hz, 1H), 7.75 (t, J = 7.7 Hz, 1H), 7.63 (t, J = 7.8 Hz, 1H), 7.52 (s, 2H), 7.48 (d, J = 7.8 Hz, 1H), 5.66 (d, J = 2.2 Hz, 2H), 3.59 (t, J = 2.2 Hz, 1H), 3.44-3.39 (m, 4H), 2.78-2.74 (m, 4H), 1.96-1.90 (m, 4H). HRMS (ESI) m/z: calcd for C_24_H_23_N_2_S^+^ [M-Br]^+^: 371.1576; found 371.1593; t_R_ = 4.81 min.

*(E)-3-(prop-2-yn-1-yl)-2-(2-(1,2,2,4-tetramethyl-1,2-dihydroquinolin-6-yl)vinyl)benzo[d]thiazol-3-ium bromide* **2d**

A mixture of 2-methyl-3-(prop-2-yn-1-yl)benzo[d]thiazol-3-ium bromide **1** (270 mg, 1 mmol) and 1,2,2,4-tetramethyl-1,2-dihydroquinoline-6-carbaldehyde (430 mg, 2 mmol) in acetic anhydride (3 mL) was stirred at 90°C for 8 h. Then, to a mixture H_2_O (0.5 mL) was added and the stirring was continued for 30 min followed by concentration *in vacuo*. The residue was purified by column chromatography on silica gel (1→10% CH_3_OH in CH_2_Cl_2_) yielding **2d** (330 mg, 0.71 mmol, 71 %) as a purple solid. ^1^H NMR (600 MHz, DMSO-d6): δ 8.30 (d, J = 8.0 Hz, 1H), 8.13 (d, J = 15.0 Hz, 1H), 8.11 (d, J = 8.7 Hz, 1H), 7.86 (d, J = 8.9 Hz, 1H), 7.80 (t, J = 7.9 Hz, 1H), 7.68 (t, J = 7.7 Hz, 1H), 7.64 (d, J = 15.0 Hz, 1H), 7.61 (d, J = 1.6 Hz, 1H), 6.72 (d, J = 8.9 Hz, 1H), 5.79 (d, J = 2.2 Hz, 2H), 5.53 (s, 1H), 3.71 (t, J = 2.2 Hz, 1H), 2.99 (s, 3H), 2.04 (s, 3H), 1.40 (s, 6H). ^13^C NMR (151 MHz, DMSO-d6): δ 170.98, 151.54, 149.82, 140.34, 134.34, 130.15, 128.89, 127.32, 126.45, 125.72 (2C), 123.92, 121.51, 121.49, 115.41, 110.74, 104.62, 78.03, 75.99, 58.14, 37.40, 31.48, 28.55, 18.19. HRMS (ESI) m/z: calcd for C_25_H_25_N_2_S^+^ [M-Br]^+^: 385.1733; found 385.1746; t_R_ = 5.08 min.

*N-(2-(2-(2-(2-azidoethoxy)ethoxy)ethoxy)ethyl)-5-(2-oxohexahydro-1H-thieno[3,4-d]imidazol-4-yl)pentanamide* **3**

This derivative (83%) was prepared starting from 2-(2-(2-(2-azidoethoxy)ethoxy)ethoxy)ethan-1-amine according to the reported method^3^. ^1^H NMR (600 MHz, DMSO-d6): δ 7.80 (t, J = 5.5 Hz, 1H), 6.39 (s, 1H), 6.34 (s, 1H), 4.33-4.29 (m, 1H), 4.15-4.11 (m, 1H), 3.62-3.59 (m, 2H), 3.58-3.49 (m, 8H), 3.42-3.38 (m, 4H), 3.21-3.17 (m, 2H), 3.12-3.08 (m, 1H), 2.83 (dd, J = 12.4 Hz, J = 5.1 Hz, 1H), 2.59 (d, J = 12.4 Hz, 1H), 2.07 (t, J = 7.4 Hz, 2H), 1.65-1.58 (m, 1H), 1.55-1.44 (m, 3H), 1.36-1.24 (m, 2H). ^13^C NMR (151 MHz, DMSO-d6): δ 172.1, 162.6, 69.7, 69.7, 69.6, 69.4, 69.1, 69.0, 61.0, 59.1, 55.3, 49.9, 39.7 (overlaps with DMSO), 38.4, 35.0, 28.1, 27.9, 25.1.

TO1-biotin (yield 6.8%) was prepared according to the reported condensation method^4^. HRMS (ESI) m/z: calcd for C_38_H_49_N_6_O_6_S_2_^+^ [M-CF_3_COO^-^]^+^: 749.3150; found 749.3143; t_R_ = 7.42 min.

## General procedure for copper catalyzed azide−alkyne cycloaddition (preparation of final compounds)

A solution of the corresponding alkyne-containing derivative **2** (0.06 mmol) and azido-containing biotinylated derivative **3** (40 mg, 0.09 mmol, 1.5 eq) in a mixture of CH_3_OH/CH_3_CN (1:1, v/v, 5 mL) was degassed and then copper (I) iodide (1.1 mg, 0.006 mmol, 0.1 eq), TBTA (1.6 mg, 0.003 mmol, 0.05 eq) and DIPEA (55 μL, 0.3 mmol, 5.0 eq) were sequentially added under a nitrogen atmosphere. The reaction mixture was stirred at room temperature overnight and concentrated *in vacuo*. The residue was partitioned between a mixture of *n*-butanol/CH_2_Cl_2_ (1:10, v/v, 10 mL) and 0.1 M EDTA solution (10 mL) and the aqueous phase was additionally extracted with *n*-butanol/CH_2_Cl_2_ (1:10, v/v, 10 mL). The combined organic layers was concentrated *in vacuo* and the residue obtained was subjected to preparative HPLC.

*2-((-1-methylquinolin-4(1H)-ylidene)methyl)-3-((1-(13-oxo-17-((3aR,4R,6aS)-2-oxohexahydro-1H-thieno[3,4-d]imidazol-4-yl)-3,6,9-trioxa-12-azaheptadecyl)-1H-1,2,3-triazol-4-yl)methyl)benzo[d]thiazol-3-ium 2,2,2-trifluoroacetate* **4a**


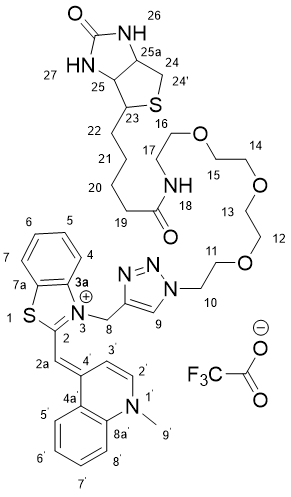


(Yield 58.1 %). ^1^H NMR (600 MHz,DMSO-*d*_6_): δ 8.78 (1H, d, *J* = 8.6 Hz, **Н5**'), 8.67 (1H, d, *J* = 7.3 Hz, **Н2**'), 8.31 (1H, s, **Н9**), 8.13 – 8.10 (1H, m, **Н8**'), 8.07 – 8.03 (2H, m, **Н7**', **Н7**), 7.96 (1H, d, *J* = 8.3 Hz, **Н4**), 7.86 – 7.82 (1H, m, **Н6**'), 7.80 – 7.75 (1H, m, **Н18**), 7.65 – 7.61 (1H, m, **Н5**), 7.45 – 7.40 (2H, m, **Н3**', **Н6**), 7.35 (1H, s, **Н2а**), 6.38 (1H, s, **Н26**), 6.34 (1H, s, **Н27**), 5.93 (2H, s, **Н8**), 4.51 (2H, t, *J* = 5.1 Hz, **Н10**), 4.32 – 4.27 (1H, m, **Н25а**), 4.22 (3H, s, **Н9**'), 4.13 – 4.09 (1H, m, **Н25**), 3.77 (2H, t, *J* = 5.1 Hz, **Н11**), 3.45 – 3.37 (10H, m, **Н12-Н16**), 3.19 – 3.13 (2H, m, **Н17**), 3.10 – 3.05 (1H, m, **Н23**), 2.81 (1H, dd, *J* = 12.4, 5.1 Hz, **Н24**), 2.57 (1H, d, *J* = 12.4 Hz, **Н24**'), 2.05 (2H, t, *J* = 7.5 Hz, **Н19**), 1.54 – 1.39 (6H, m, **Н20-Н22**). HRMS (ESI) m/z: calcd for C_39_H_49_N_8_O_5_S_2_^+^ [M-CF_3_COO^-^]^+^: 773.3262; found 773.3260; t_R_ = 7.91 min.

*2-(4-(dimethylamino)styryl)-3-((1-(13-oxo-17-((3aR,4R,6aS)-2-oxohexahydro-1H-thieno[3,4-d]imidazol-4-yl)-3,6,9-trioxa-12-azaheptadecyl)-1H-1,2,3-triazol-4-yl)methyl)benzo[d]thiazol-3-ium 2,2,2-trifluoroacetate* **4b**


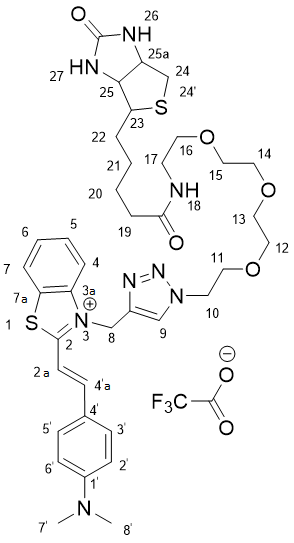


(Yield 37.4 %). ^1^H NMR (600 MHz, DMSO-*d*_6_): δ 8,34 (1H, s, **Н9**), 8.29 (1H, d, *J* = 8.0 Hz, **Н7**), 8.24 (1H, d, *J* = 8.9 Hz, **Н4**), 8.16 (1H, d, *J* = 14.6 Hz, **Н2а**), 7.93 (2H, d, *J* = 9.0 Hz, **Н2**', **Н6**'), 7.89 (1H, d, *J* = 15.2 Hz, **Н4**'**а**), 7.80 – 7.75 (2H, m, **Н5**, **Н18**), 7.69 – 7.65 (1H, m, **Н6**), 6.89 (2H, d, *J* = 9.0 Hz, **Н3**', **Н5**'), 6.38 (1H, s, **Н25**), 6.34 (1H, s, **Н26**), 6.17 (2H, s, **Н8**), 4.52 (2H, t, *J* = 5.1 Hz, **Н10**), 4.32 – 4.28 (1H, m, **Н25а**), 4.14 – 4,10 (1H, m, **Н25**), 3.77 (2H, t, *J* = 5.1 Hz, **Н11**), 3.42 – 3.37 (10H, m, **Н12-Н16**), 3.19 – 3.15 (2H, m, **Н17**), 3.14 (6H, s, **Н7**', **Н8**'), 3.10-3.06 (1H, m, **Н23**), 2.81 (1H, dd, *J* = 12.4, 5.1 Hz, **Н24**), 2.59 – 2.56 (1H, m, **Н24**'), 2.05 (2H, t, *J* = 7.5 Hz, **Н19**), 1.64 – 1.40 (6H, m, **Н20-Н22**). HRMS (ESI) m/z: calcd for C_38_H_51_N_8_O_5_S_2_^+^ [M-CF_3_COO^-^]^+^: 763.3418; found 763.3403; t_R_ = 8.27 min.

*3-((1-(13-oxo-17-((3aR,4R,6aS)-2-oxohexahydro-1H-thieno[3,4-d]imidazol-4-yl)-3,6,9-trioxa-12-azaheptadecyl)-1H-1,2,3-triazol-4-yl)methyl)-2-(2-(2,3,6,7-tetrahydro-1H,5H-pyrido[3,2,1-ij]quinolin-9-yl)vinyl)benzo[d]thiazol-3-ium 2,2,2-trifluoroacetate* **4c**


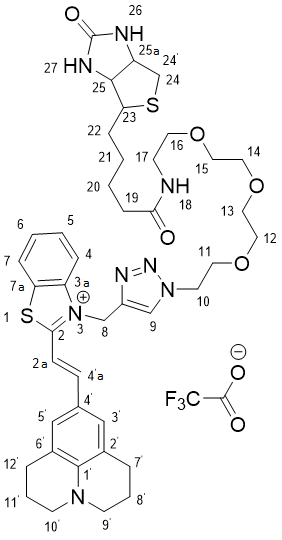


(Yield 40.4 %) ^1^H NMR (600 MHz, DMSO-*d*_6_): δ 8.32 (1H, s, **Н9**), 8.23 (1H, d, *J* = 8.0 Hz, **Н7**), 8.12 (1H, d, *J* = 8.4 Hz, **Н4**), 7.95 (1H, d, *J* = 14.8 Hz, **Н2а**), 7.81 – 7.76 (1H, m, **Н18**) 7.73 – 7.68 (2H, m, **Н5, Н4**'**а**), 7.63 – 7.59 (1H, m, **Н6**), 7.52 (2H, s, **Н3**', **Н5**'), 6.38 (1H, s, **Н26**), 6.34 (1H, s, **Н27**), 6.08 (2H, s, **Н8**), 4.52 (2H, t, *J* = 5.1 Hz, **Н10**), 4.32 – 4.28 (1H, m, **Н25а**), 4.14 – 4.10 (1H, m, **Н25**), 3.78 (2H, t, *J* = 5.1 Hz, **Н11**), 3.46 – 3.35 (14H, m, **Н12-Н16, Н10**', **Н9**'), 3.21 – 3.13 (2H, m, **Н17**), 3.12 – 3.05 (1H, m, **Н23**), 2.81 (1H, dd, *J* = 12.4, 5.1 Hz, **Н24**), 2.74 (4H, t, *J* = 6.3 Hz, **Н7**', **Н12**'), 2.58 (1H, d, *J* = 12.4 Hz, **Н24**'), 2.05 (2H, t, *J* = 7.4 Hz, **Н19**), 1.94 – 1.88 (4H, m, **Н11**', **Н8**'), 1.65 – 1.40 (6H, m, **Н20-Н22**). HRMS (ESI) m/z: calcd for C_42_H_55_N_8_O_5_S_2_^+^ [M-CF_3_COO^-^]^+^: 815.3731; found 815.3768; t_R_ = 4.46 min.

*3-((1-(13-oxo-17-((3aR,4R,6aS)-2-oxohexahydro-1H-thieno[3,4-d]imidazol-4-yl)-3,6,9-trioxa-12-azaheptadecyl)-1H-1,2,3-triazol-4-yl)methyl)-2-(2-(1,2,2,4-tetramethyl-1,2-dihydroquinolin-6-yl)vinyl)benzo[d]thiazol-3-ium 2,2,2-trifluoroacetate* **4d**


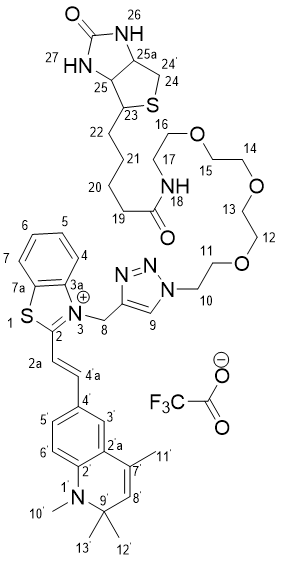


(Yield 41.9 %) ^1^H NMR (600 MHz, DMSO-*d*_6_): δ 8.32 (1H, s, **Н9**), 8.28 (1H, d, *J* = 8.8 Hz, **Н7**), 8.21 (1H, d, *J* = 8.7 Hz, **Н4**), 8.11 (1H, d, *J* = 15.0 Hz, **Н2а**), 7.86 – 7.74 (4H, m, **Н4**'**а, Н6', Н18, Н5**), 7.68 – 7.64 (1H, m, **Н6**), 7.61 – 7.60 (2H, m, **Н3'**, **Н5'**), 6.71 (1H, d, *J* = 9.0 Hz, **Н8'**), 6.38 (1H, s, **Н26**), 6.35 (1H, s, **Н27**), 6.17 (2H, s, **Н8**), 4.52 (2H, t, *J* = 5.1 Hz, **Н10**), 4.32 – 4.28 (1H, m, **Н25а**), 4.13 – 4.09 (1H, m, **Н25**), 3.77 (2H, t, *J* = 5.0 Hz, **Н11**), 3.46 – 3.35 (10H, m, **Н12-Н16**), 3.19 – 3.15 (2H, m, **Н17**), 3.12 – 3.06 (1H, m, **Н23**), 2.98 (3H, s, **Н10'**), 2.81 (1H, dd, *J* = 12.4, 5.2 Hz, **Н24**), 2.57 – 2.54 (1H, m, **Н24**'), 2.08 – 2.04 (2H, m, **Н19**) 2.03 (3H, s, **Н11**'), 1.51 – 1.41 (6H, m, **Н20- Н22**), 1.40 (6H, s, **Н13', Н12'**). HRMS (ESI) m/z: calcd for C_43_H_57_N_8_O_5_S_2_^+^ [M-CF_3_COO^-^]^+^: 829.3888; found 829.3924; t_R_ = 4.67 min.

# Figures and Tables


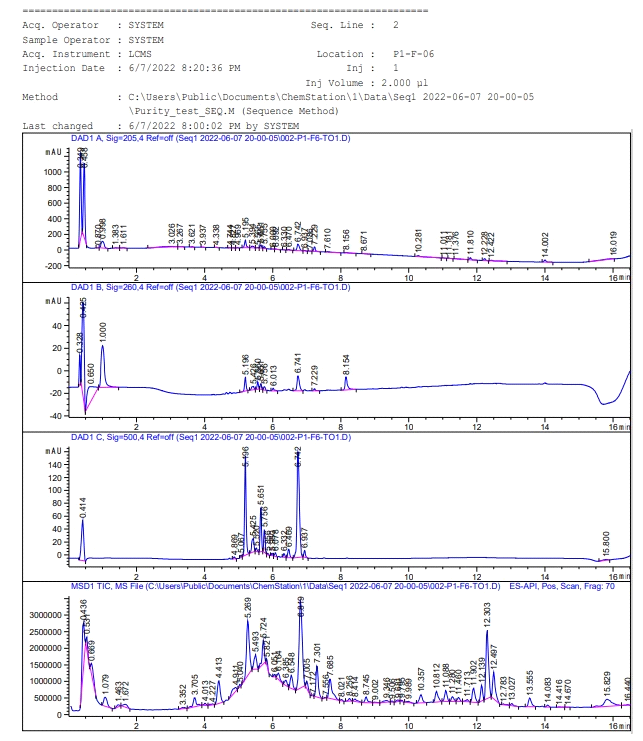


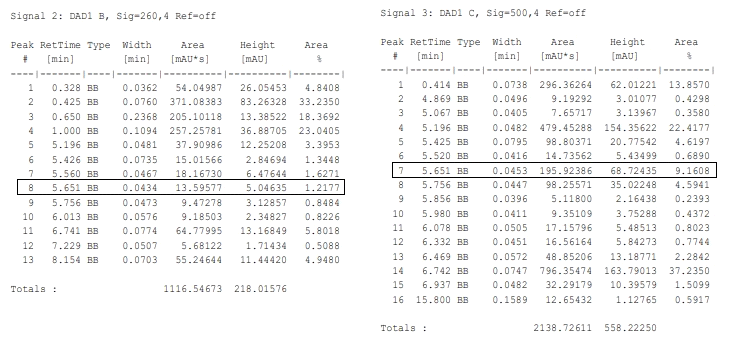


## Fig. S1. HPLC profile and purity assessment (Area %) of TO1-biotin


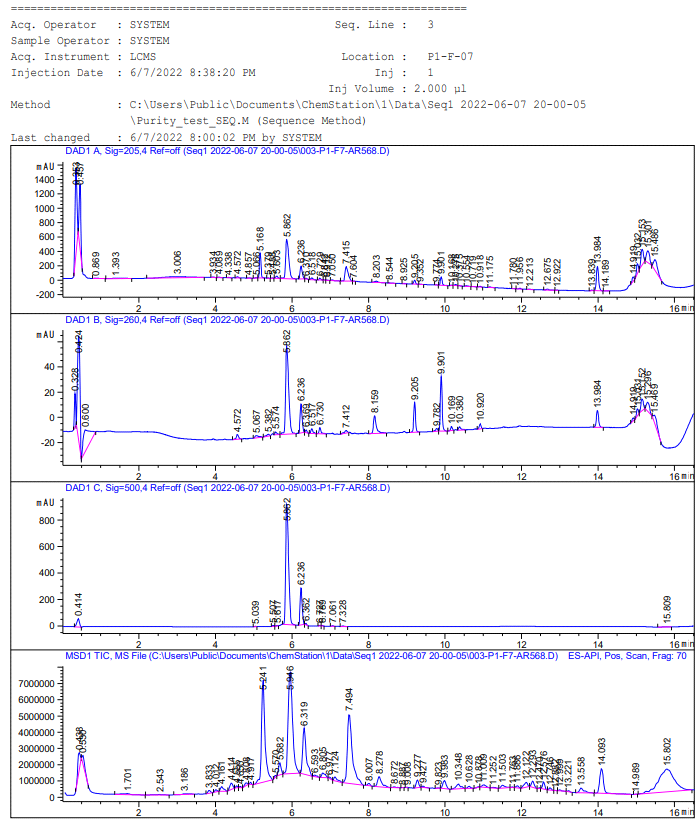


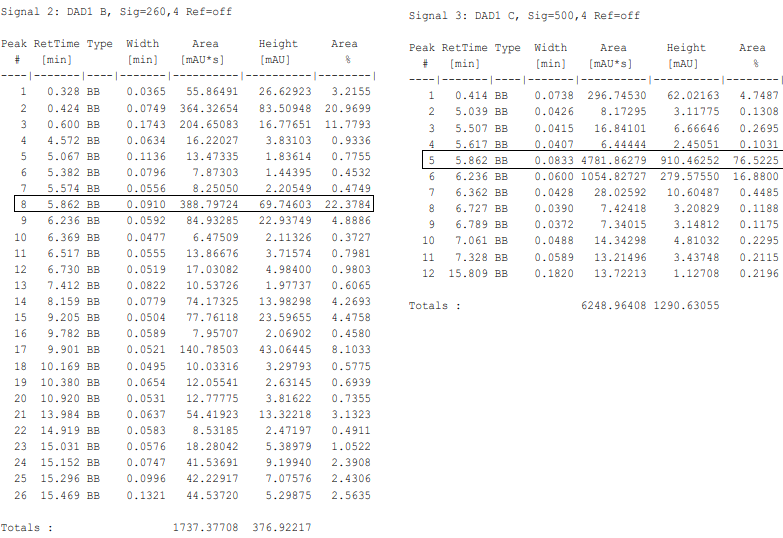


## Fig. S2. HPLC profile and purity assessment (Area %) of 4a

Table S1. Sequences of chemically synthesized oligonucleotides used for evaluating the spectral properties, affinity and selectivity of fluorogenic dyes.

| **code** | **Sequence, 5'→3'** |
| --- | --- |
| Mango-II | r(GCACGUACGAAGGAGAGGAGAGGAAGAGGAGAGUACGUGC) |
| Mango-IV | r(GCACGUACCGAGGGAGUGGUGAGGAUGAGGCGAGUACGUGC) |
| G4 RNA (utr-z) | r(GGCGGCGGCAGUGGCGGCGG) |
| dsRNA (hairpin ds26) | r(CAAUCGGAUCGAAUUCGAUCCGAUUG) |
| dsDNA (hairpin ds26) | d(CAATCGGATCGAATTCGATCCGATTG) |
| ssDNA (dT_18_) | d(TTTTTTTTTTTTTTTTTT) |
| h/pG4-DNA (hybrid/parallel G4 22AG) | d(AGGGTTAGGGTTAGGGTTAGGG) |
| aG4 (antiparallel G4 22CTA) | d(AGGGCTAGGGCTAGGGCTAGGG) |
| i-motif (hexanucleotide repeat) | d(CCCCGGCCCCGGCCCCGGCCCC) |

**
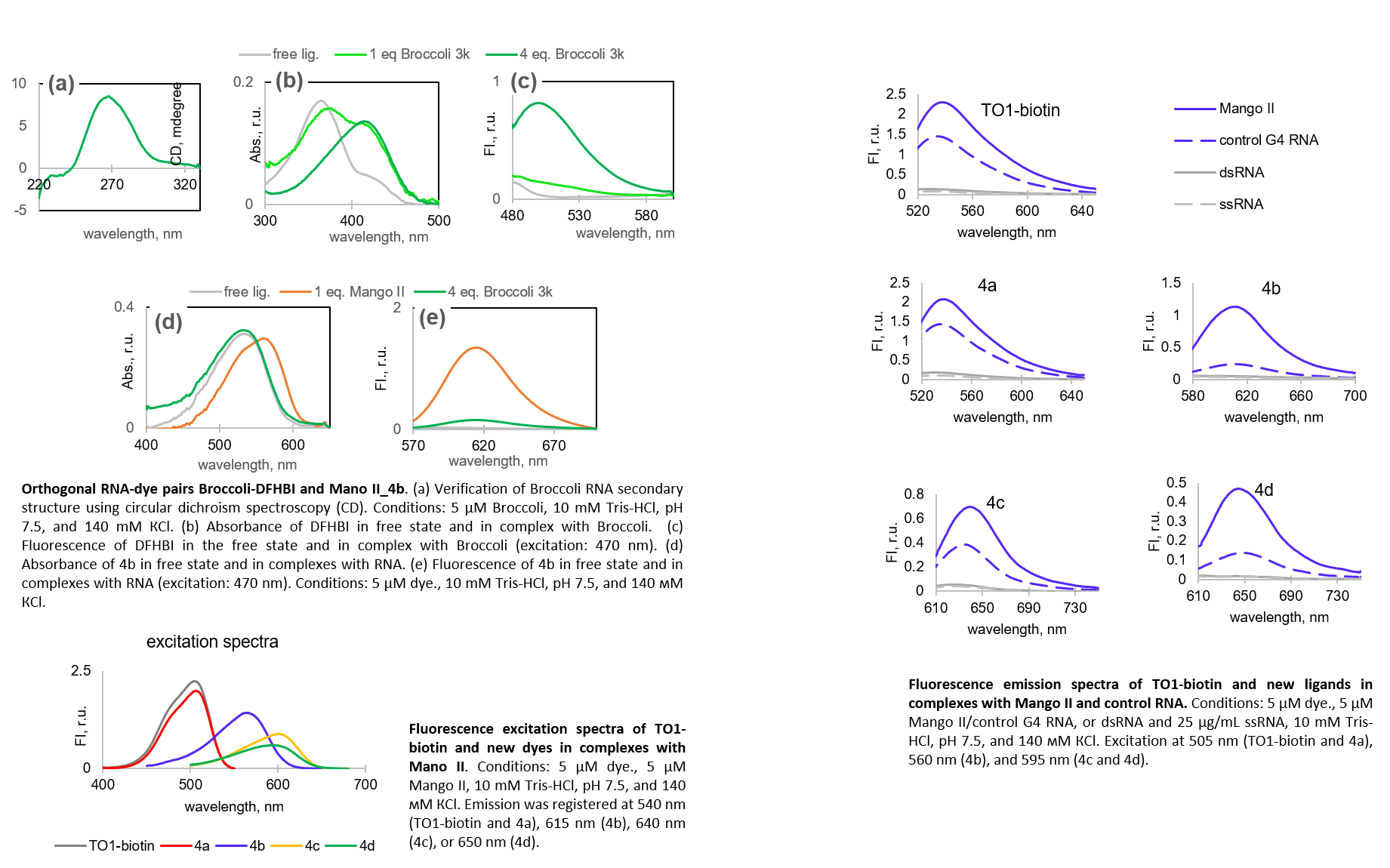
**

Fig. S3. Fluorescence excitation spectra of TO1-biotin and new dyes 4a-d in complexes with Mango II**.** Conditions: 5 µM dye, 5 µM Mango II, 10 mM Tris-HCl, pH 7.5, and 140 мМ КСl. Emission was registered at 540 nm (TO1-biotin and **4a**), 615 nm (**4b**), 640 nm (**4c**), or 650 nm (**4d**).

**
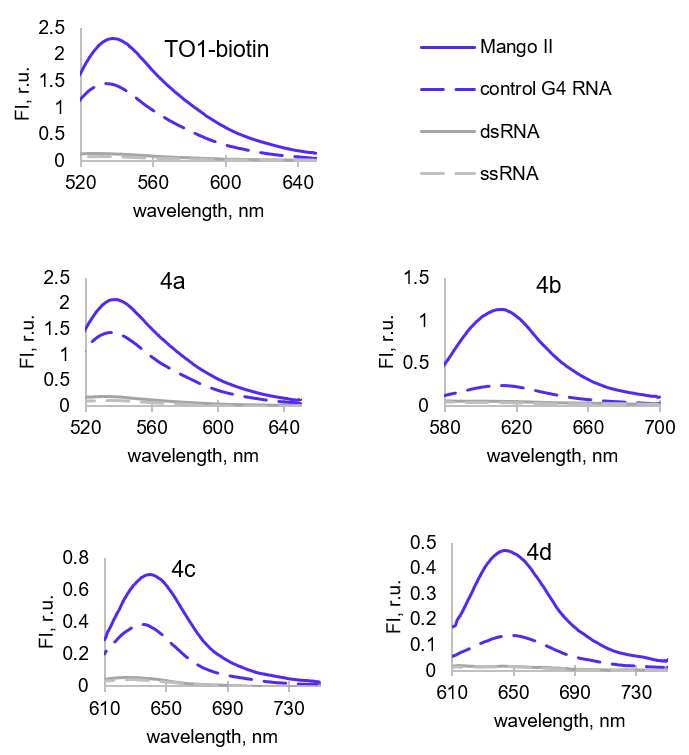
**

Fig. S4. Fluorescence emission spectra of TO1-biotin and new ligands 4a-d in complexes with Mango II and control RNA**.** Conditions: 5 µM dye, 5 µM Mango II/control G4 RNA, or dsRNA and 25 µg/mL ssRNA, 10 mM Tris-HCl, pH 7.5, and 140 мМ КСl. Excitation at 505 nm (TO1-biotin and **4a**), 560 nm (**4b**), and 595 nm (**4c** and **4d**).


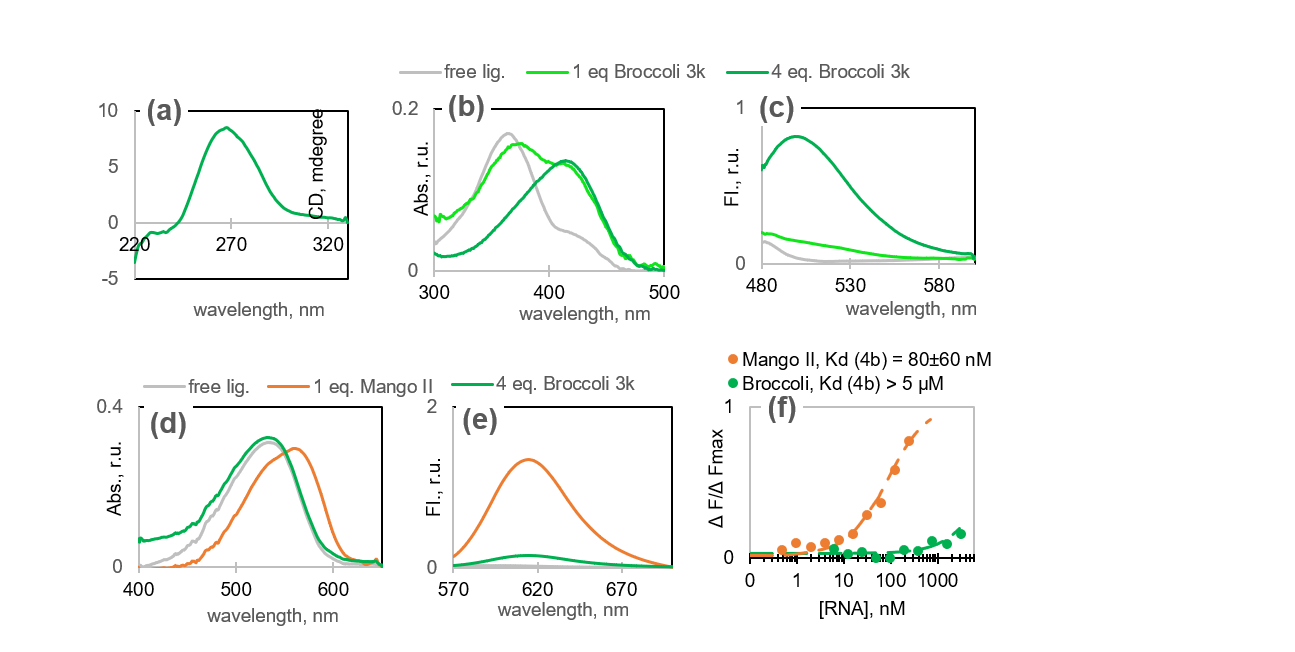


Fig. S5. Orthogonal RNA-dye pairs DFHBI-Broccoli and 4b-Mango II**.** (a) Verification of Broccoli RNA secondary structure using circular dichroism spectroscopy (CD). Conditions: 5 µM Broccoli, 10 mM Tris-HCl, pH 7.5, and 140 mМ КСl. (b) Absorbance of **DFHBI** in free state and in complex with Broccoli. (c) Fluorescence of DFHBI in free state and in complex with Broccoli (excitation: 470 nm). (d) Absorbance of **4b** with or without RNA aptamers. (e) Fluorescence of **4b** with or without RNA aptamers (excitation: 560 nm). Conditions: 5 µM dye, 10 mM Tris-HCl, pH 7.5, and 140 mM КСl. (f) Comparison of **4b** interactions with Mango II and Broccoli. Conditions: 10 nM **4b**, 10 mM Tris-HCl, pH 7.5, and 140 mM КСl, 0,05% Tween-20.


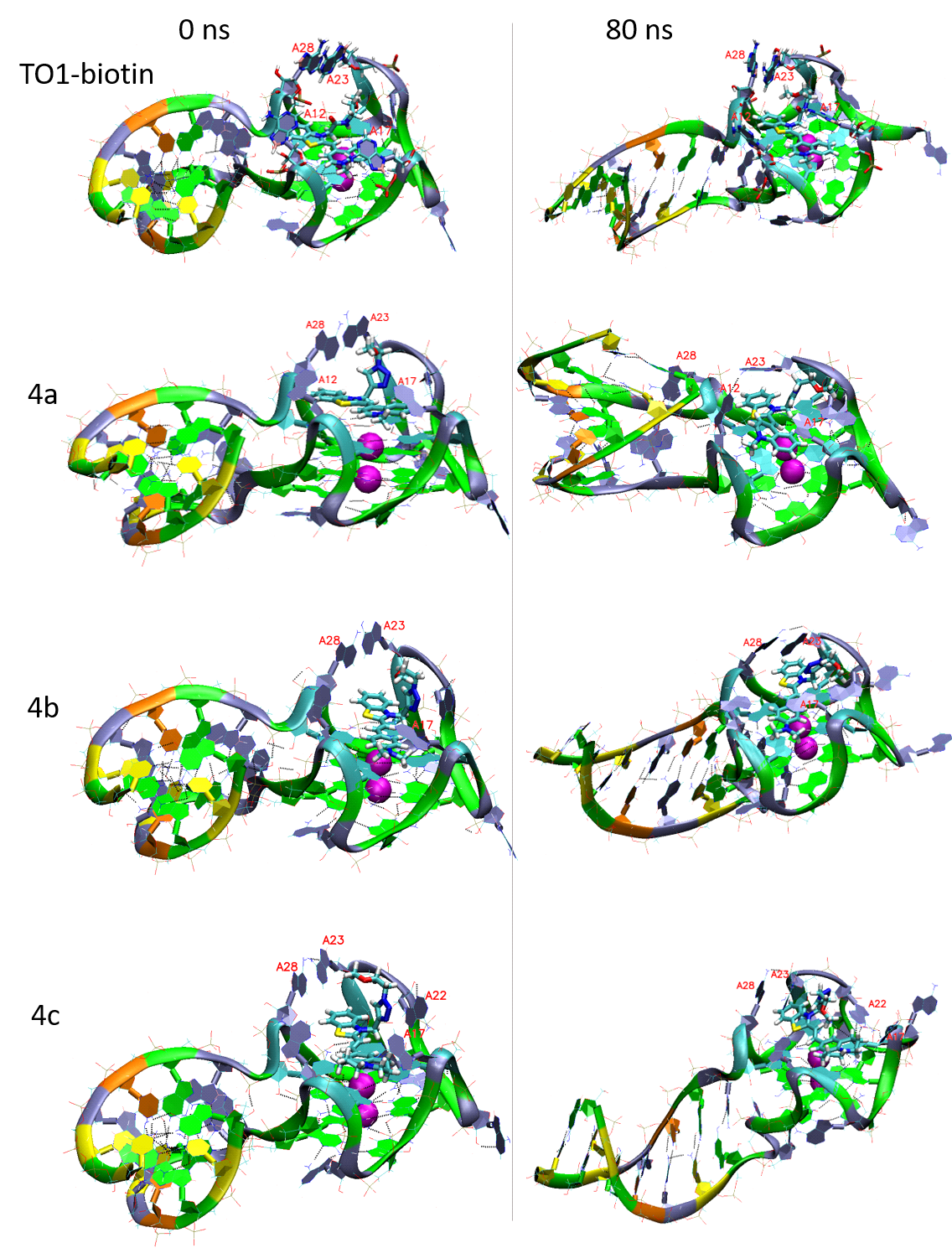


Fig. S6. Molecular dynamics of dye-Mango II complexes: full structures of the complexes at 0 and 80 ns simulation times. Color labeling of aptamer residues: G, green; A, grey; T, orange; C, cyan. Ligand atoms: C, cyan; N, blue; O, red; S, yellow.


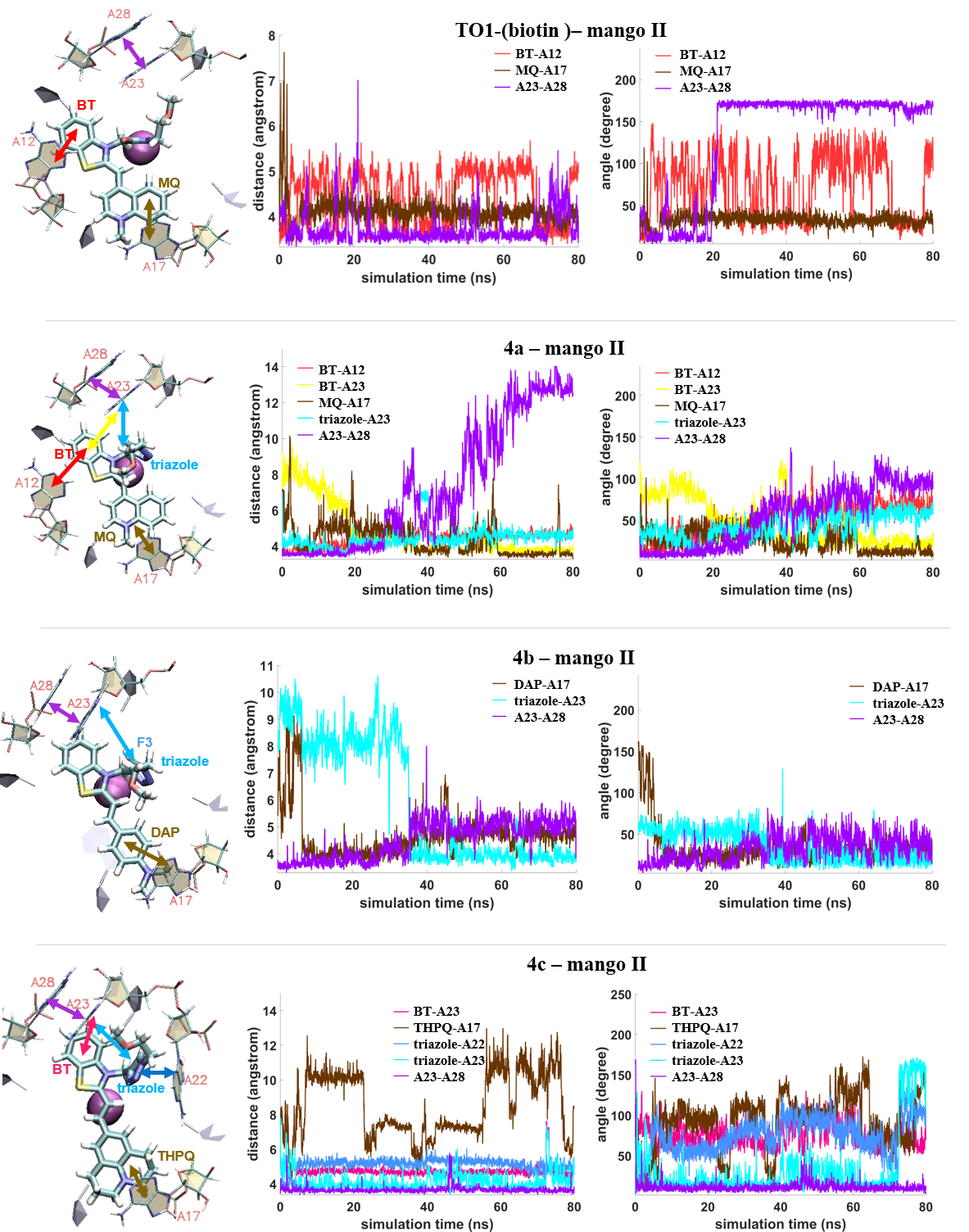


Fig. S7. Molecular dynamics of dye-Mango II complexes: binding modes. Left: dye positioning in complex with mango II. Arrows mark distances between COMs of key dye and aptamer residues. Middle: distances between the centers of mass (COM) of the specified residues. Right: angles between the normal to the planes of these residues. BT, benzothiazolium ring; MQ, N-methylquinolinyl group; DAP, *N*,*N*-dimethylaminophenyl group; THPQ, 2,3,6,7-tetrahydro-H,5H-pyrido[3,2,1-ij]quinolinyl group. In each pair of the residues, only the closest rings were considered, and for these rings COM distances and angles were analyzed.


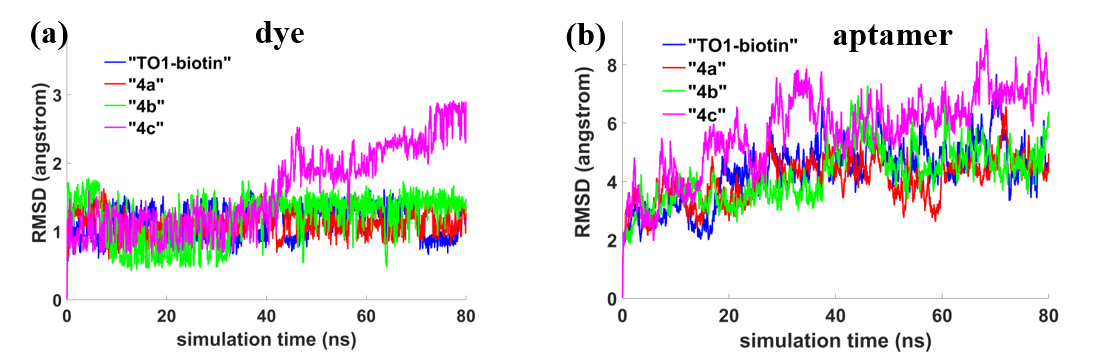


Fig. S8. Molecular dynamics of dye-Mango II complexes: RMSD of the dyes (a) and the aptamer (b)**.**


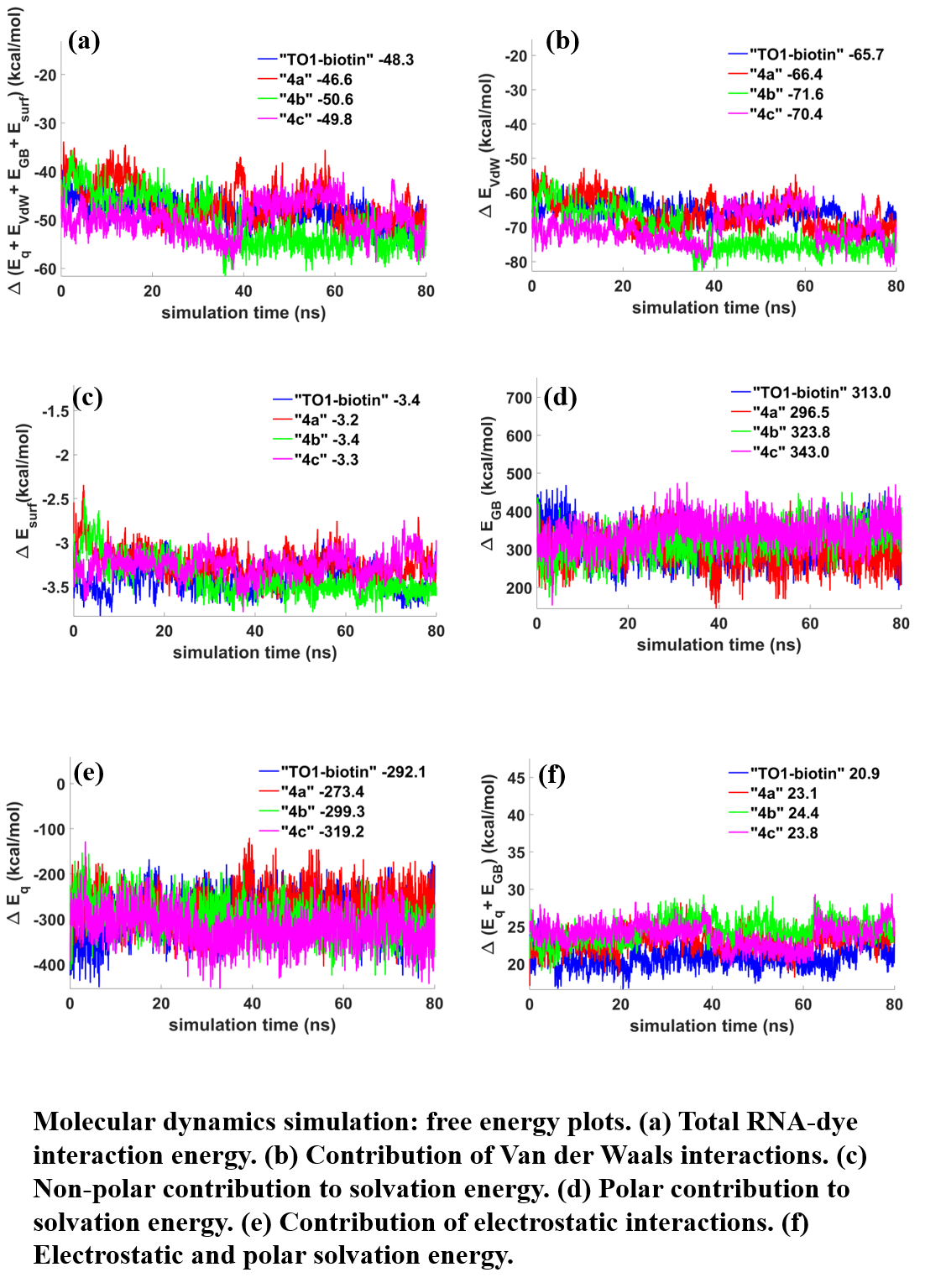


Fig. S9. Molecular dynamics simulation: free energy plots. (a) Total RNA-dye interaction energy. (b) Contribution of Van der Waals interactions. (c) Non-polar contribution to solvation energy. (d) Polar contribution to solvation energy. (e) Contribution of electrostatic interactions. (f) Electrostatic and polar solvation energy.


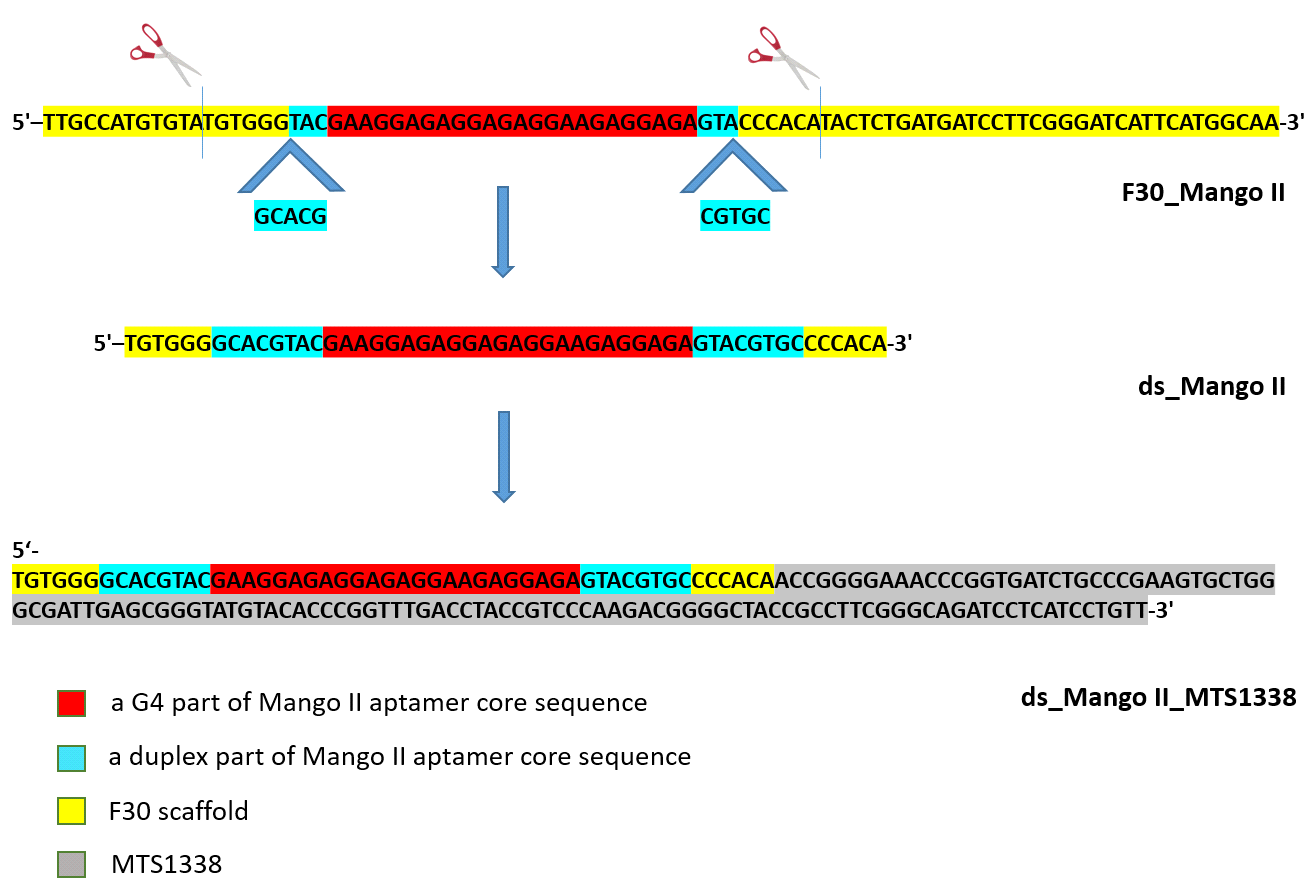


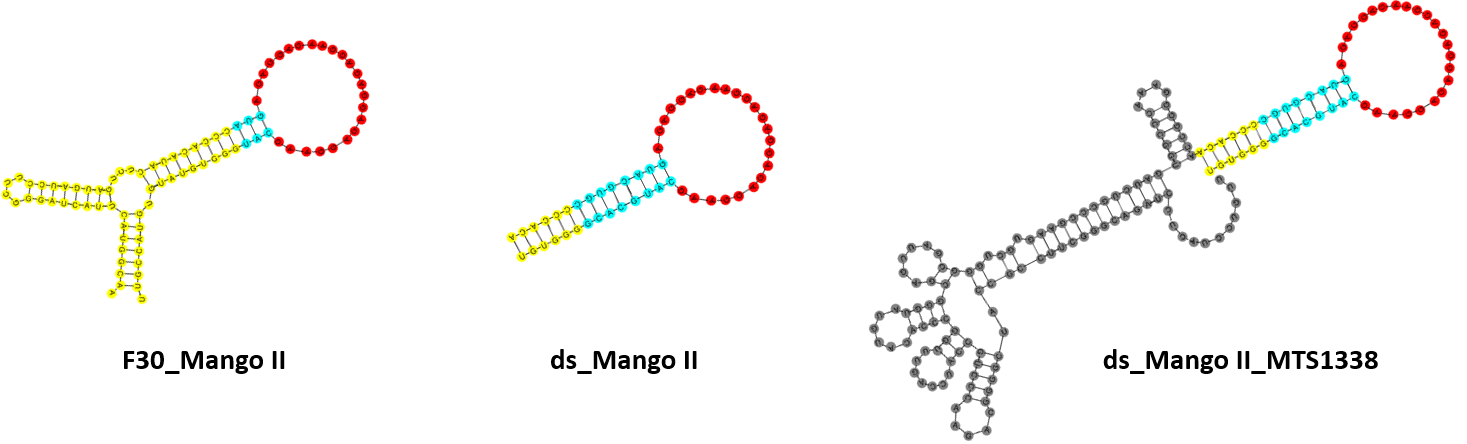


Fig. S10. The sequences and the secondary structure of F30_Mango II, ds_Mango II aptamers, and modular RNA ds_Mango II_MTS1338 according to the RNAfold server (<http://rna.-tbi.univie.ac.at/cgi-bin/RNAWebSuite/RNAfold.cgi>).

## Table S2. Nucleotide sequences of the genetic constructs and the oligonucleotides used for their analysis

| **code** | **sequence 5'→3'** |
| --- | --- |
| rrnB promoter | CTAGAGTGACCGCGTCTGACCAGGGAAAATAGCCCTCTGACCTGGGGATTTGACTCCCAGTTTCCAAGGACGTAACTTA |
| MTS1338 | ACCGGGGAAACCCGGTGATCTGCCCGAAGTGCTGGGCGATTGAGCGGGTATGTACACCCGGTTTGACCTACCGTCCCAAGACGGGGCTACCGCCTTCGGGCAGATCCTCATCCTGTT |
| term terminator | GCTTCCCCGCGAAAGCGGGGTTTTTTTTTTTAGCT |
| ds_Mango II | TGTGGGGCACGTACGAAGGAGAGGAGAGGAAGAGGAGAGTACGTGCCCCACA |
| Broccoli | GAGACGGTCGGGTCCAGATATTCGTATCTGTCGAGTAGAGTGTGGGCTC |
| rrnB_ds_Mango II_term | CTAGAGTGACCGCGTCTGACCAGGGAAAATAGCCCTCTGACCTGGGGATTTGACTCCCAGTTTCCAAGGACGTAACTTATGTGGGGCACGTACGAAGGAGAGGAGAGGAAGAGGAGAGTACGTGCCCCACACCCCGCGAAAGCGGGGTTTTTTTTTTTAGCT- |
| rrnB_ds_Mango II_MTS1338_term | CTAGAGTGACCGCGTCTGACCAGGGAAAATAGCCCTCTGACCTGGGGATTTGACTCCCAGTTTCCAAGGACGTAACTTATGTGGGGCACGTACGAAGGAGAGGAGAGGAAGAGGAGAGTACGTGCCCCACAACCGGGGAAACCCGGTGATCTGCCCGAAGTGCTGGGCGATTGAGCGGGTATGTACACCCGGTTTGACCTACCGTCCCAAGACGGGGCTACCGCCTTCGGGCAGATCCTCATCCTGTTCCCCGCGAAAGCGGGGTTTTTTTTTTTAGCT |
| rrnB_Broccoli _term | CTAGAGTGACCGCGTCTGACCAGGGAAAATAGCCCTCTGACCTGGGGATTTGACTCCCAGTTTCCAAGGACGTAACTTAGAGACGGTCGGGTCCAGATATTCGTATCTGTCGAGTAGAGTGTGGGCTCCCCCGCGAAAGCGGGGTTTTTTTTTTTAGCT |
| T7MngII_F | GTTTTTTTTAATACGACTCACTATAGGTGTGGGGCACGTAC |
| MngII_R | TGTGGGGCACGTACTCTCCT |
| 1338_R | AACAGGATGAGGATCTGCCCG |
| T7Broc_F | GTTTTTTTTAATACGACTCACTATAGGGAGACGGTCGGGTC |
| Broc_R | GAGCCCACACTCTACTCGACAGATACGAAT |
| q1338-F | GTGCTGGGCGATTGAGC |
| q1338-R | GCGGTAGCCCCGTCTT |
| 16S-F | TACGTAGGGTGCGAGCGTTG |
| 16S-R | CCCGCACGCTCACAGTTAAG |

**
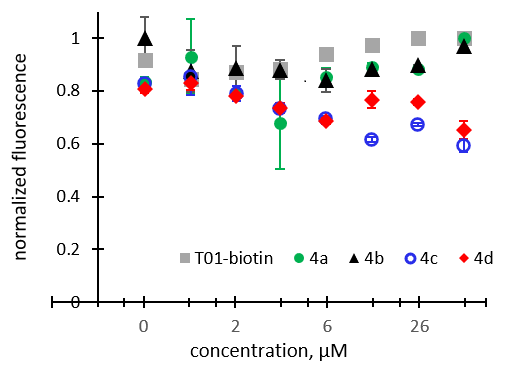
**

Fig. S11. Cytotoxicity of TO1-biotin and 4a-d in RAW 264.7 cell lines after 7 d incubation**.**

Fig. S12. Evaluation of the dye cytotoxicity for bacterial cells using optical density (OD_600_) measurements. Growth curves of *M. smegmatis*_pAMYC in the presence of **4b** in concentrations 400 nM, 1 µM and without dye (positive control). The data are presented as the mean +-SD of three independent experiments.


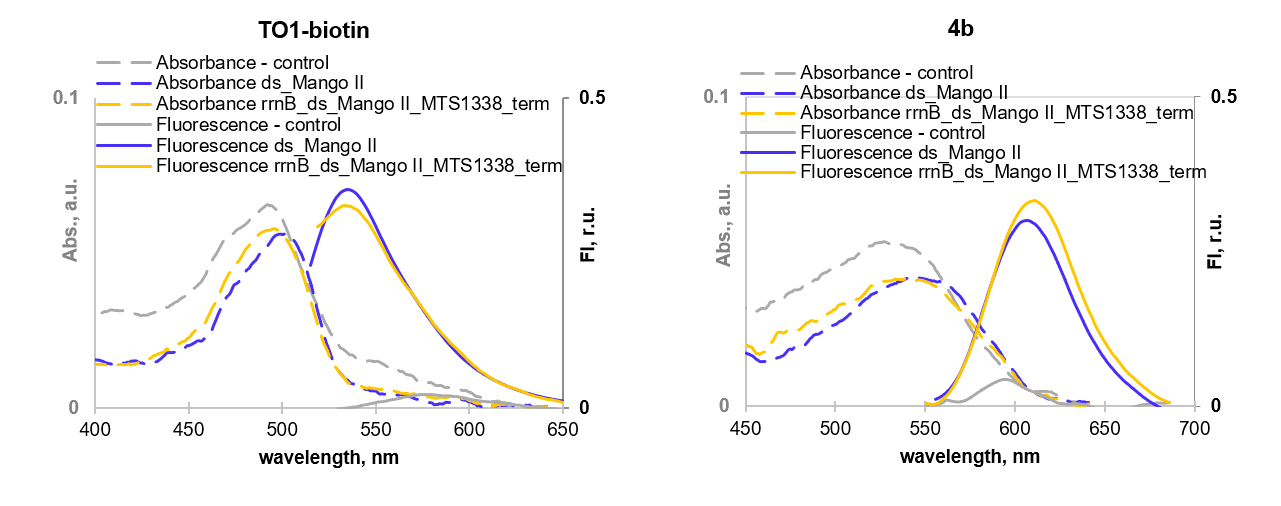


Fig. S13. Spectra of TO1-biotin and 4b in complexes with the Mango II-labeled genetic construct and control RNA**.** Absorption and fluorescence emission spectra of the dyes in complexes with rrnB_Mango II_MTS1338_term, ds_Mango II, and random-sequence Mango-free (-control) RNA. Conditions: 1 μM RNA, 1 μM dye, 20 mM sodium phosphate buffer, pH 7.2, 0.05% Tween-20, and 140 mM КСl.

**
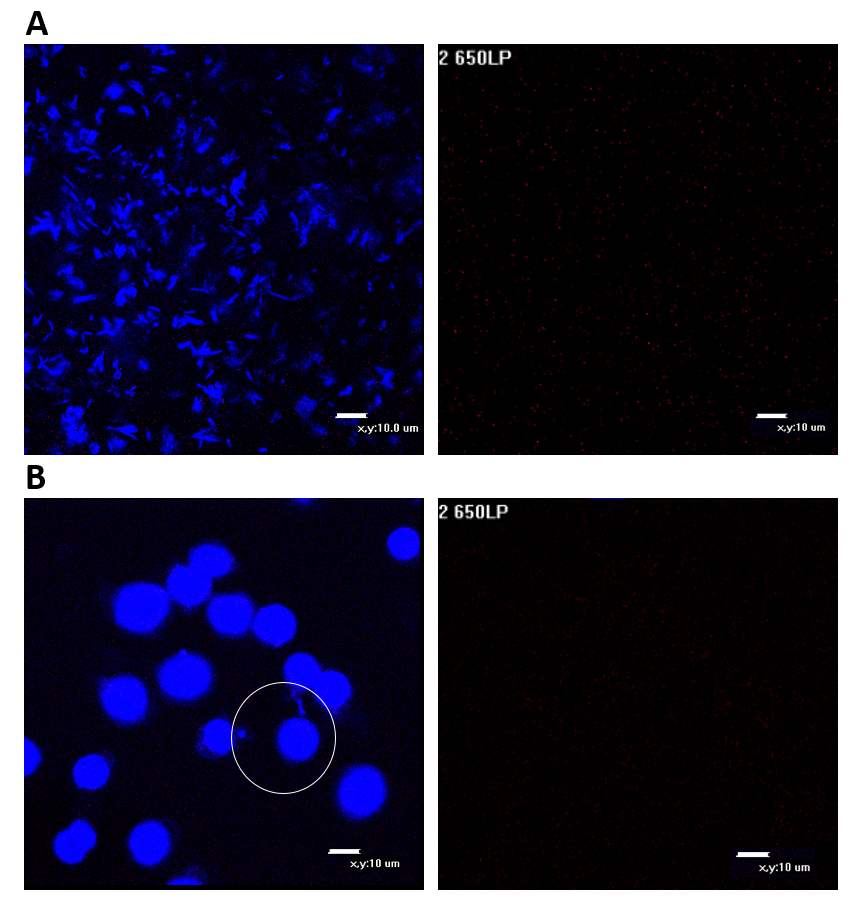
**

Fig S14. The control confocal microscopy images showing 4b staining of Msm_pAMYC in buffer (A) and infected macrophages RAW 264.7 (B). Two confocal microscopy panels (blue and red channels) are given. Macrophage nuclei and bacteria were stained with Hoechst 33258 (in a blue channel). The circle depicts phagocytosed bacteria.


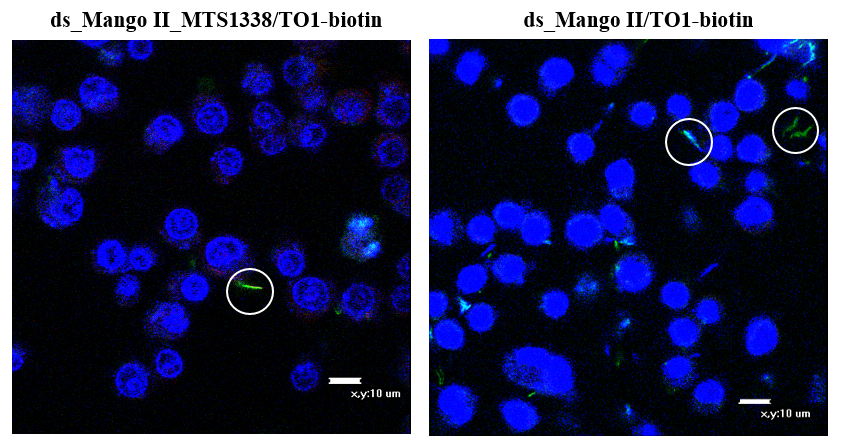


Fig S15. Visualization of the genetically encoded modular RNA ds_Mango II_MTS1338 and ds_Mango II tag in *M. smegmatis* in infected RAW 264.7 macrophages using TO1-biotin (in a green channel). Macrophage nuclei were stained with Hoechst 33258 (in a blue channel). The circles cover fluorescent bacteria.


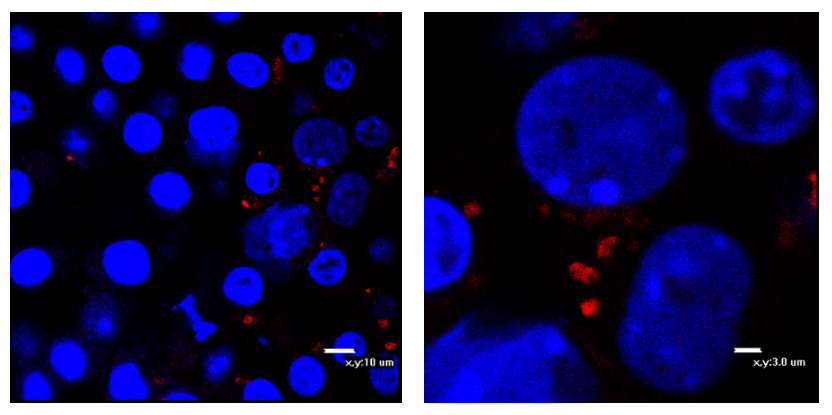


Fig S16. Visualization of the genetically encoded modular RNA ds_Mango II_MTS1338 using 4b in infected macrophages RAW 264.7**.** Macrophage nuclei were stained with Hoechst 33258.NMR spectra

*2-((1-methylquinolin-4(1H)-ylidene)methyl)-3-(prop-2-yn-1-yl)benzo[d]thiazol-3-ium bromide* **2a**

^1^H spectrum


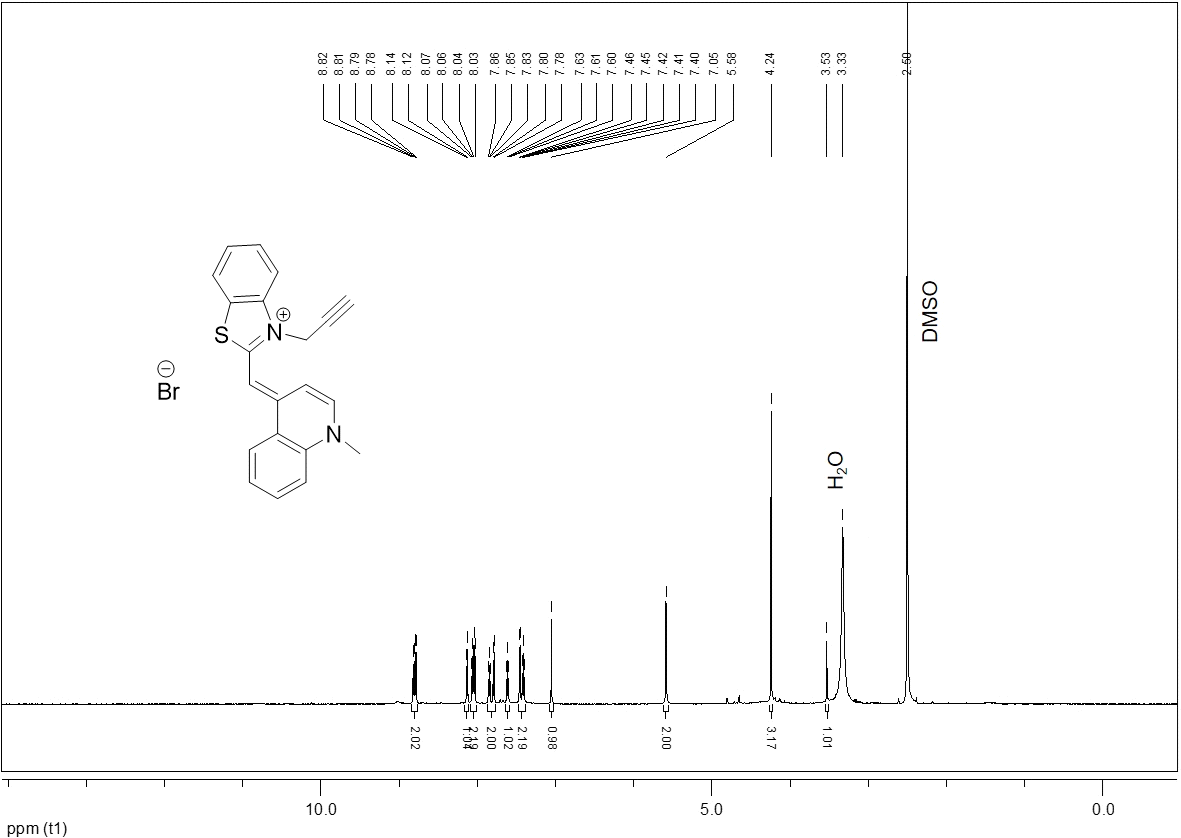


^13^C spectrum


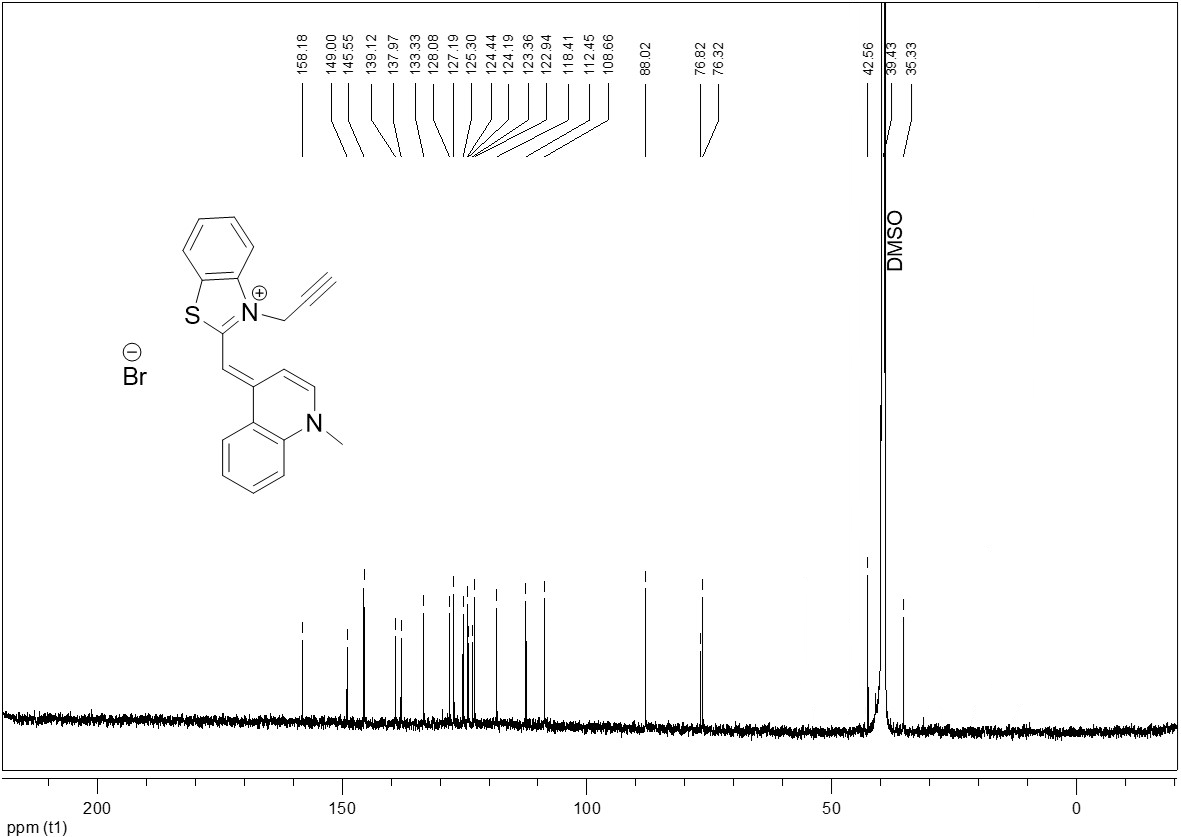


*2-(2-(4-(Dimethylamino)phenyl)ethenyl)-3-(2-propynyl)benzo[d]thiazolium bromide* **2b**

^1^H spectrum


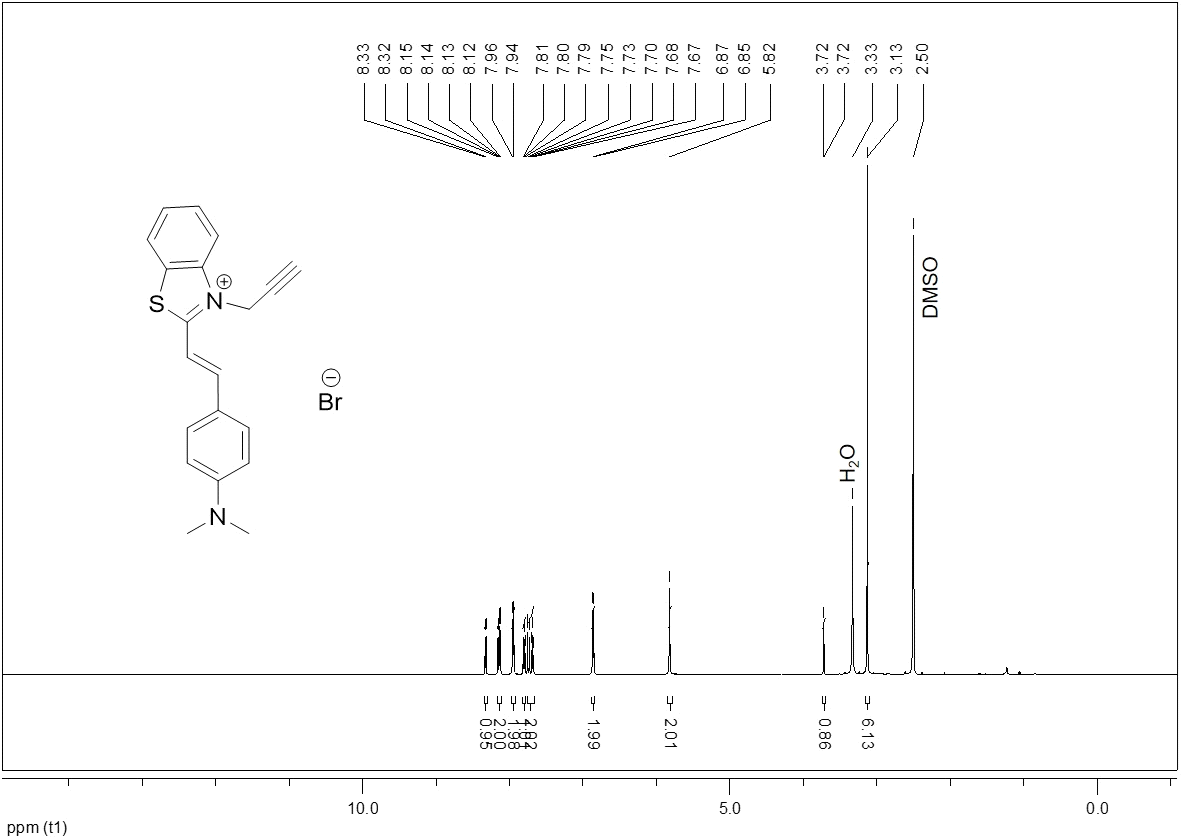


^13^C spectrum


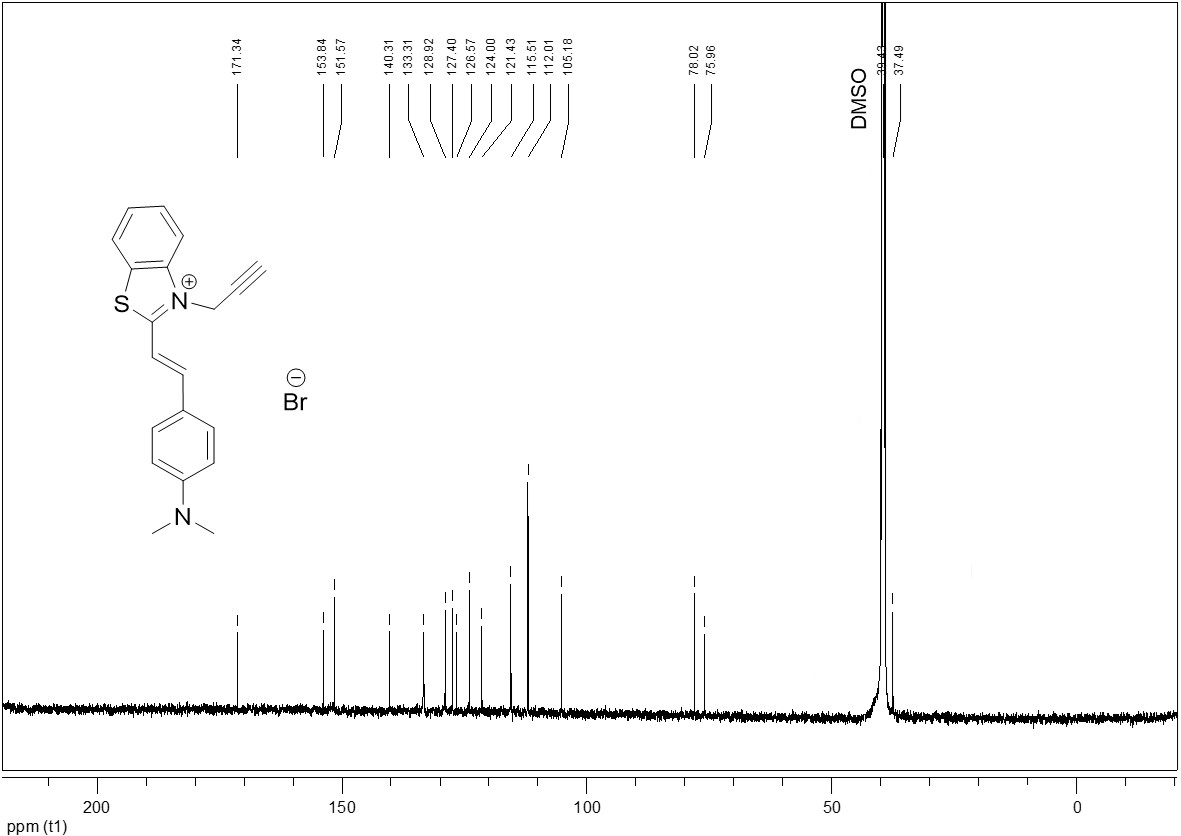


*3-(prop-2-yn-1-yl)-2-(2-(2,3,6,7-tetrahydro-1H,5H-pyrido[3,2,1-ij]quinolin-9-yl)vinyl)benzo[d]thiazol-3-ium bromide* **2c**

^1^H spectrum


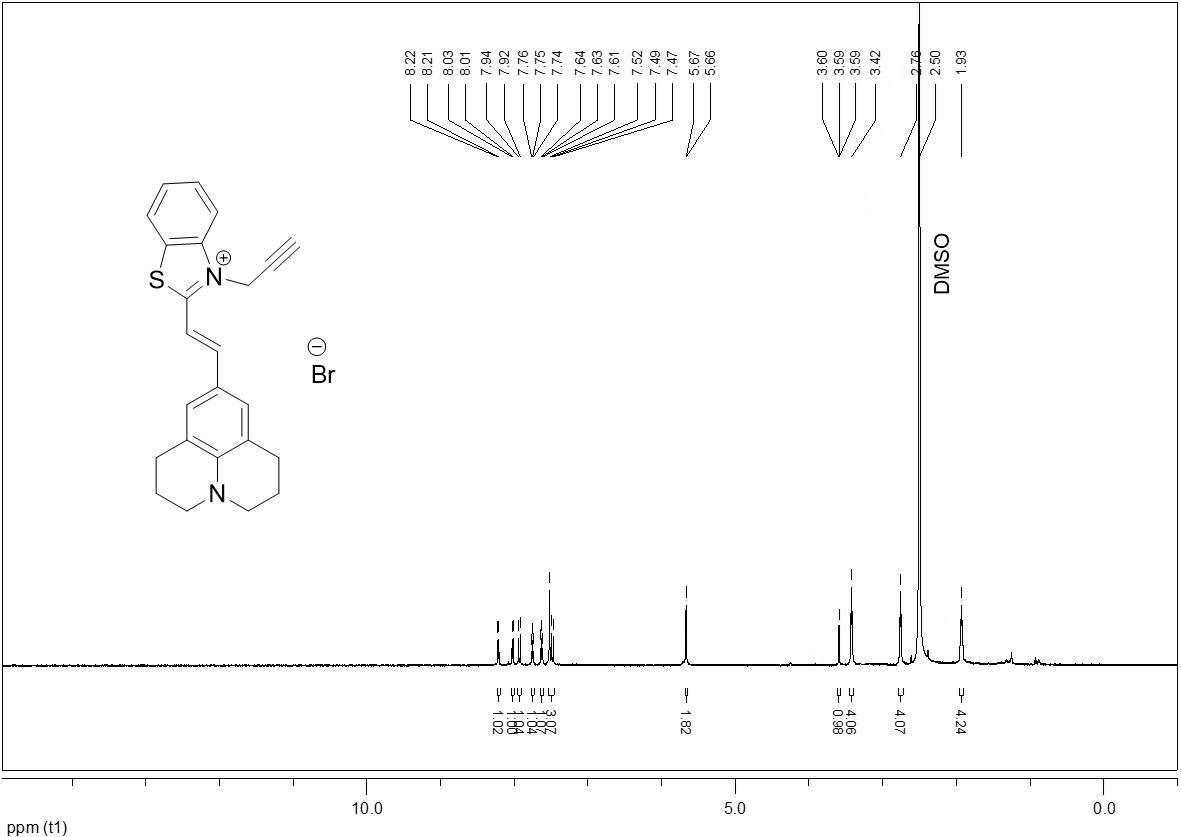


*3-(prop-2-yn-1-yl)-2-(2-(1,2,2,4-tetramethyl-1,2-dihydroquinolin-6-yl)vinyl)benzo[d]thiazol-3-ium bromide* **2d**

^1^H spectrum


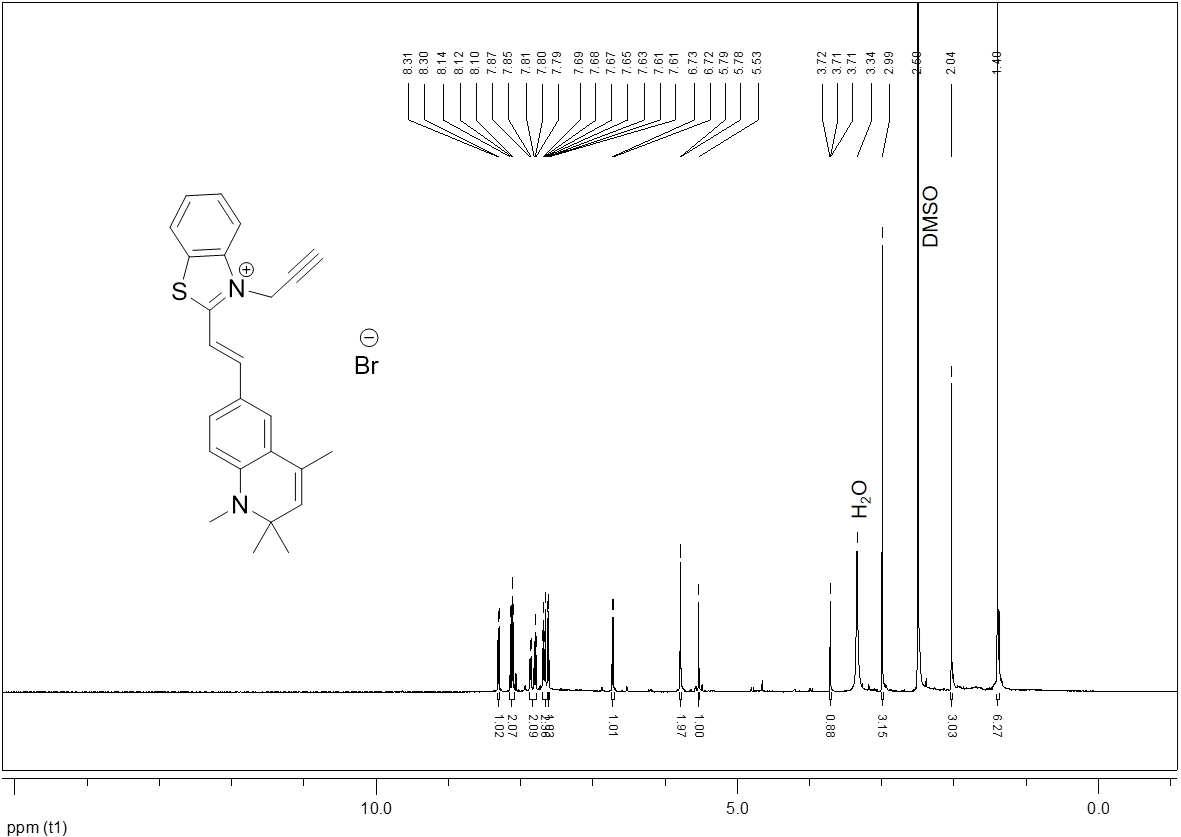


^13^C spectrum


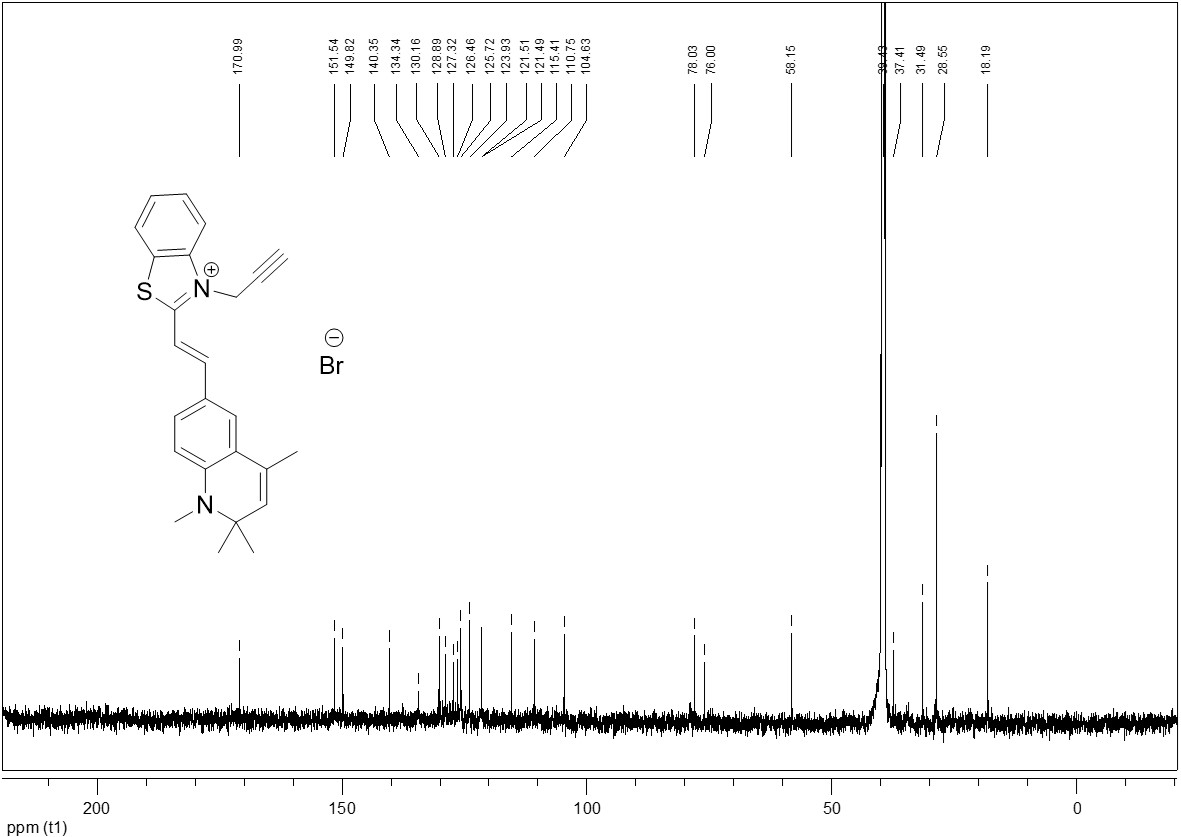


*N-(2-(2-(2-(2-azidoethoxy)ethoxy)ethoxy)ethyl)-5-(2-oxohexahydro-1H-thieno[3,4-d]imidazol-4-yl)pentanamide* **3**

^1^H spectrum


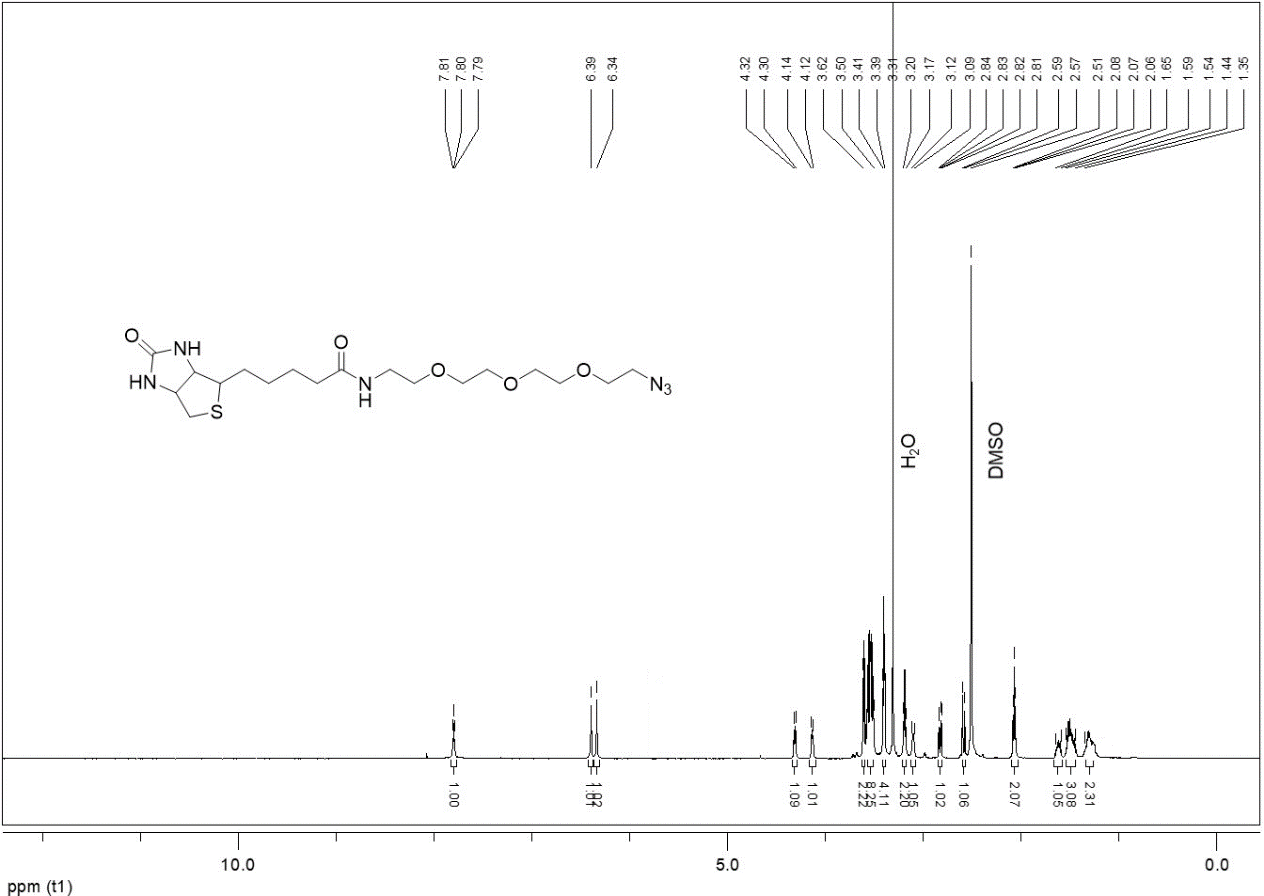


^13^C spectrum


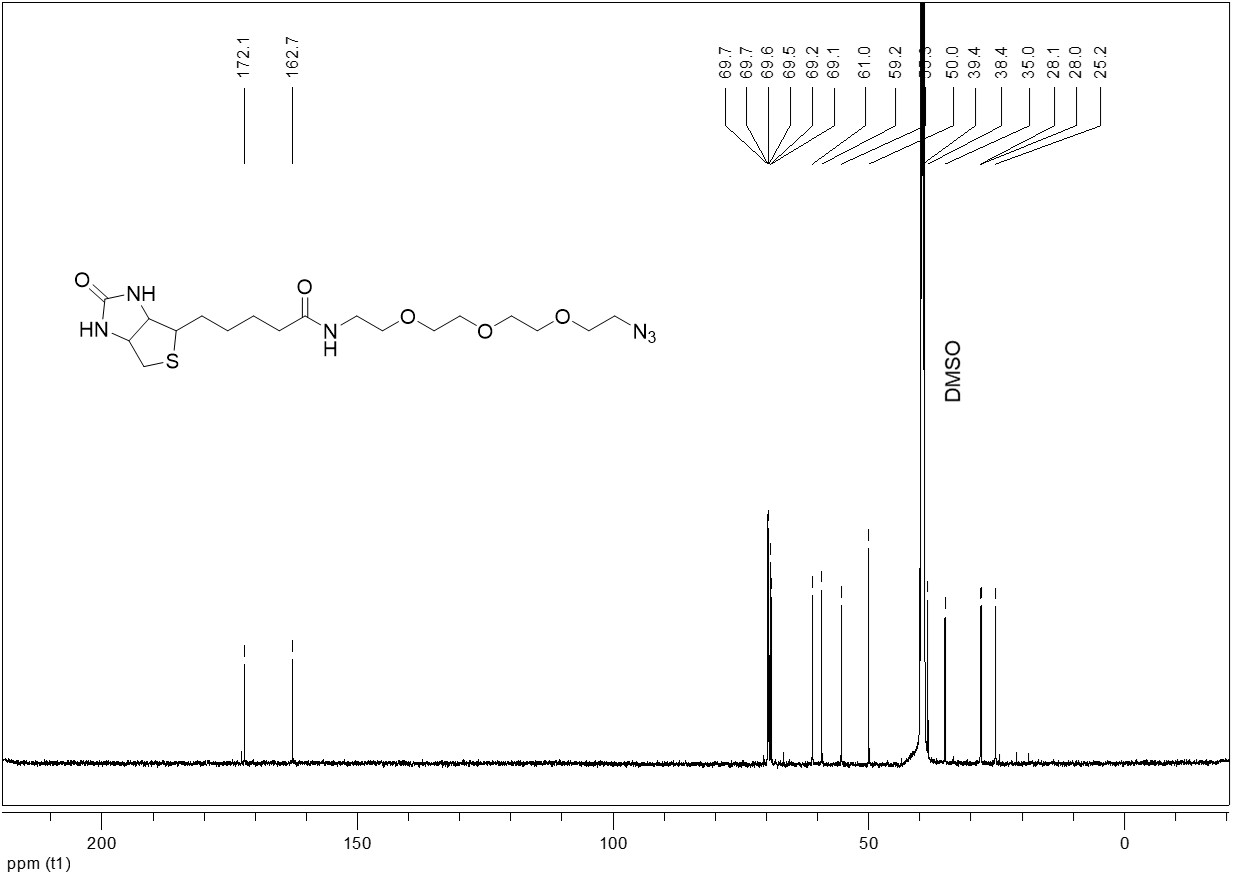


*2-((1-methylquinolin-4(1H)-ylidene)methyl)-3-((1-(13-oxo-17-(2-oxohexahydro-1H-thieno[3,4-d]imidazol-4-yl)-3,6,9-trioxa-12-azaheptadecyl)-1H-1,2,3-triazol-4-yl)methyl)benzo[d]thiazol-3-ium 2,2,2-trifluoroacetate* **4a**


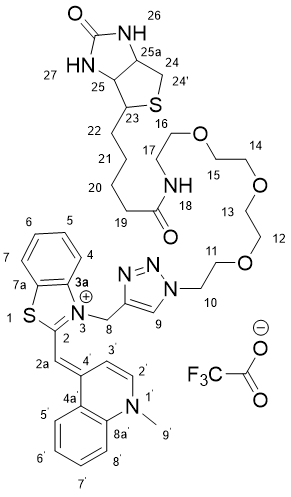


^1^H spectrum


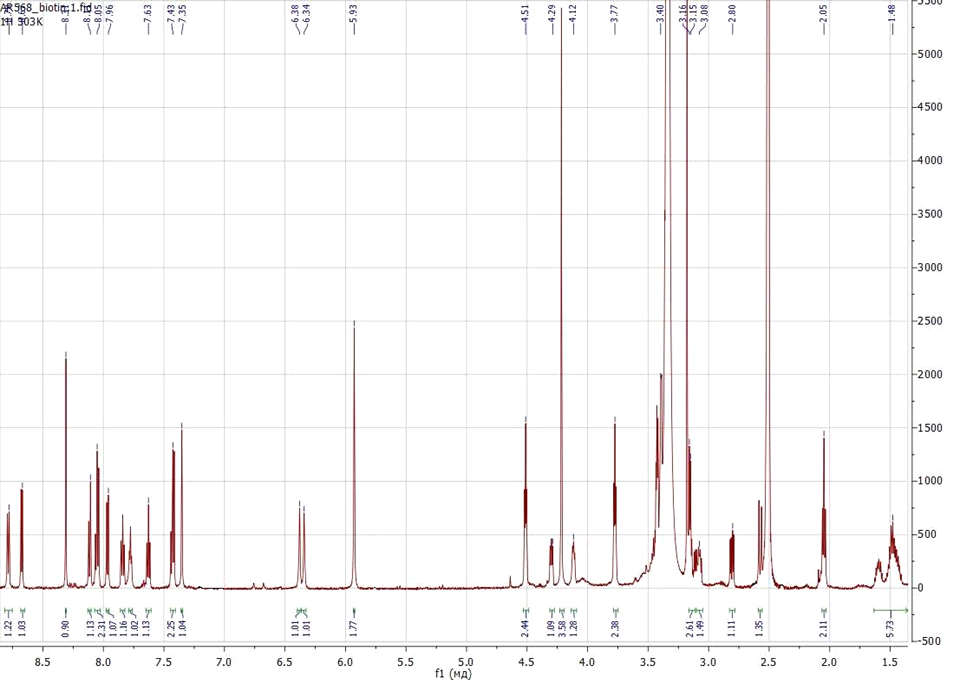


An expansion of the 2D ^1^H COSY NMR spectrum (aromatic region)


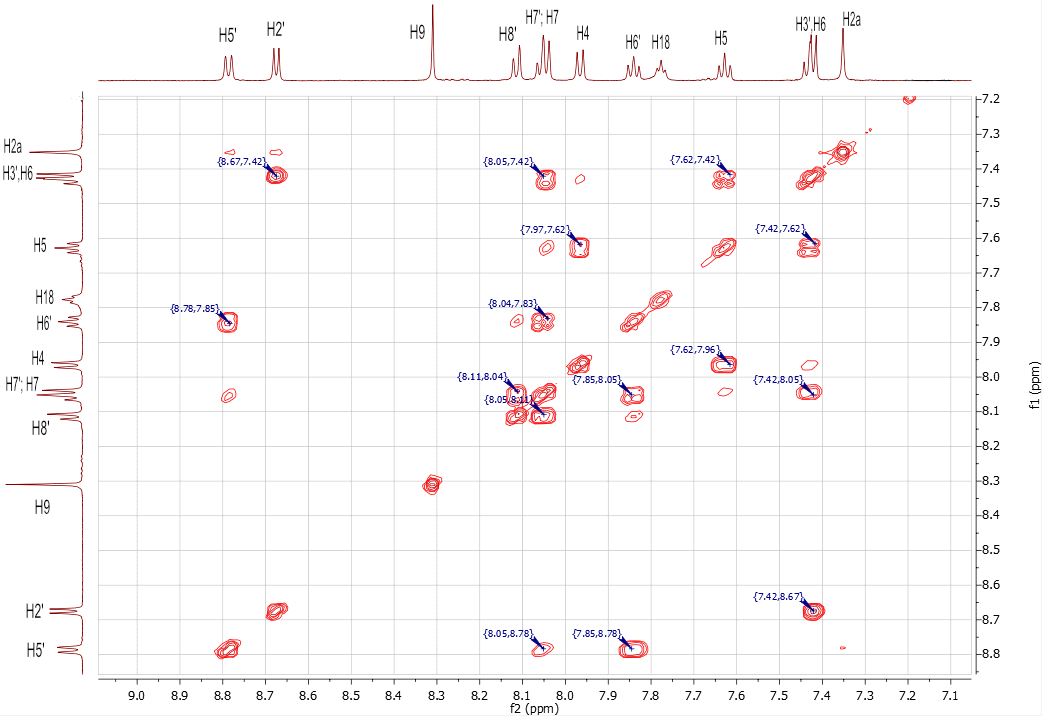


An expansion of the 2D ^1^H COSY NMR spectrum (aliphatic region)


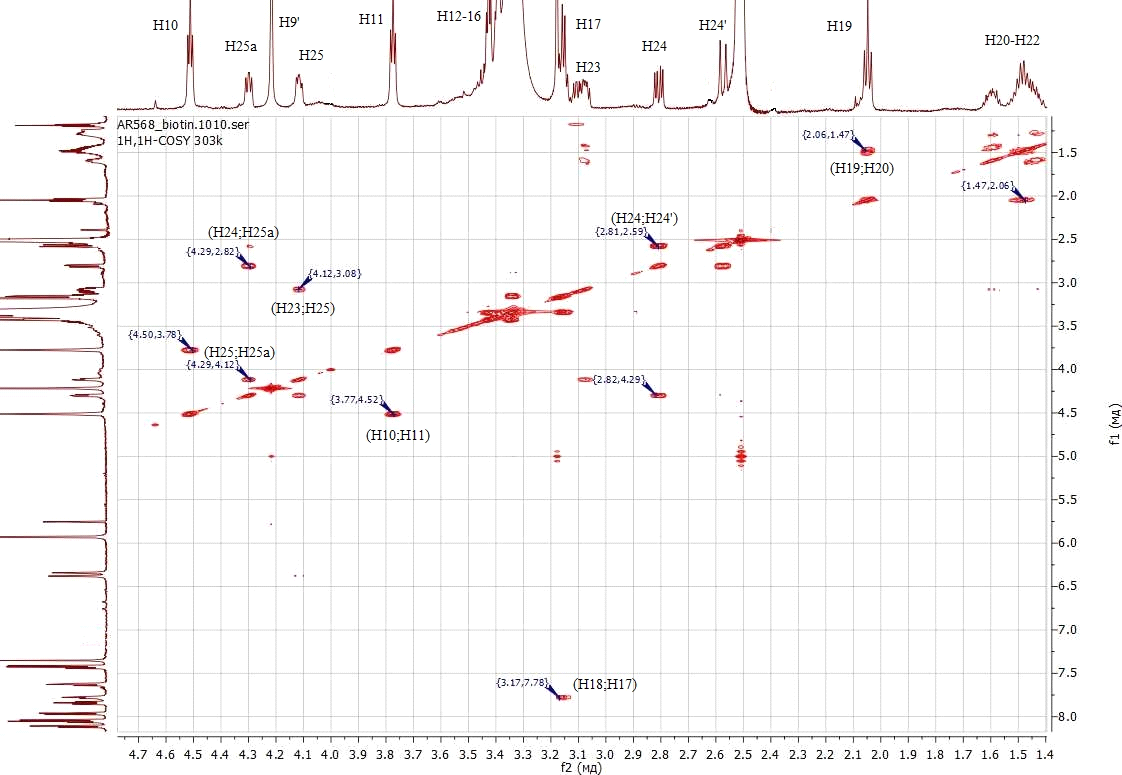


*2-(4-(dimethylamino)styryl)-3-((1-(13-oxo-17-(2-oxohexahydro-1H-thieno[3,4-d]imidazol-4-yl)-3,6,9-trioxa-12-azaheptadecyl)-1H-1,2,3-triazol-4-yl)methyl)benzo[d]thiazol-3-ium 2,2,2-trifluoroacetate* **4b**


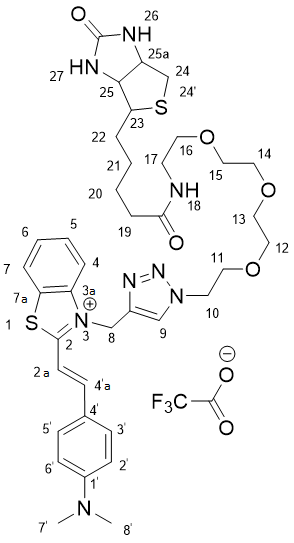


^1^H spectrum


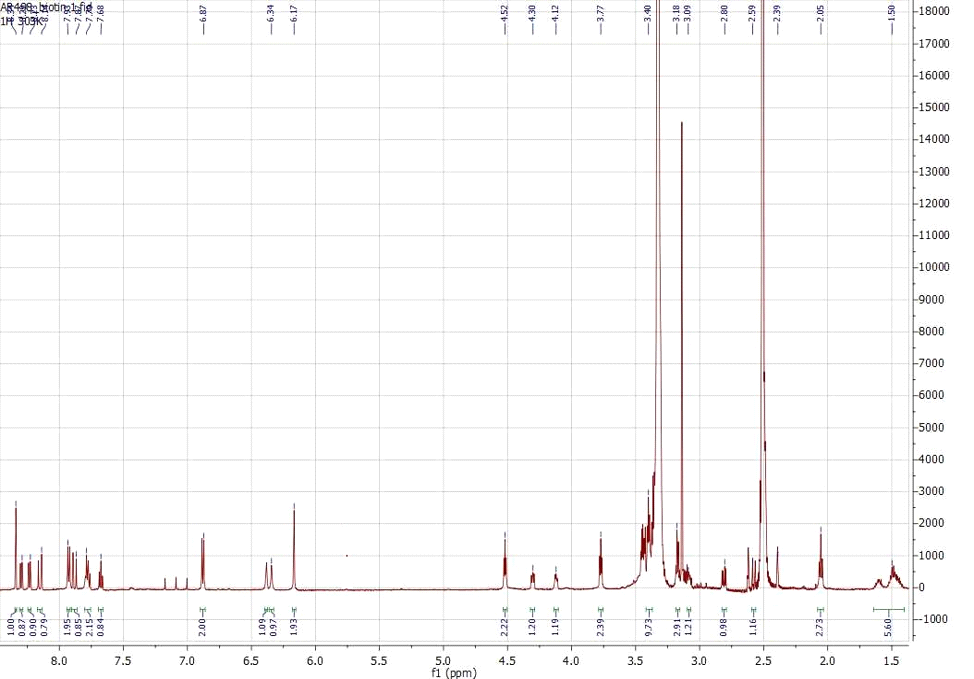


An expansion of the 2D ^1^H COSY NMR spectrum (aromatic region)


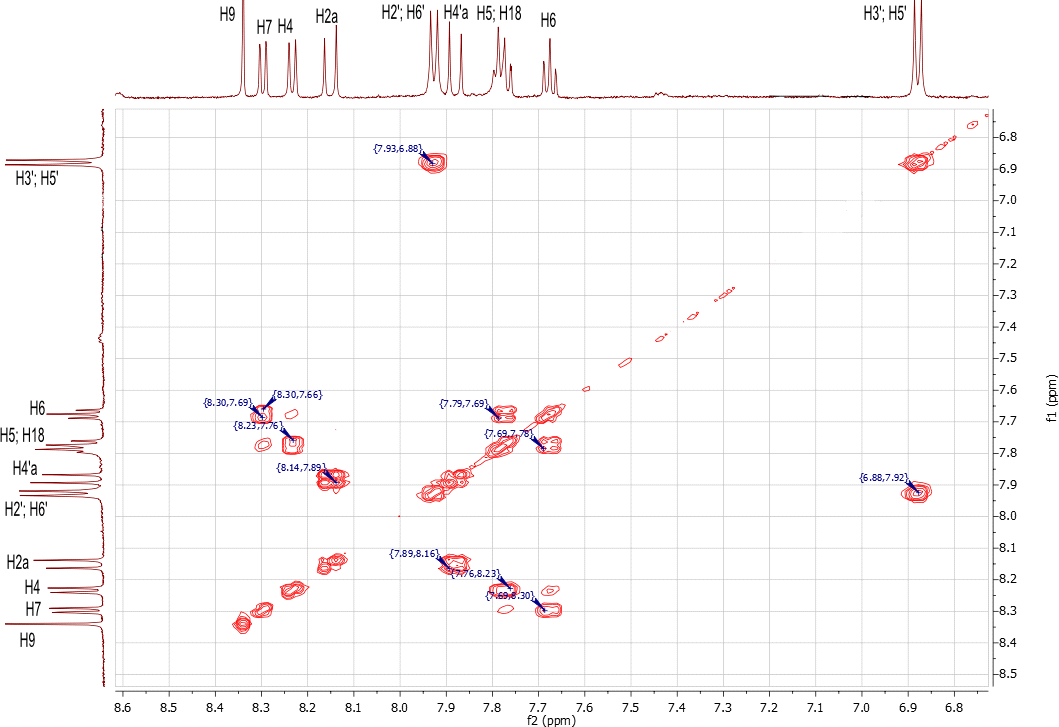


An expansion of the 2D ^1^H COSY NMR spectrum (aliphatic region)


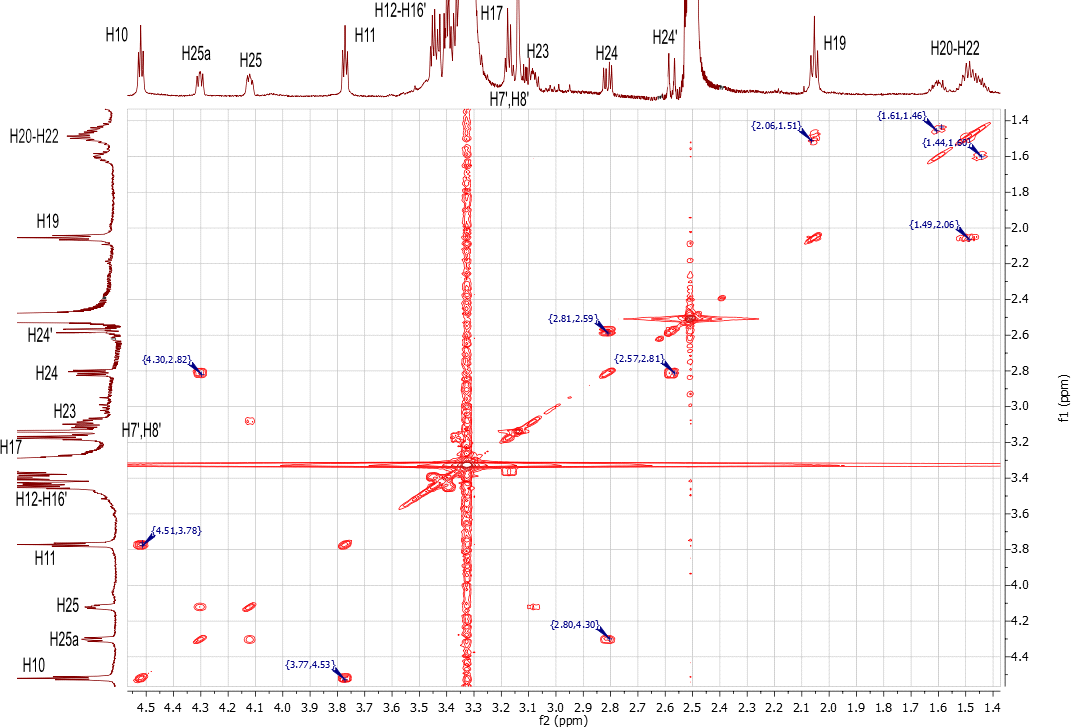


*3-((1-(13-oxo-17-(2-oxohexahydro-1H-thieno[3,4-d]imidazol-4-yl)-3,6,9-trioxa-12-azaheptadecyl)-1H-1,2,3-triazol-4-yl)methyl)-2-(2-(2,3,6,7-tetrahydro-1H,5H-pyrido[3,2,1-ij]quinolin-9-yl)vinyl)benzo[d]thiazol-3-ium 2,2,2-trifluoroacetate* **4c**


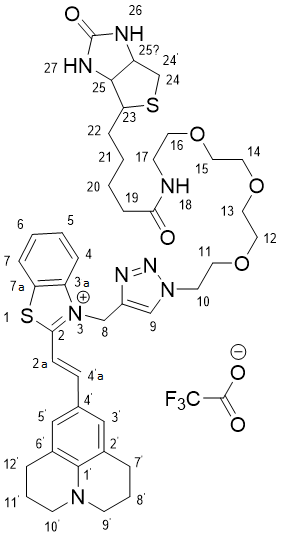


^1^H spectrum


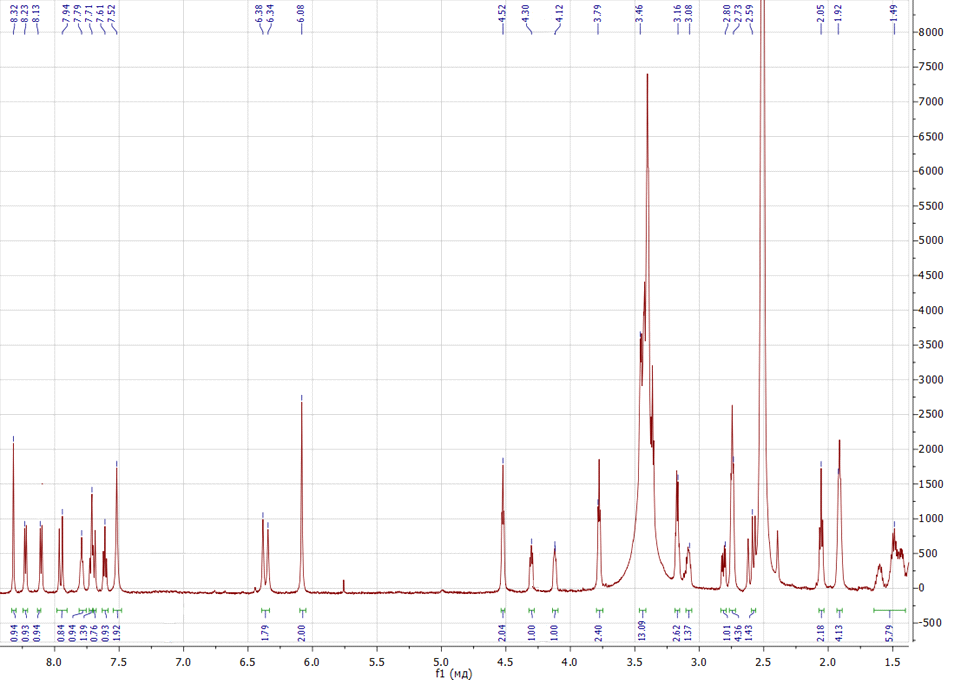


An expansion of the 2D ^1^H COSY NMR spectrum (aromatic region)


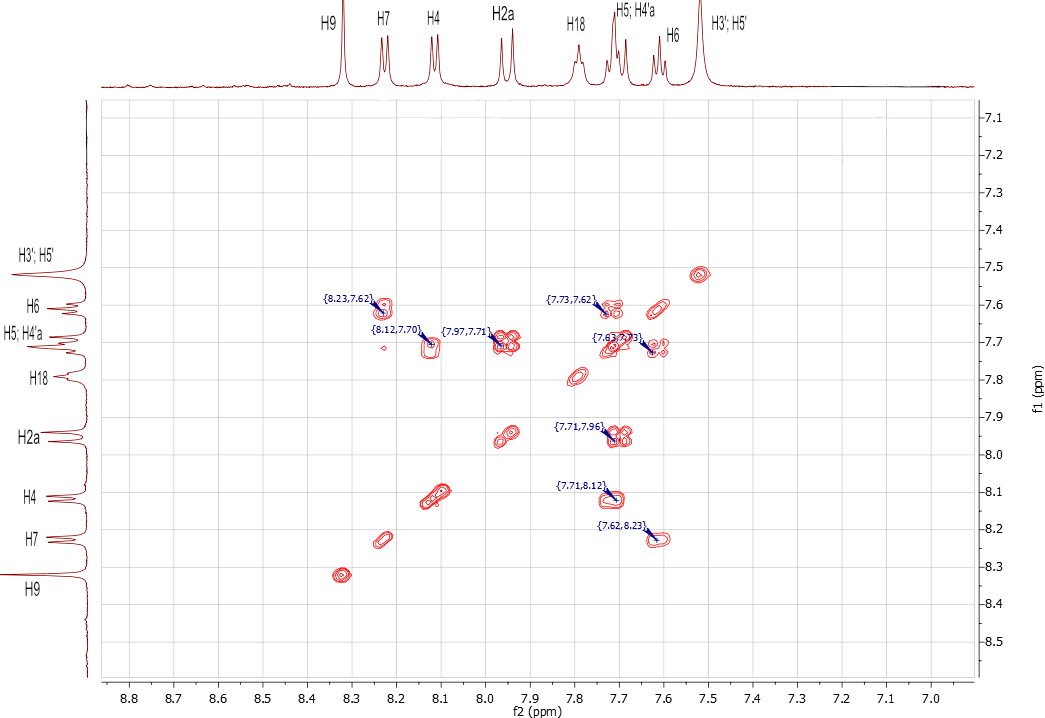


An expansion of the 2D ^1^H COSY NMR spectrum (aliphatic region)


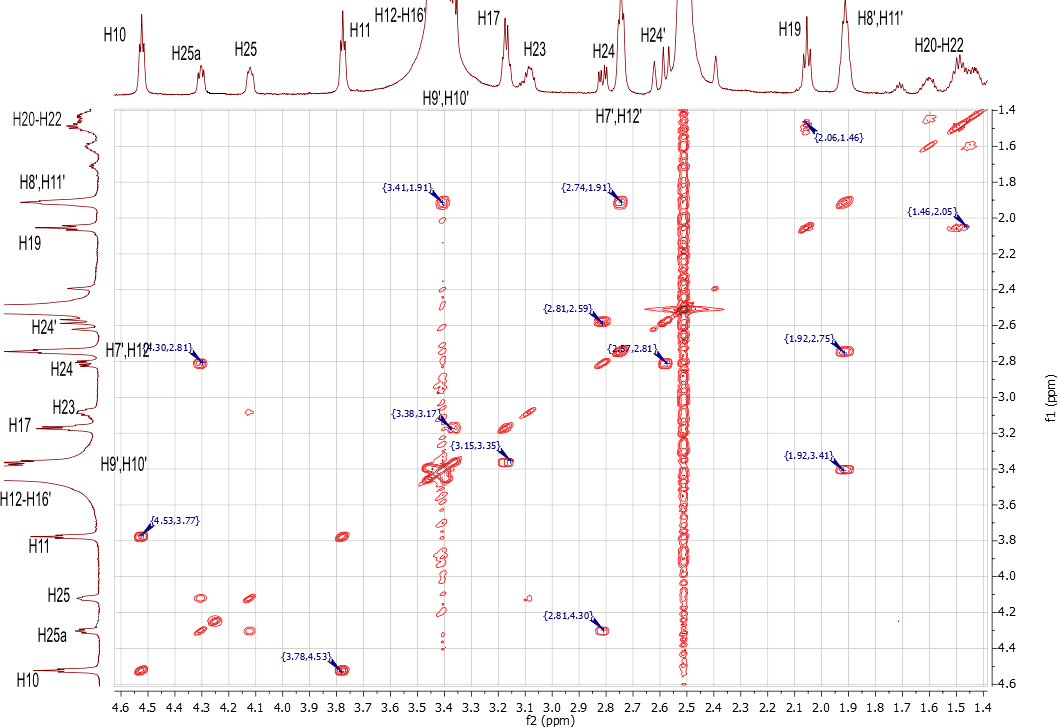


*3-((1-(13-oxo-17-(2-oxohexahydro-1H-thieno[3,4-d]imidazol-4-yl)-3,6,9-trioxa-12-azaheptadecyl)-1H-1,2,3-triazol-4-yl)methyl)-2-(2-(1,2,2,4-tetramethyl-1,2-dihydroquinolin-6-yl)vinyl)benzo[d]thiazol-3-ium 2,2,2-trifluoroacetate* **4d**


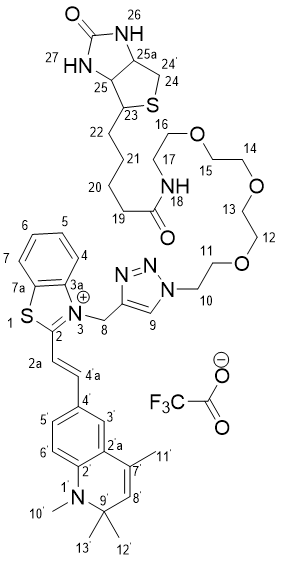


^1^H spectrum


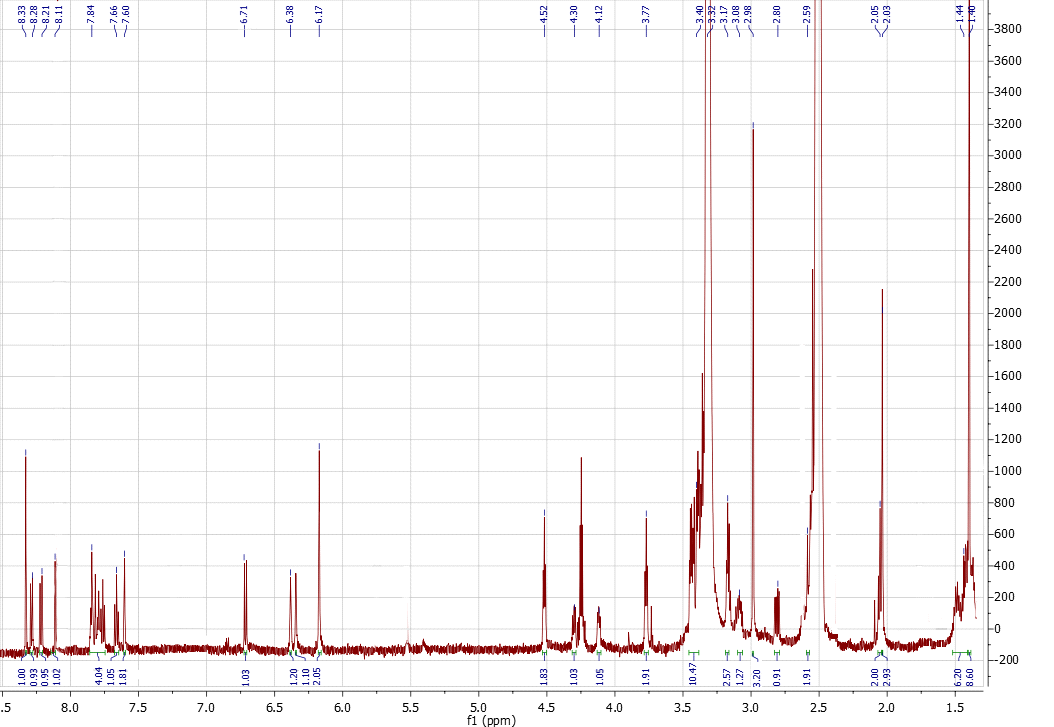


An expansion of the 2D ^1^H COSY NMR spectrum (aromatic region)


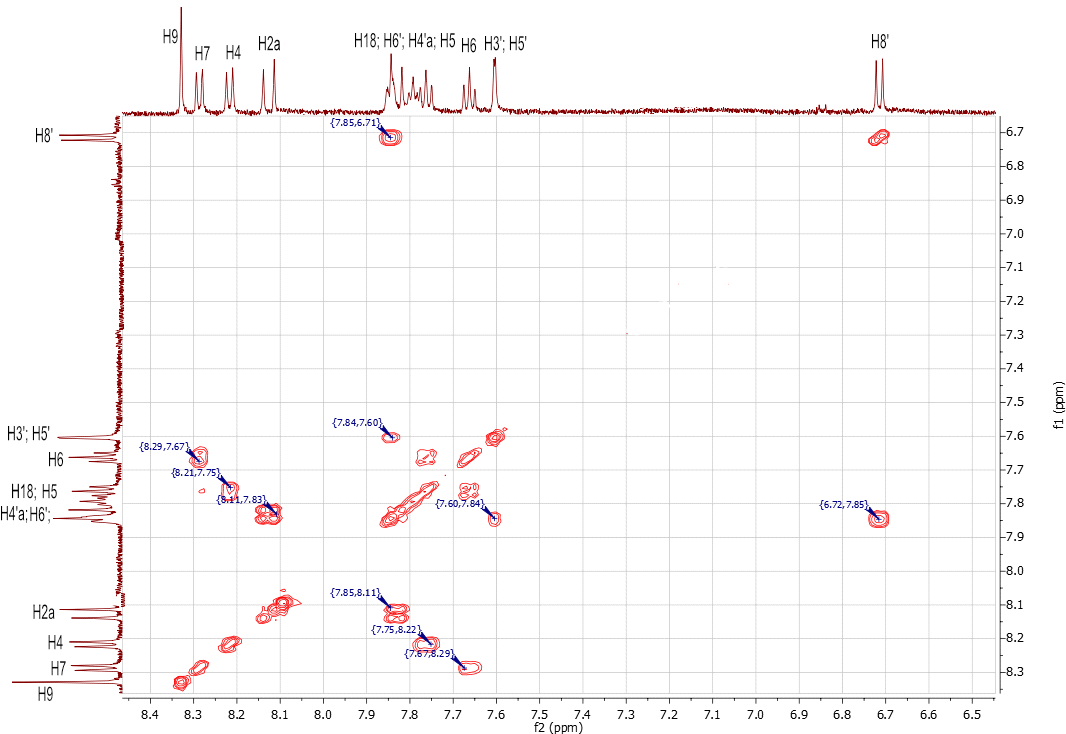


An expansion of the 2D ^1^H COSY NMR spectrum (aliphatic region)


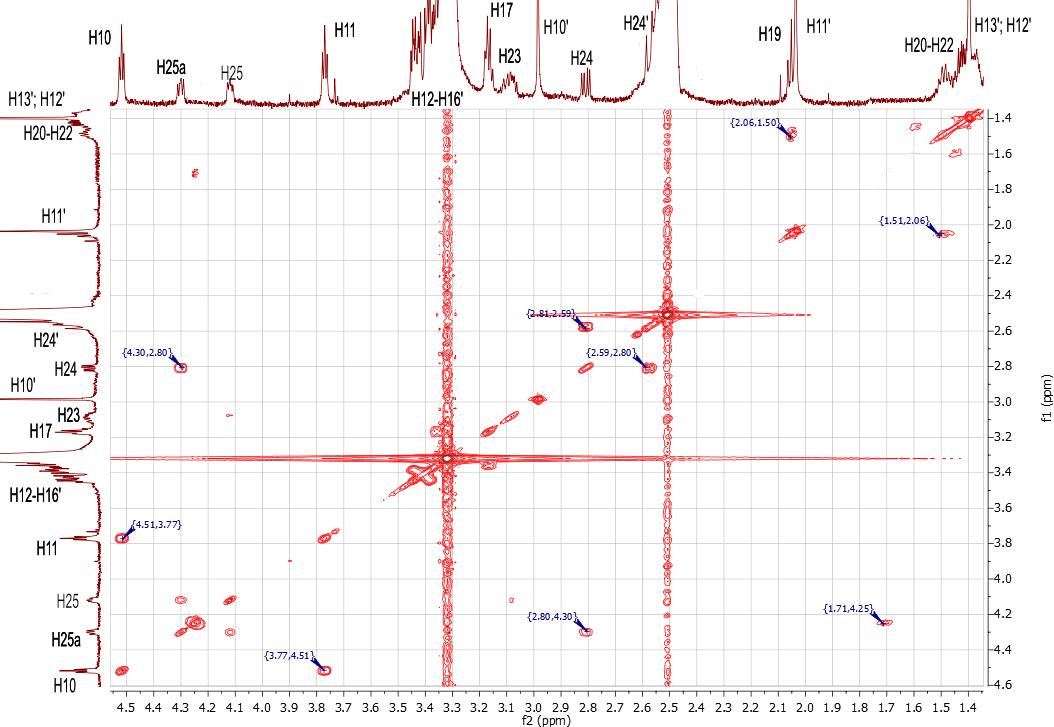


# HPLC and MS data

*(E)-2-((1-methylquinolin-4(1H)-ylidene)methyl)-3-(prop-2-yn-1-yl)benzo[d]thiazol-3-ium bromide* **2a**


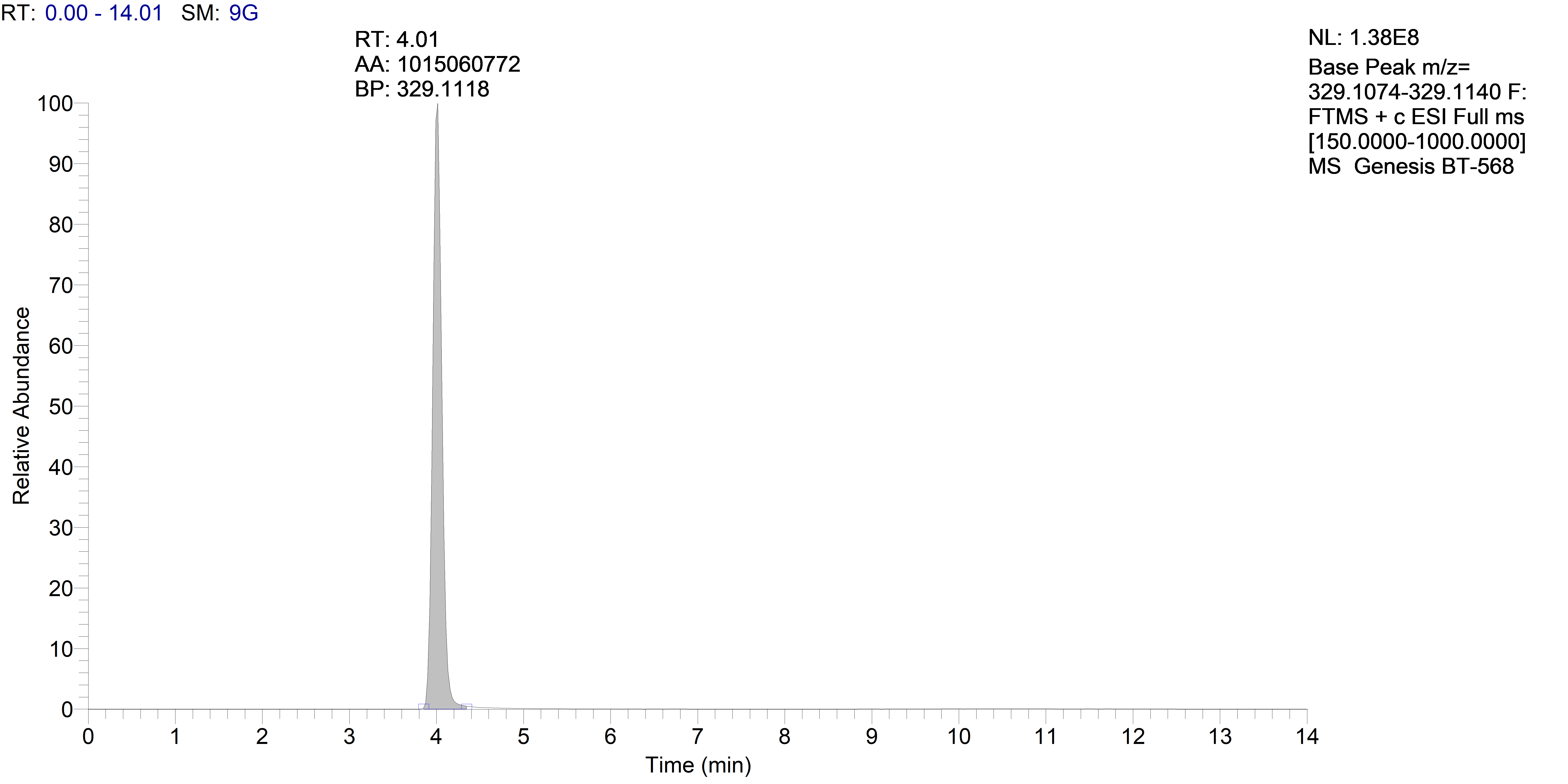


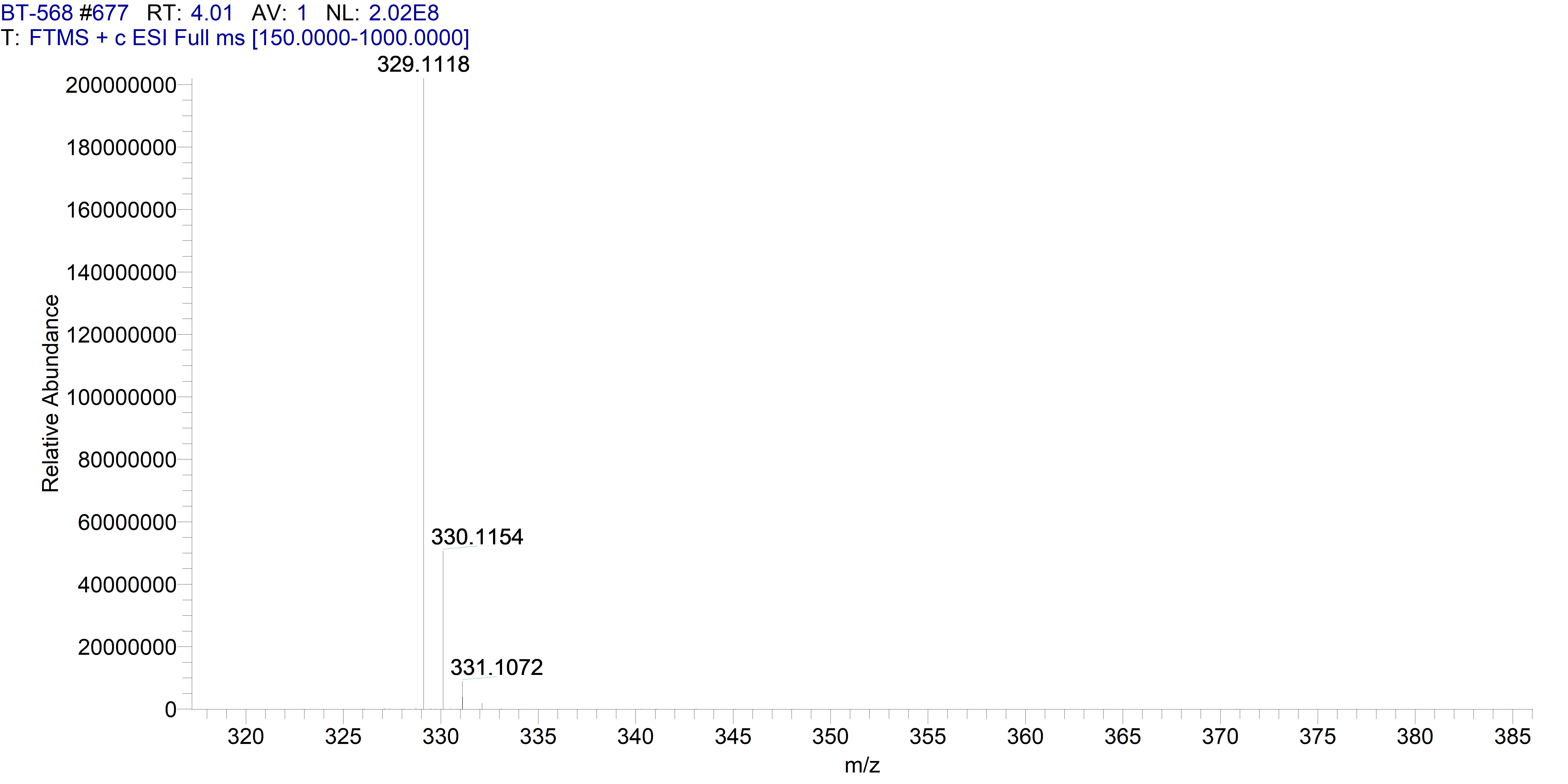


*(E)-2-(2-(4-(Dimethylamino)phenyl)ethenyl)-3-(2-propynyl)benzo[d]thiazolium bromide* **2b**


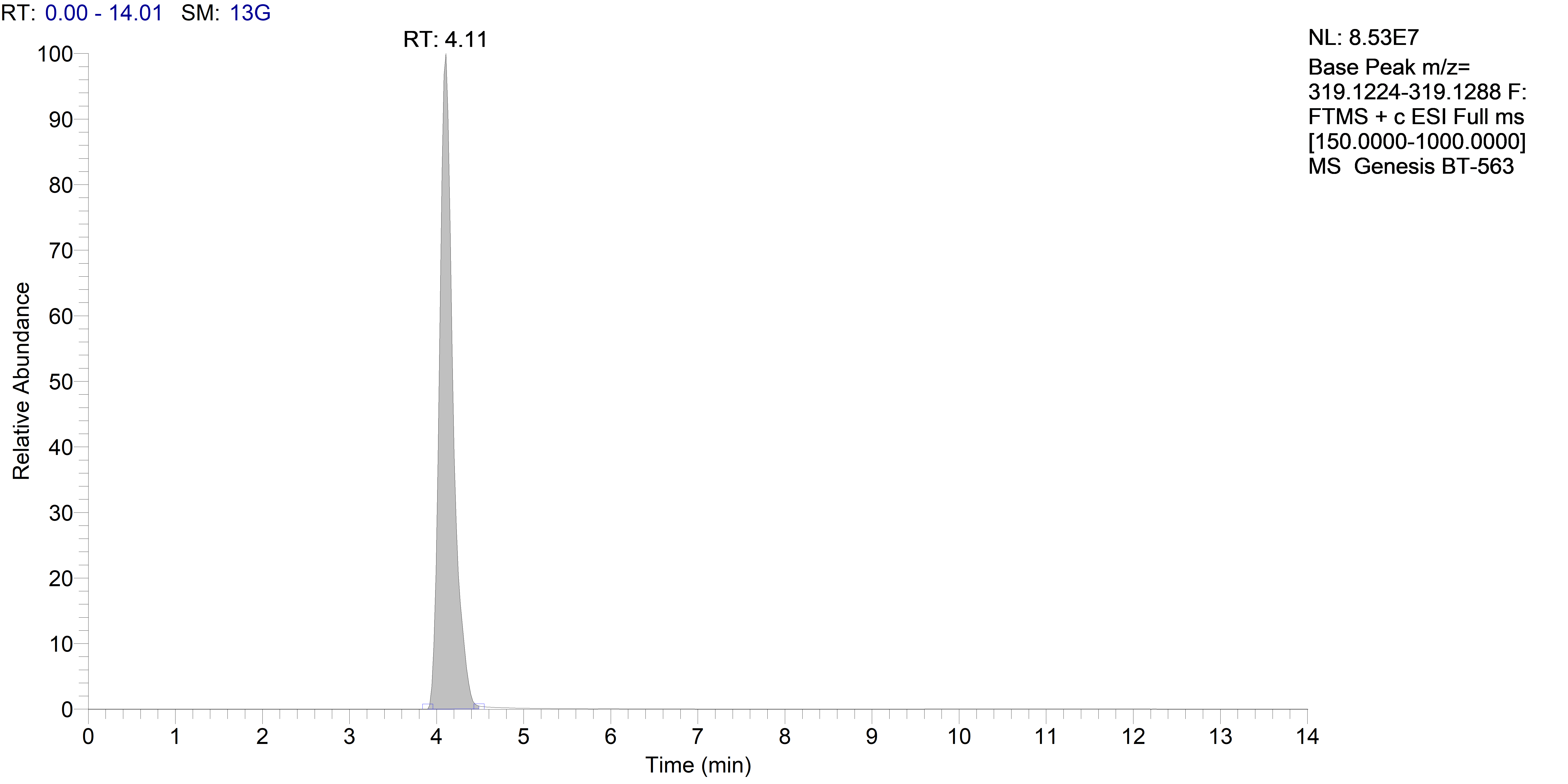


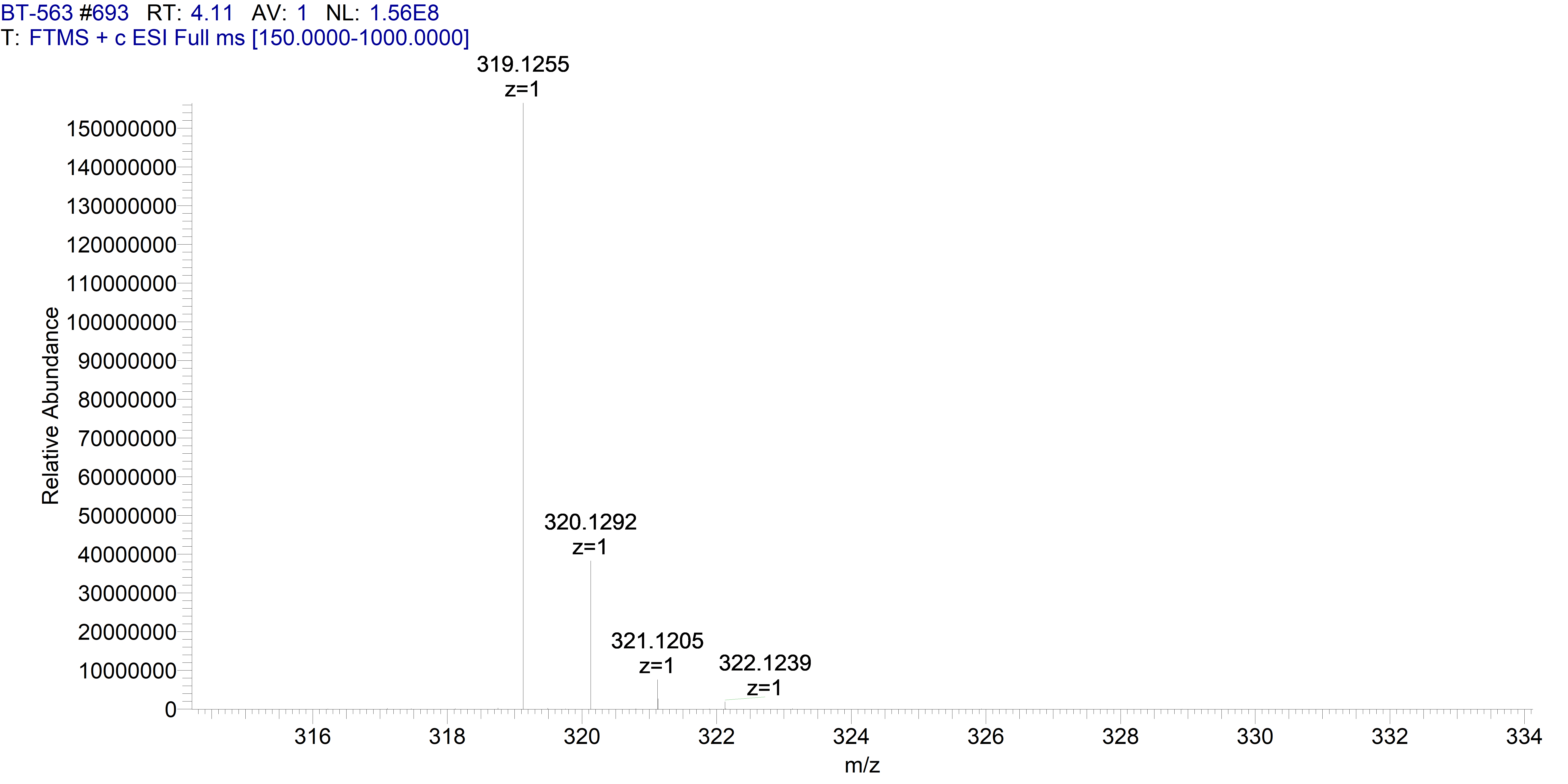


*(E)-3-(prop-2-yn-1-yl)-2-(2-(2,3,6,7-tetrahydro-1H,5H-pyrido[3,2,1-ij]quinolin-9-yl)vinyl)benzo[d]thiazol-3-ium bromide* **2c**


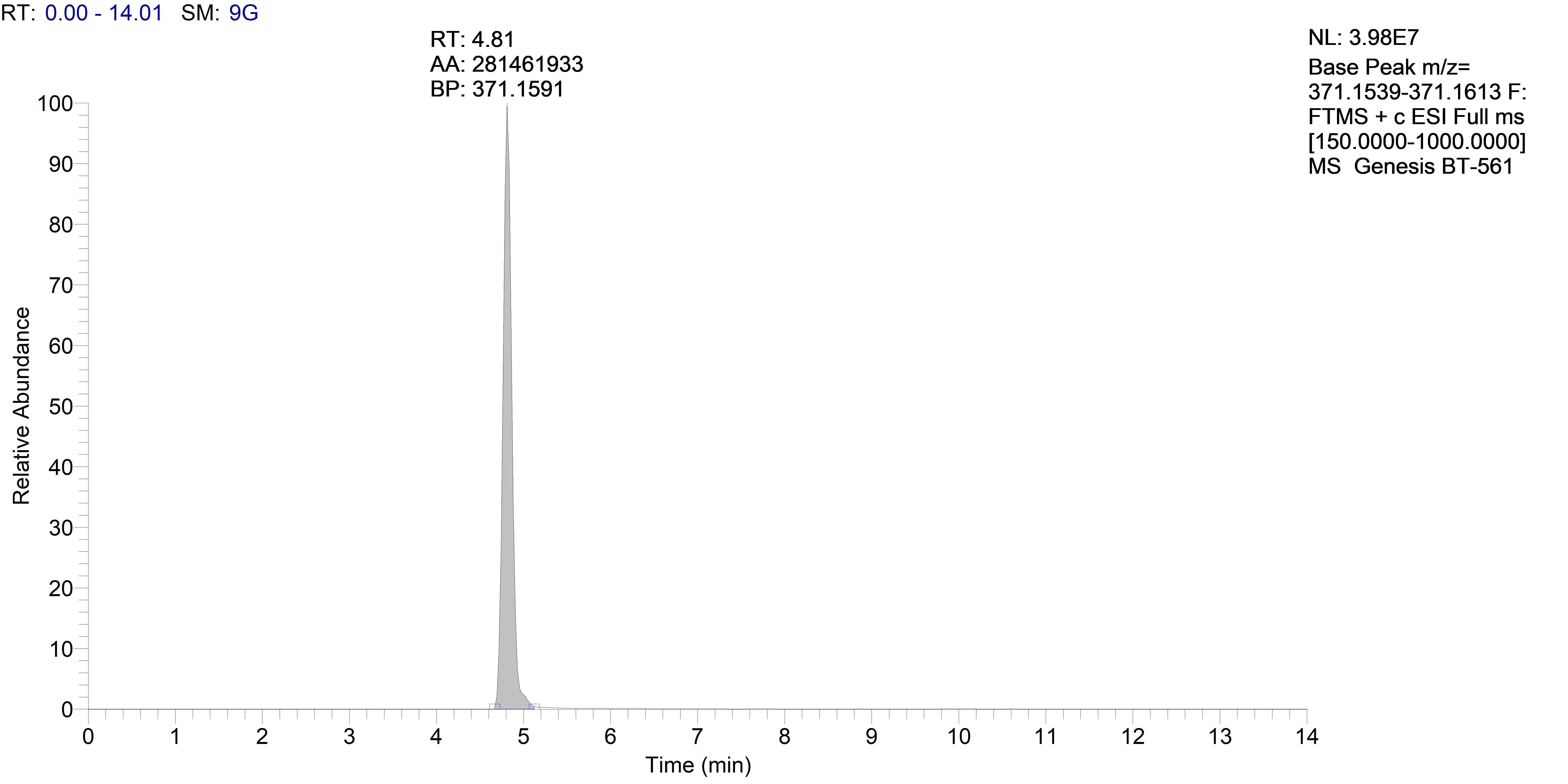


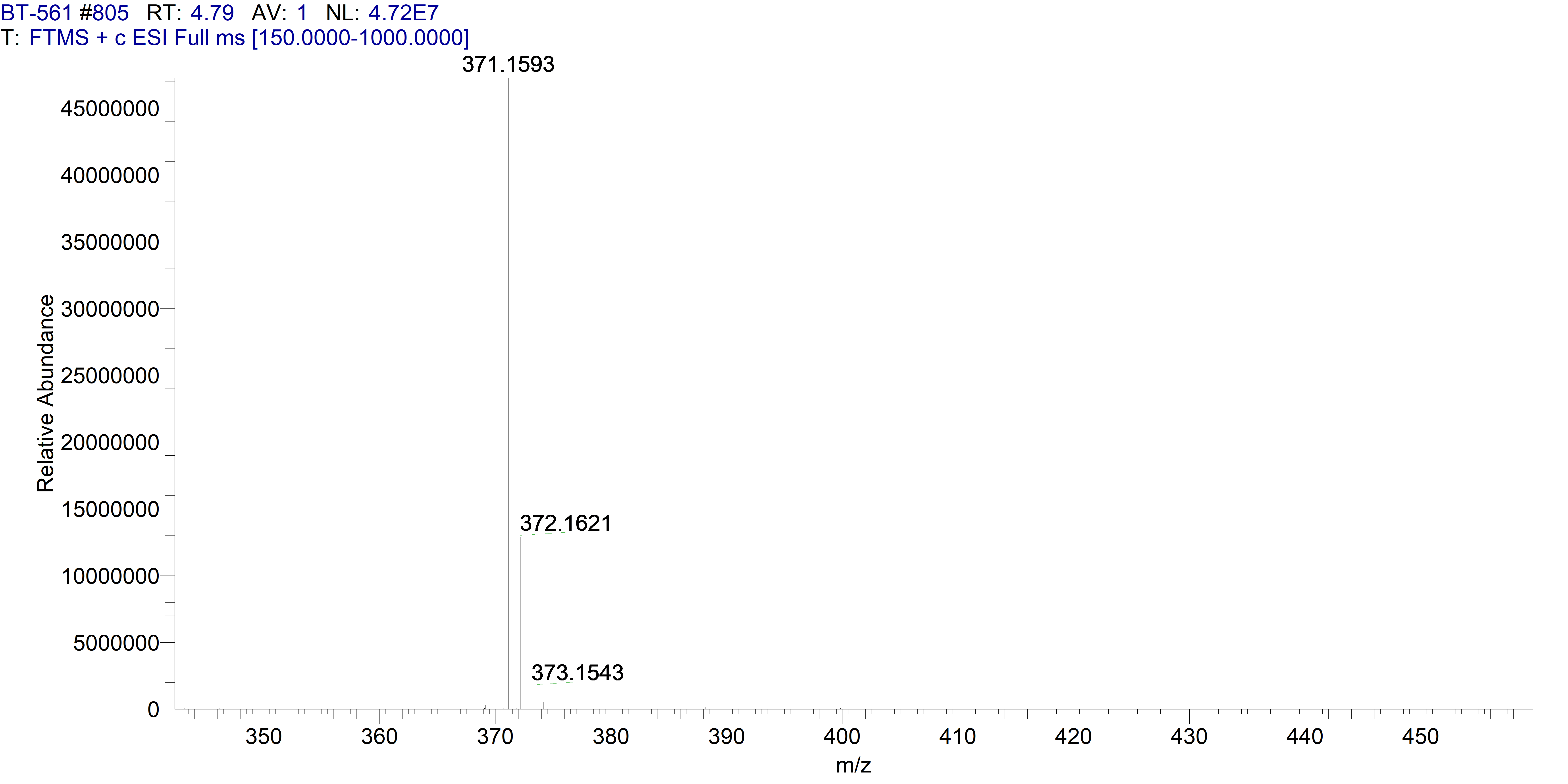


*(E)-3-(prop-2-yn-1-yl)-2-(2-(1,2,2,4-tetramethyl-1,2-dihydroquinolin-6-yl)vinyl)benzo[d]thiazol-3-ium bromide* **2d**


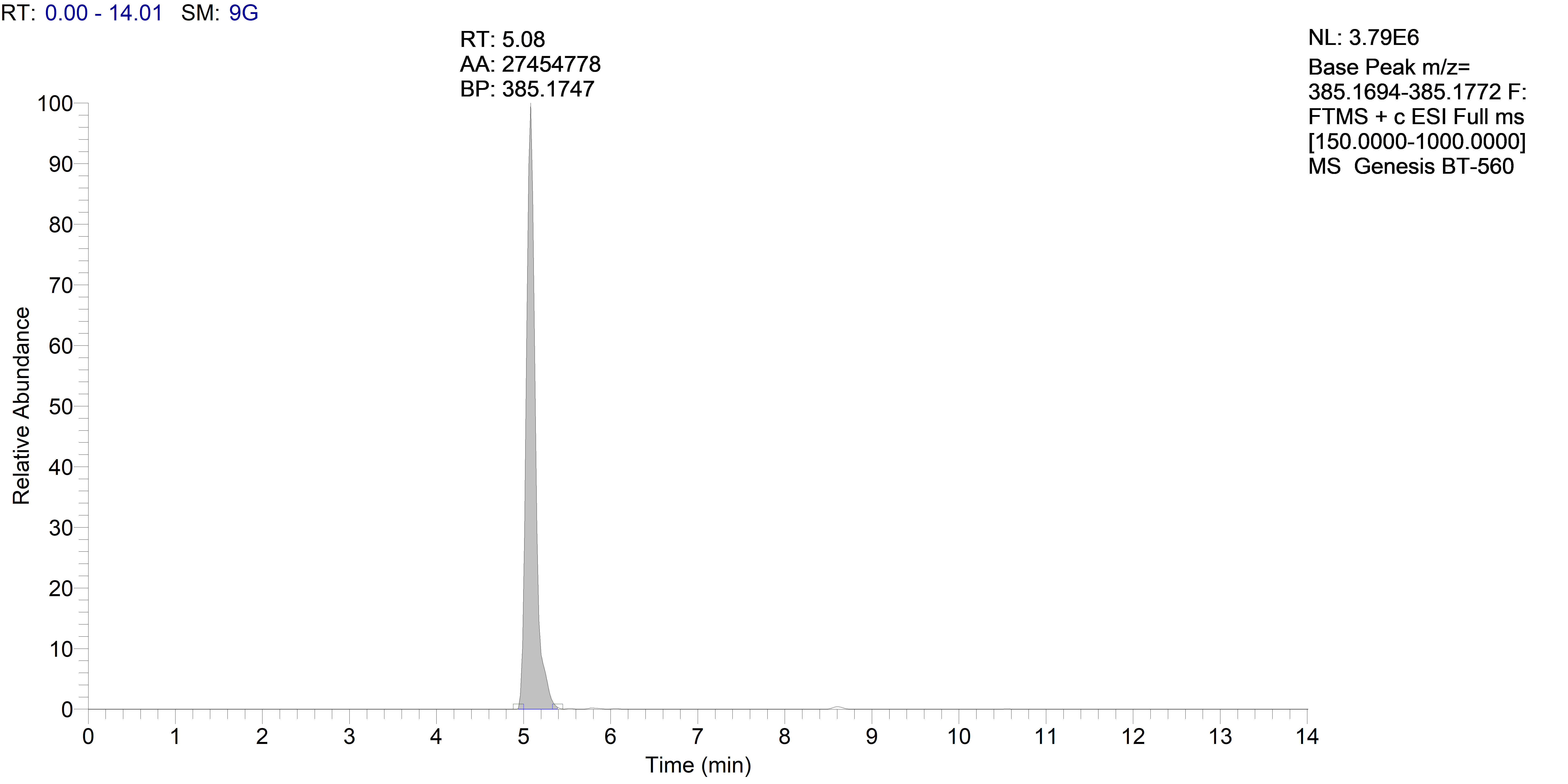


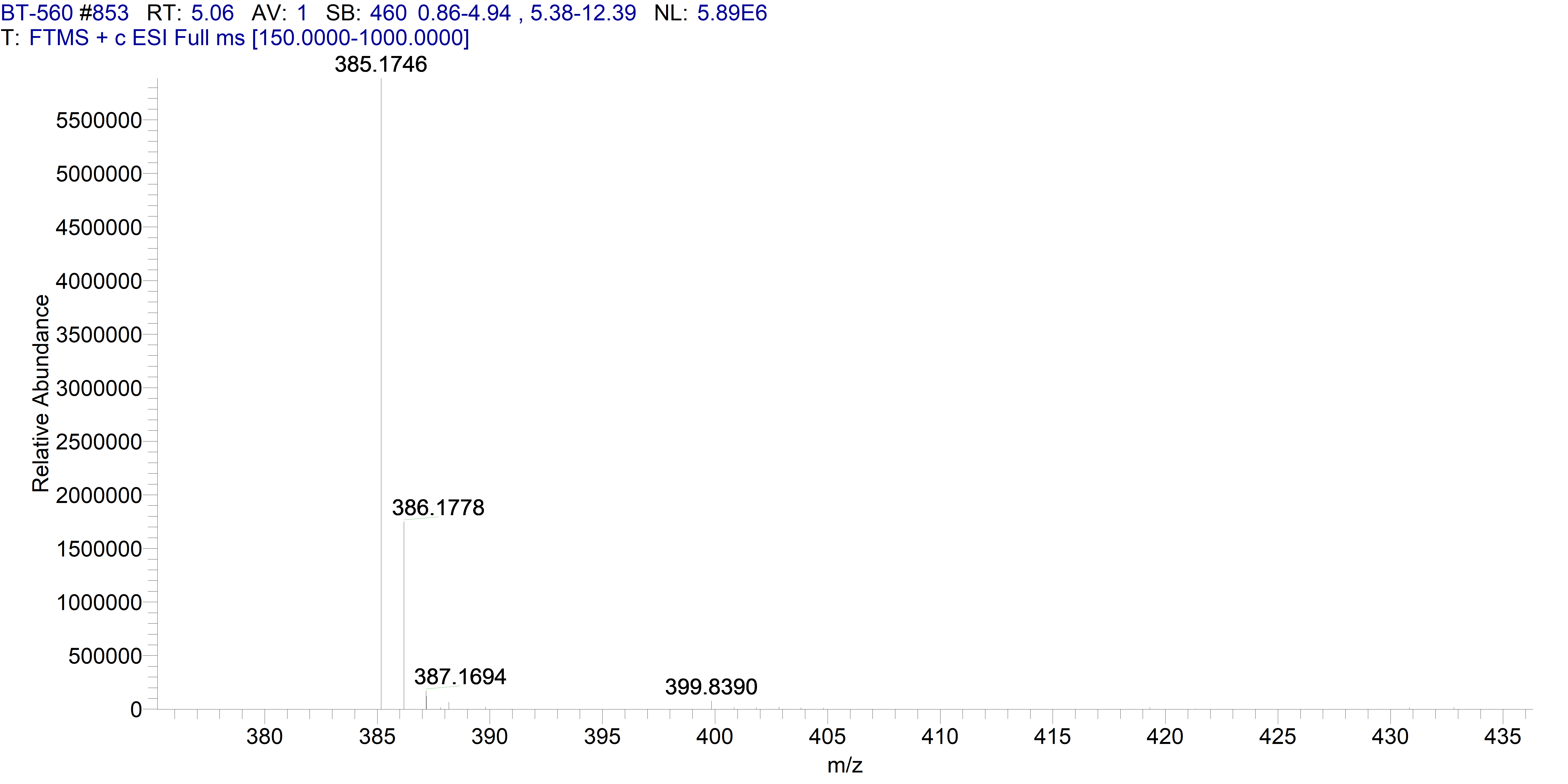


**TO-1 biotin**


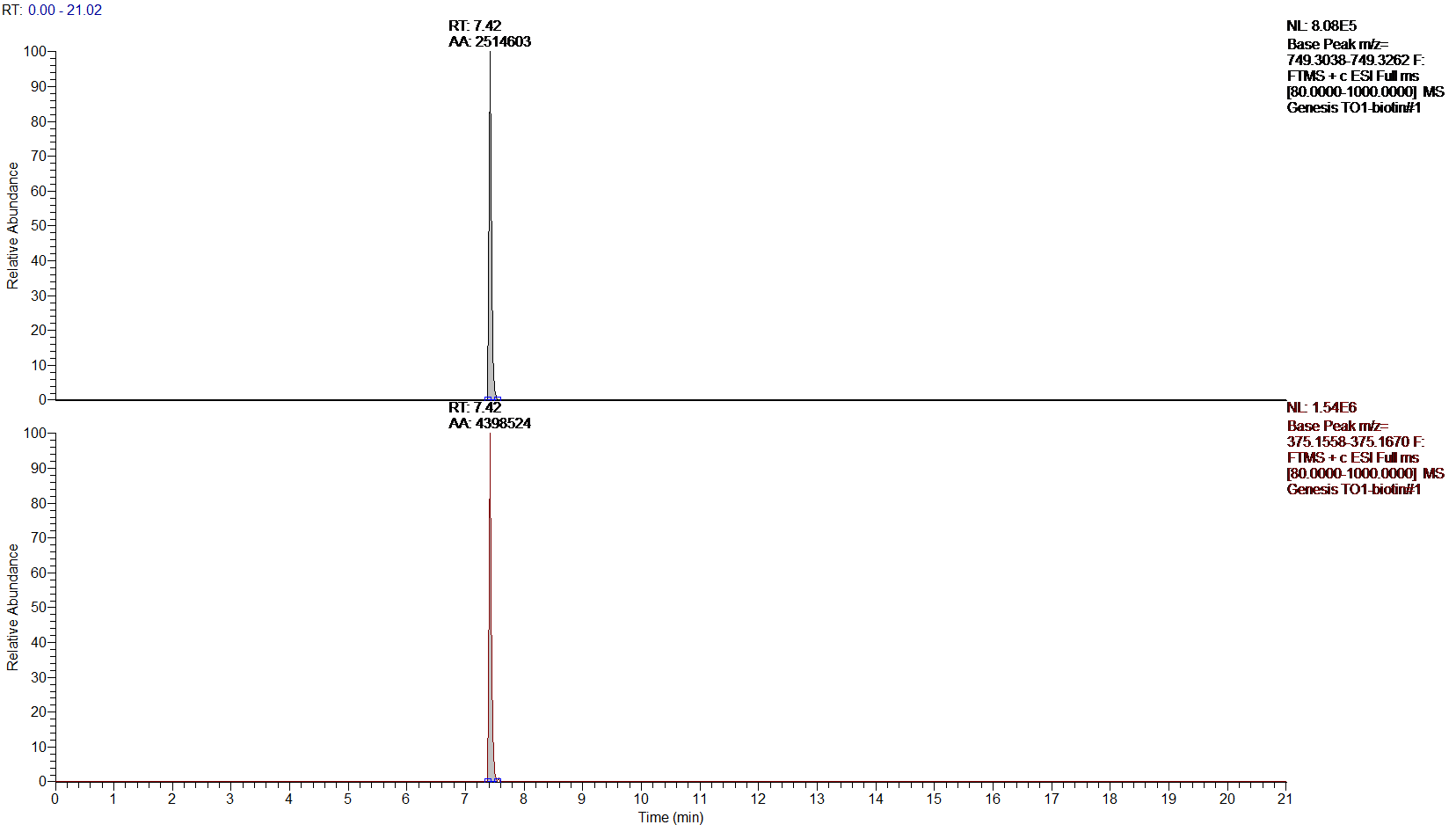


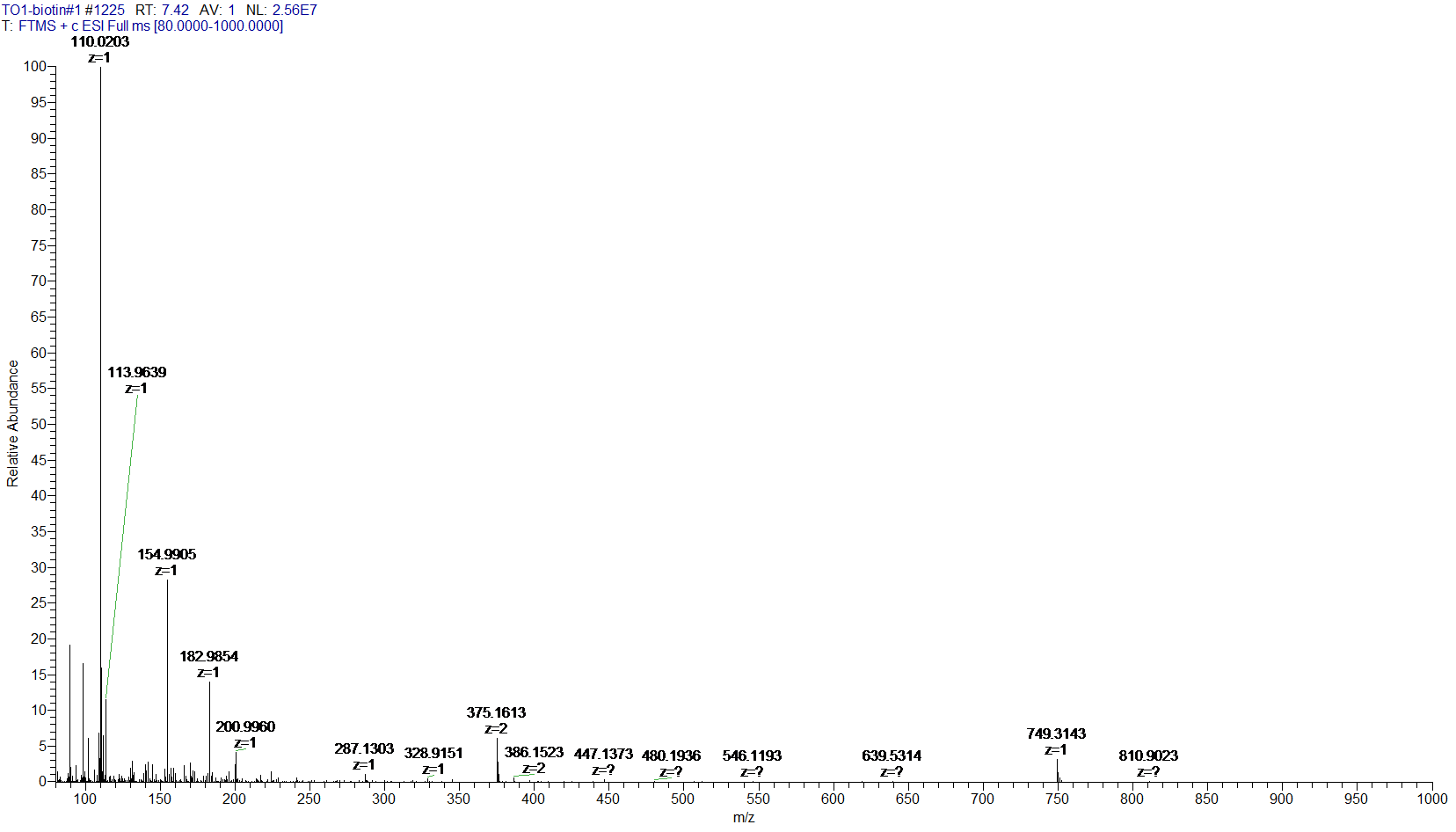


*2-((1-methylquinolin-4(1H)-ylidene)methyl)-3-((1-(13-oxo-17-(2-oxohexahydro-1H-thieno[3,4-d]imidazol-4-yl)-3,6,9-trioxa-12-azaheptadecyl)-1H-1,2,3-triazol-4-yl)methyl)benzo[d]thiazol-3-ium 2,2,2-trifluoroacetate* **4a**


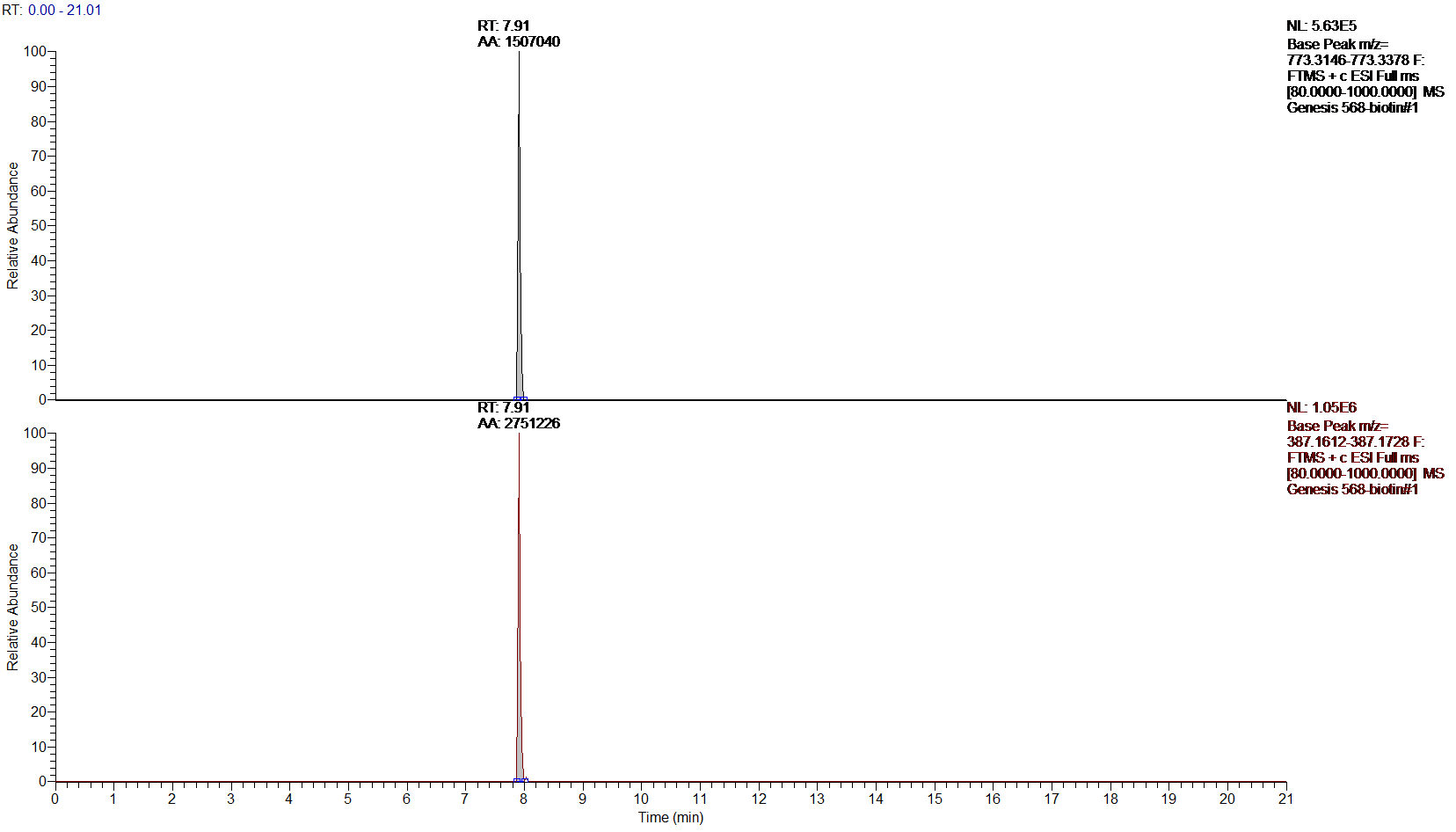


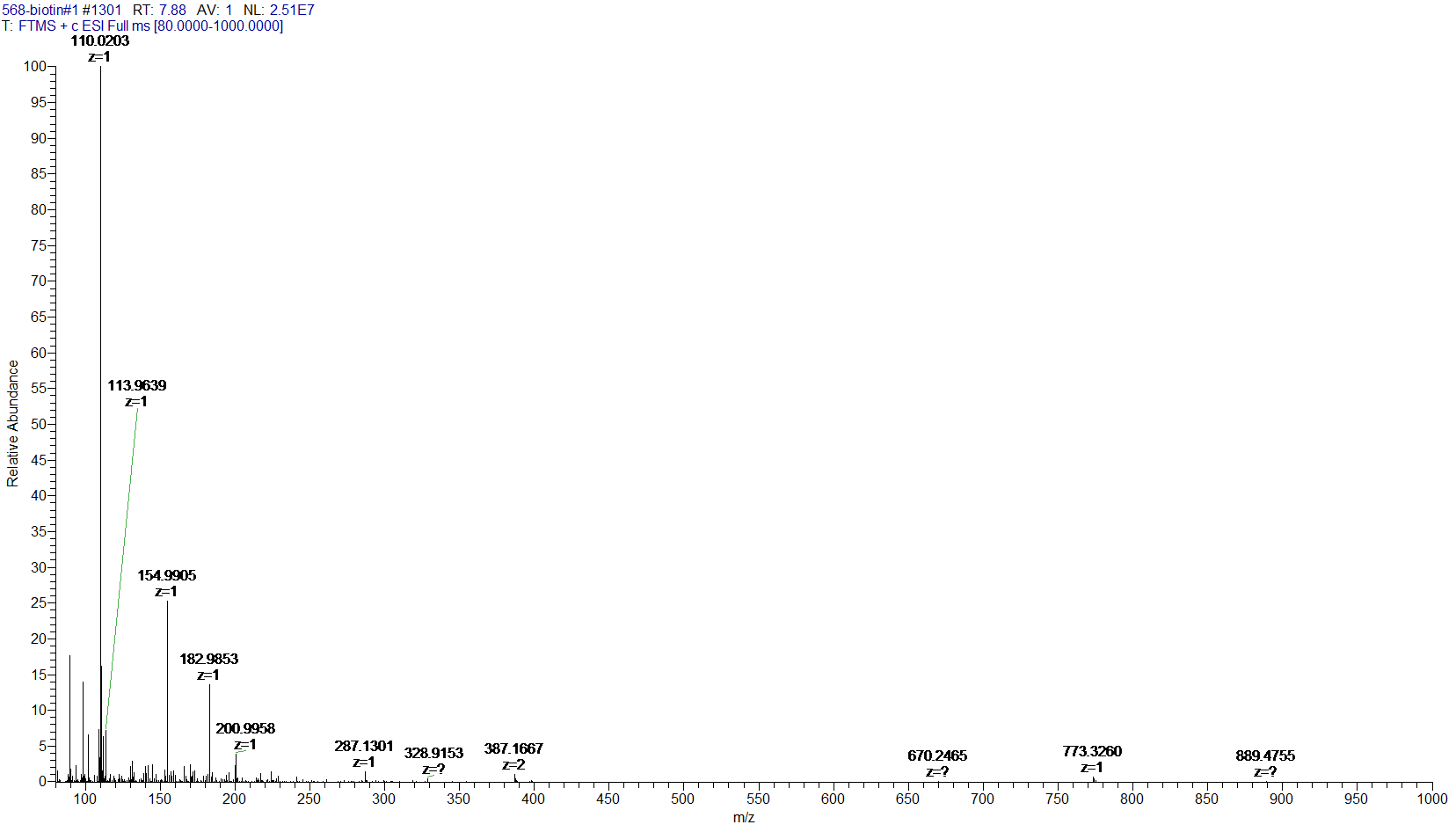


*2-(4-(dimethylamino)styryl)-3-((1-(13-oxo-17-(2-oxohexahydro-1H-thieno[3,4-d]imidazol-4-yl)-3,6,9-trioxa-12-azaheptadecyl)-1H-1,2,3-triazol-4-yl)methyl)benzo[d]thiazol-3-ium 2,2,2-trifluoroacetate* **4b**


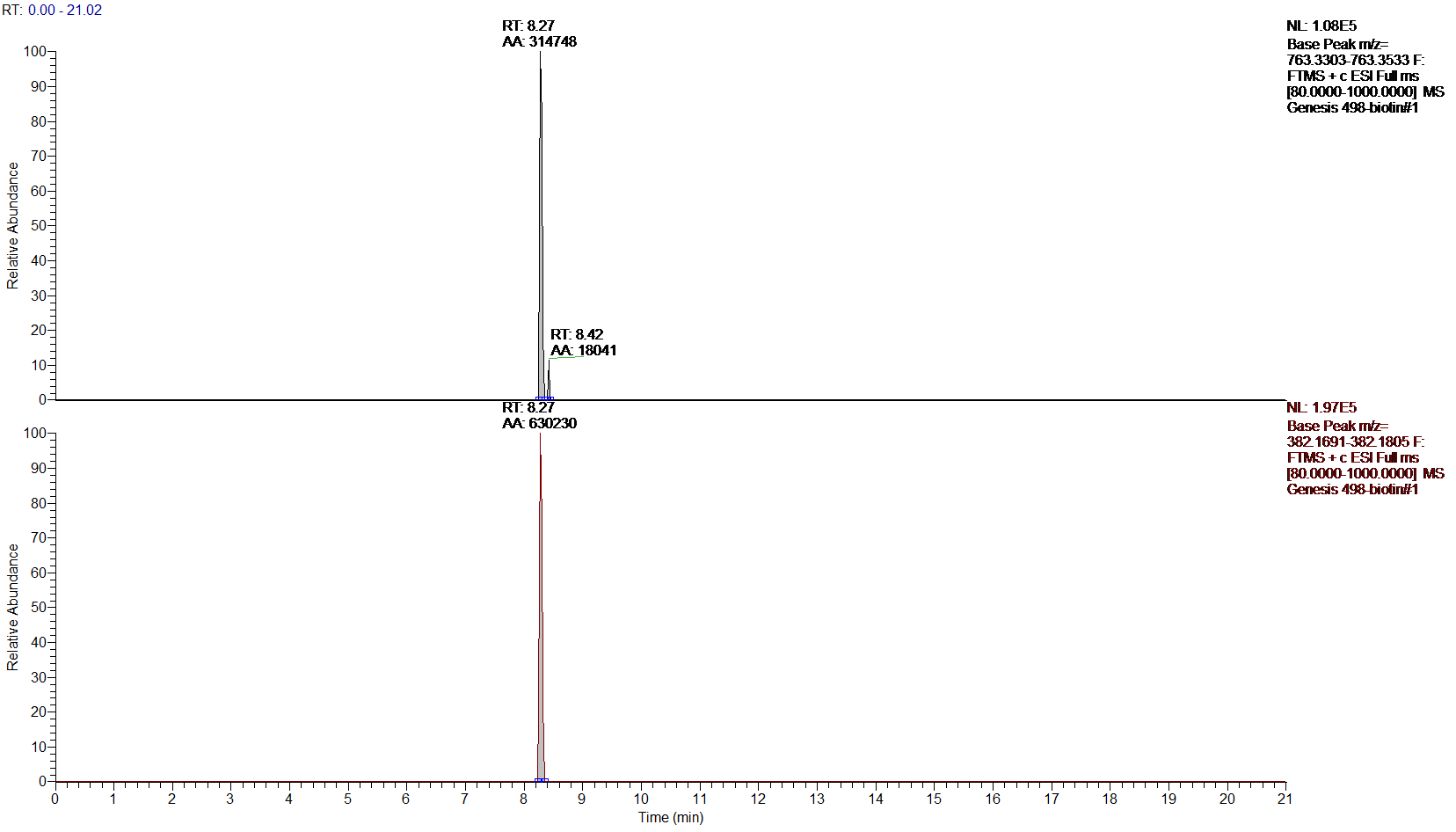


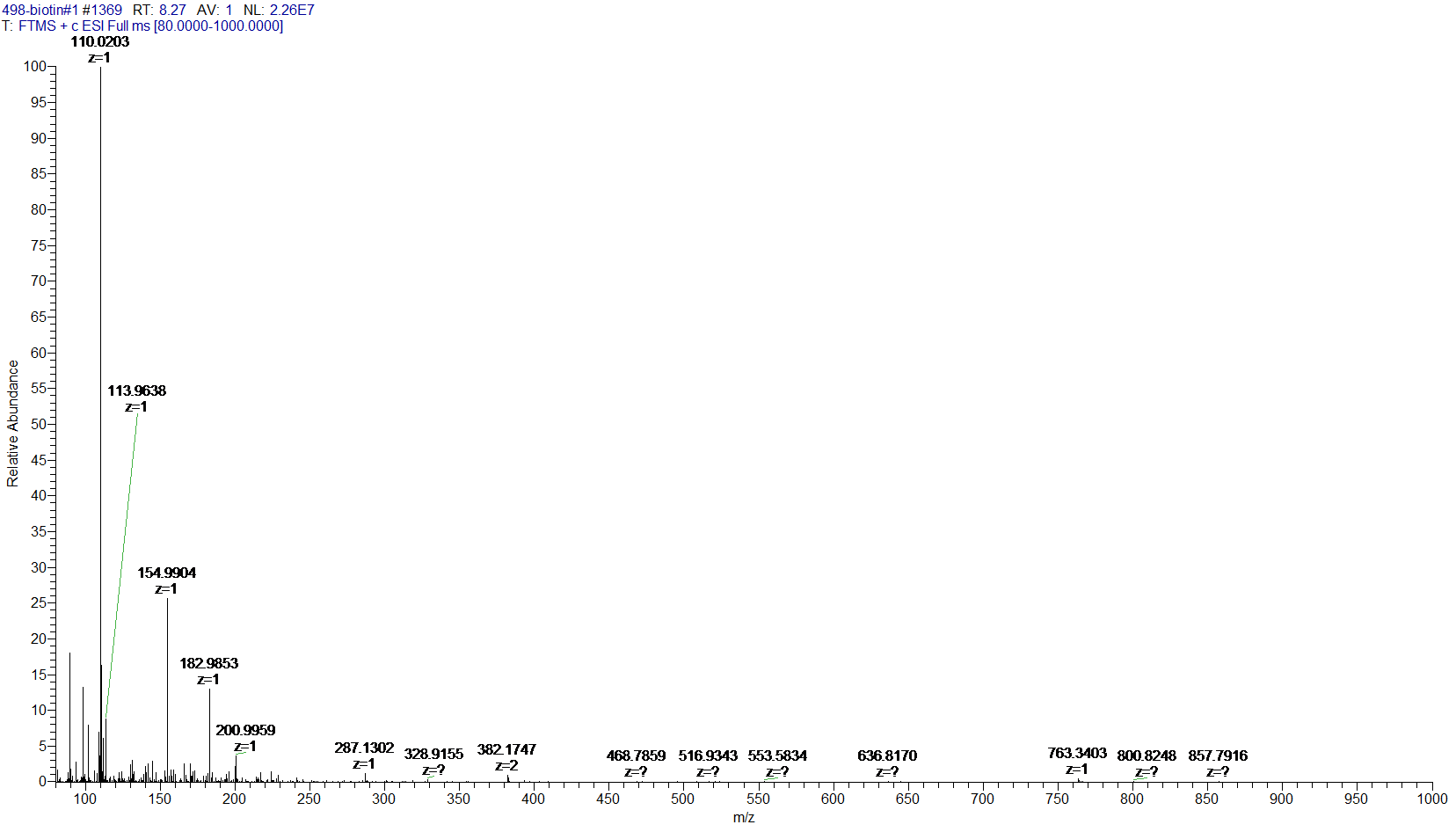


*3-((1-(13-oxo-17-(2-oxohexahydro-1H-thieno[3,4-d]imidazol-4-yl)-3,6,9-trioxa-12-azaheptadecyl)-1H-1,2,3-triazol-4-yl)methyl)-2-(2-(2,3,6,7-tetrahydro-1H,5H-pyrido[3,2,1-ij]quinolin-9-yl)vinyl)benzo[d]thiazol-3-ium 2,2,2-trifluoroacetate* **4c**


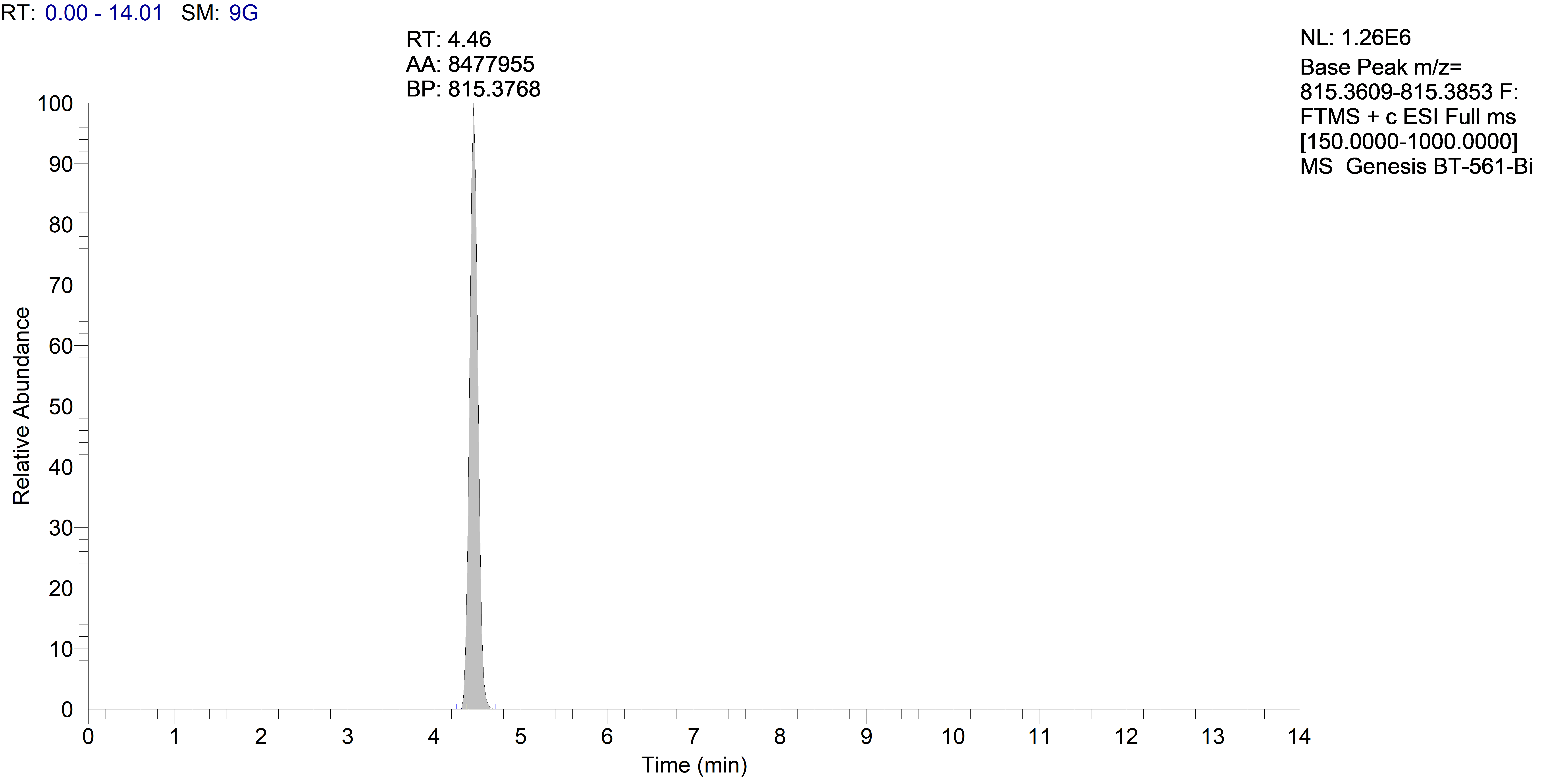


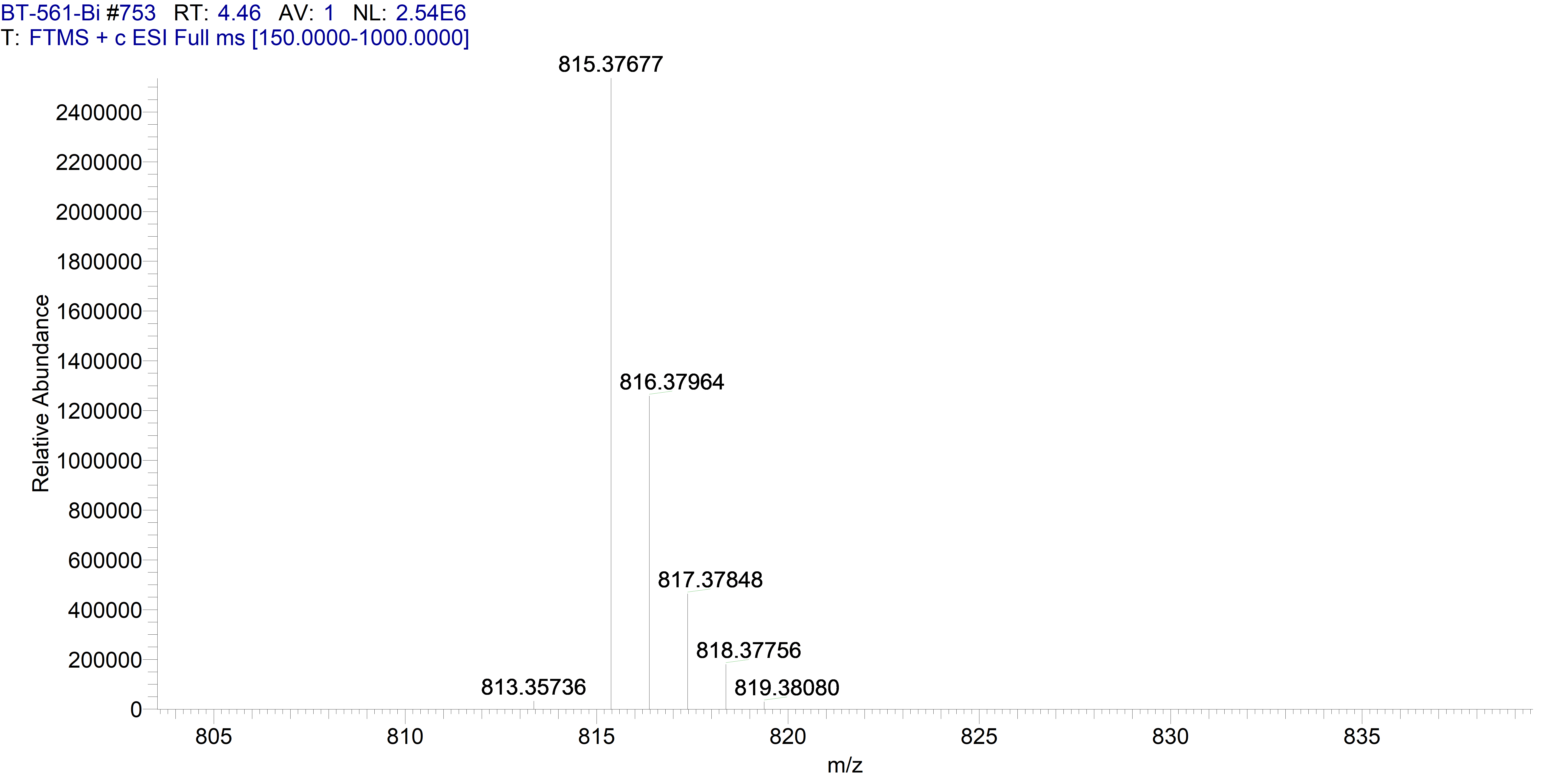


*3-((1-(13-oxo-17-(2-oxohexahydro-1H-thieno[3,4-d]imidazol-4-yl)-3,6,9-trioxa-12-azaheptadecyl)-1H-1,2,3-triazol-4-yl)methyl)-2-(2-(1,2,2,4-tetramethyl-1,2-dihydroquinolin-6-yl)vinyl)benzo[d]thiazol-3-ium 2,2,2-trifluoroacetate* **4d**


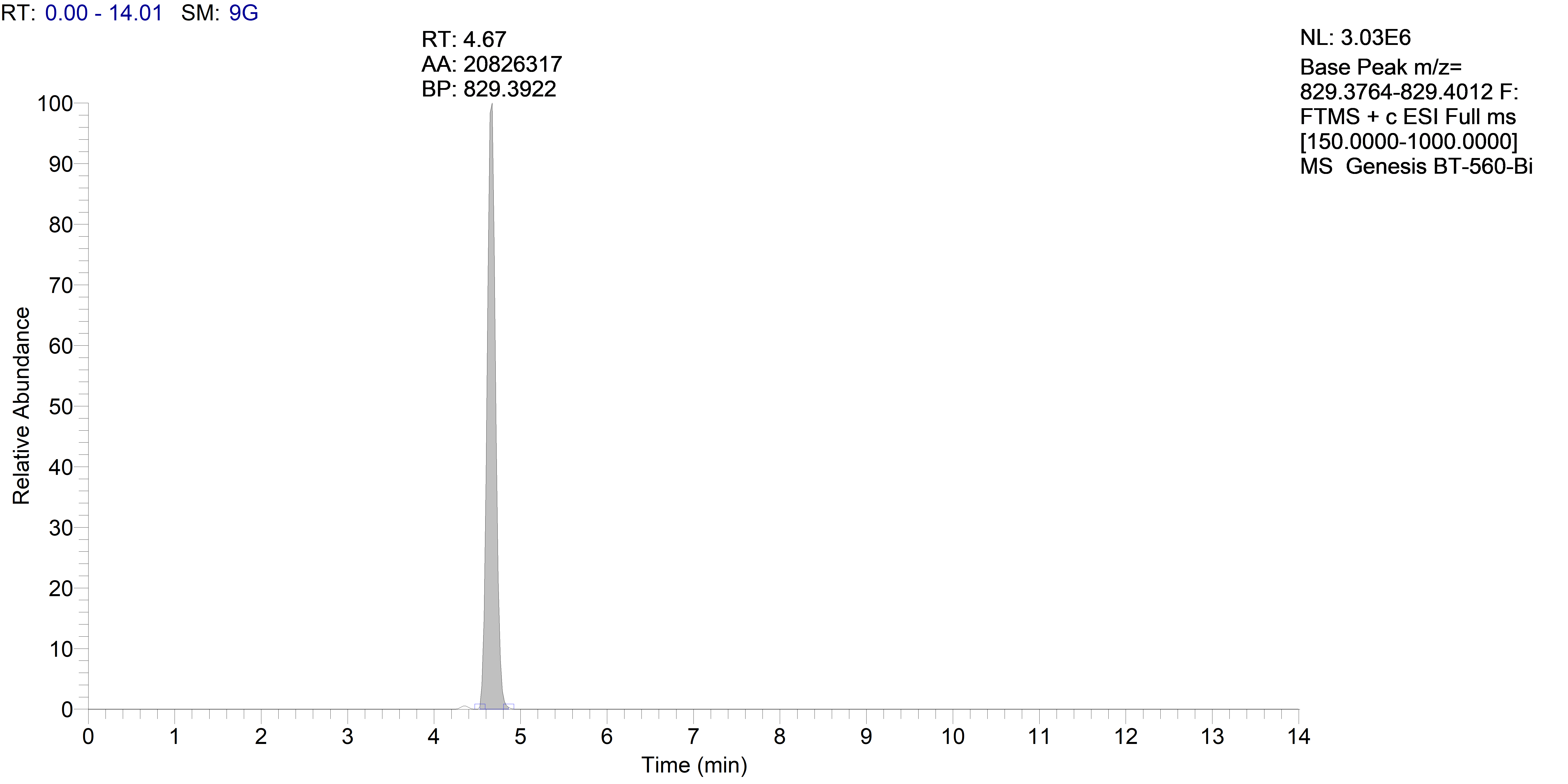


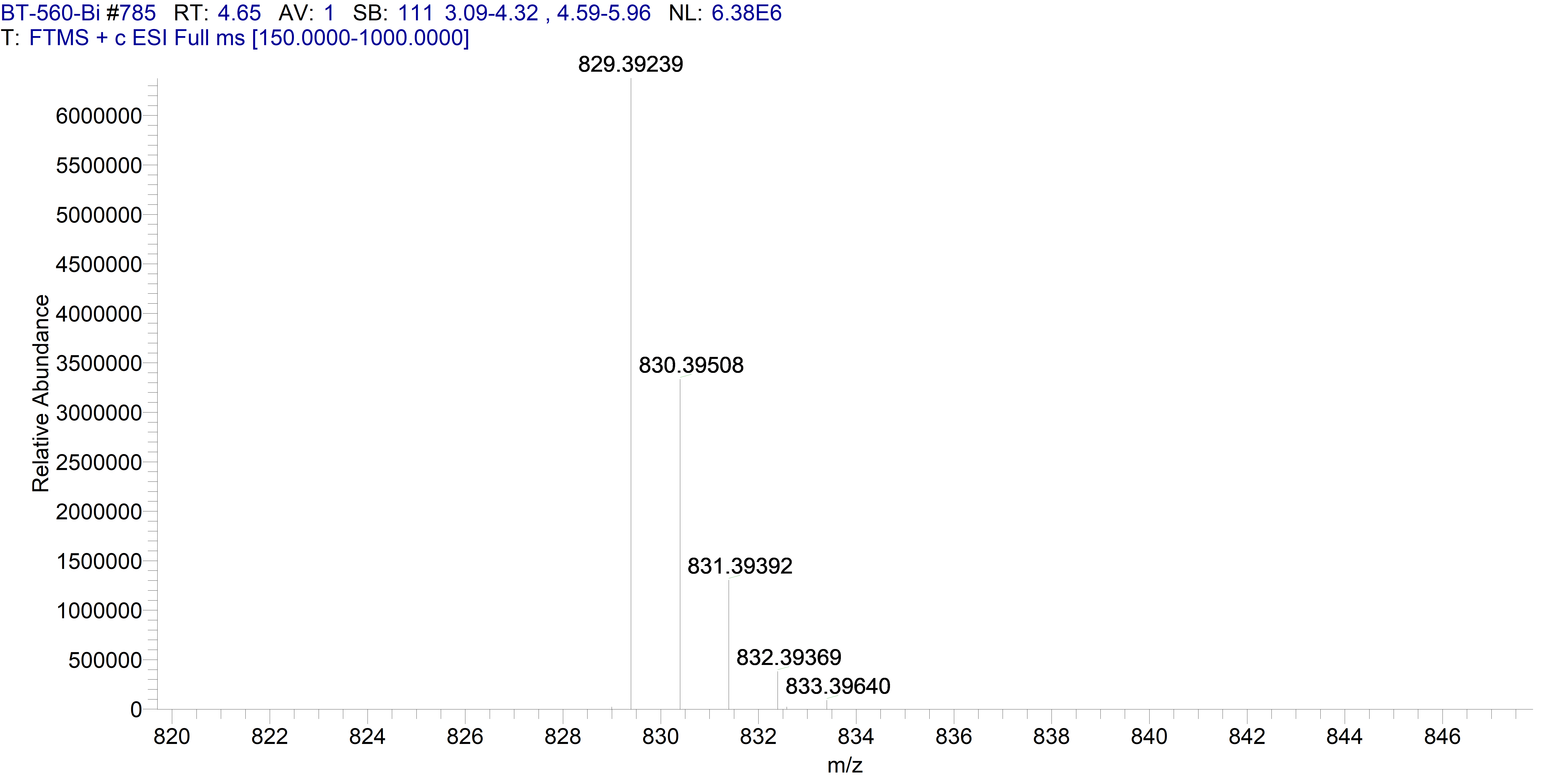


# References

1. Shen D*, et al.* Design, synthesis and evaluation of a novel fluorescent probe to accurately detect H2S in hepatocytes and natural waters. *Spectrochim Acta A Mol Biomol Spectrosc* **228**, 117690 (2020).
2. Ditmangklo B, Taechalertpaisarn J, Siriwong K, Vilaivan T. Clickable styryl dyes for fluorescence labeling of pyrrolidinyl PNA probes for the detection of base mutations in DNA. *Org Biomol Chem* **17**, 9712-9725 (2019).
3. Sun XL, Stabler CL, Cazalis CS, Chaikof EL. Carbohydrate and protein immobilization onto solid surfaces by sequential Diels-Alder and azide-alkyne cycloadditions. *Bioconjug Chem* **17**, 52-57 (2006).
4. Dolgosheina EV*, et al.* RNA mango aptamer-fluorophore: a bright, high-affinity complex for RNA labeling and tracking. *ACS Chem Biol* **9**, 2412-2420 (2014)
